# Electrochemical radical cation aza-Wacker cyclizations

**DOI:** 10.3762/bjoc.20.165

**Published:** 2024-08-05

**Authors:** Sota Adachi, Yohei Okada

**Affiliations:** 1 Department of Applied Biological Science, Tokyo University of Agriculture and Technology, 3-5-8 Saiwai-cho, Fuchu, Tokyo 183-8509, Japan

**Keywords:** alkene, aza-Wacker cyclization, electrochemistry, radical cation, sulfonamide

## Abstract

Electrochemical or photochemical single-electron oxidation of bench-stable substrates can generate radical cations that offer unique reactivities as intermediates in various bond-formation processes. Such intermediates can potentially take part in both radical and ionic bond formation; however, the mechanisms involved are complicated and not fully understood. Herein, we report electrochemical radical cation aza-Wacker cyclizations under acidic conditions, which are expected to proceed via radical cations generated by single-electron oxidation of alkenes.

## Introduction

Activating bench-stable substrates is the first step to driving bond formation and/or cleavage. Therefore, the discovery of new modes for activation leads to reaction advancements. Electrochemical [1–5] and photochemical [6–10] reactions that induce single-electron reduction and oxidation are widely used in modern synthetic organic chemistry [11–15]. Single-electron oxidation of bench-stable substrates can generate radical cations that offer unique reactivities as intermediates for various bond-formation processes (also true for reduction). Because the reactivities of radicals and ions are fundamentally different, their creative use may pave the way for complementary bond formation. This merging is unique and such intermediates could potentially take part in both radical and ionic bond formation. However, the mechanisms involved can be complicated and are not fully understood.

**Scheme 1:**
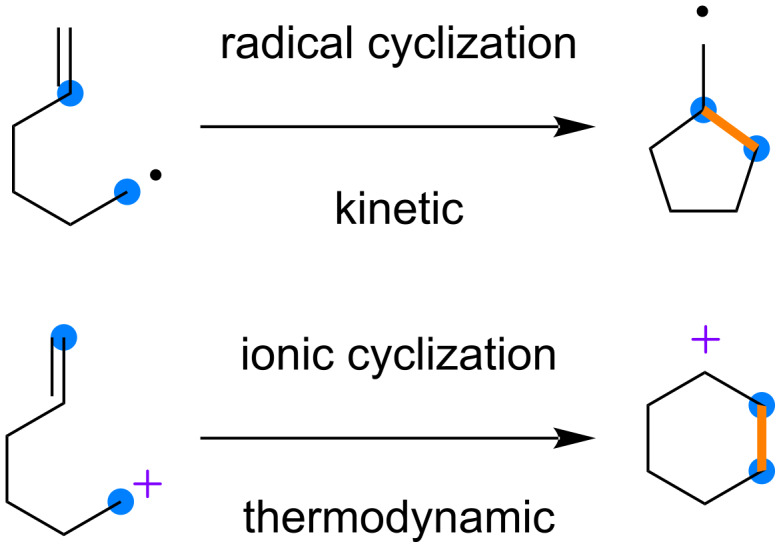
Radical and ionic intramolecular cyclizations.

Alkenes and styrenes are representative radical cation precursors that are widely used to realize the formation of unique bonds. The respective radical cations are trapped by various nucleophiles under radical and/or ion control, where kinetic and/or thermodynamic effects are expected to be dominant. Typical examples that clearly show the difference in such reactivities are intramolecular cyclizations (Scheme 1). A radical cyclization generates a five-membered ring with a less-stable primary radical, while a six-membered ring with a secondary cation is obtained through ionic cyclization. When such intramolecular cyclizations are expected to proceed via radical cations, there are several interpretations of the mechanisms involved, since radical and ionic cyclizations are both possible.

**Scheme 2:**
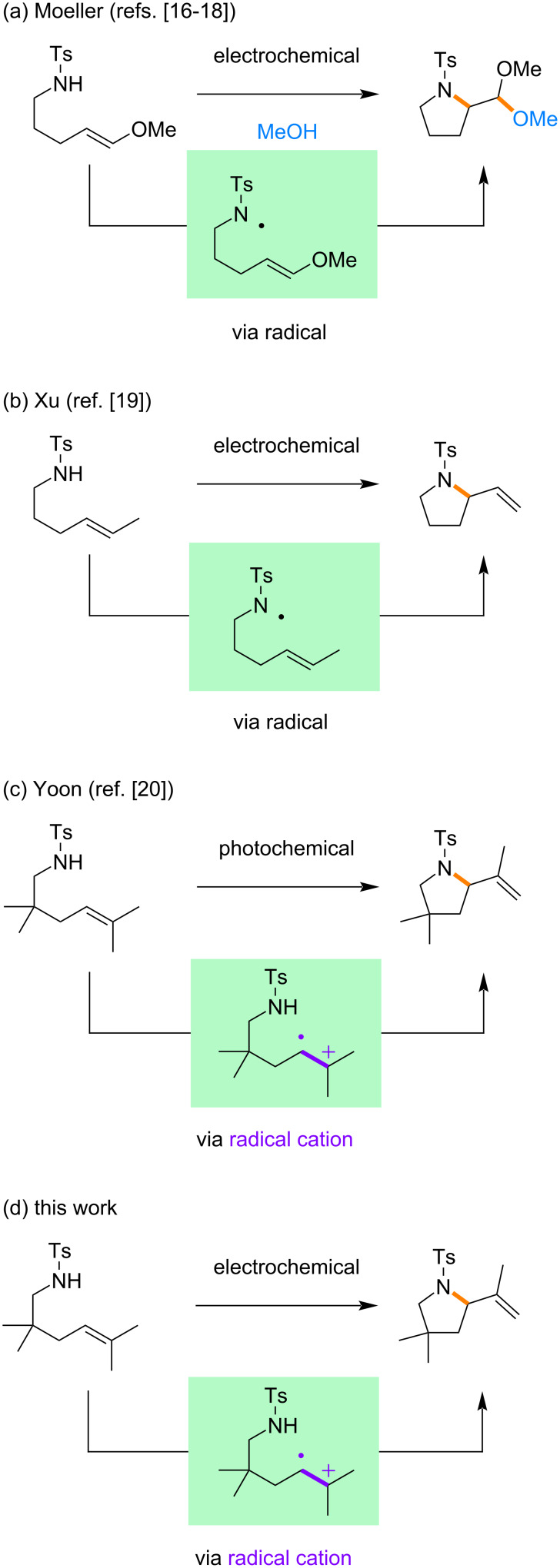
Electrochemical and photochemical aza-Wacker cyclizations.

In this context, electrochemical and photochemical aza-Wacker cyclizations have provided interesting models for mechanistic discussion (Scheme 2). For example, Moeller reported electrochemical reactions under basic conditions, which were proposed to proceed via radicals [16–18]. Xu also reported electrochemical reactions via radicals, which were generated through proton-coupled electron transfer [19]. On the other hand, Yoon reported photochemical reactions under acidic conditions, which were proposed to proceed via radical cations [20]. Since electrochemical and photochemical aza-Wacker cyclizations can offer ring systems that are difficult to construct through state-of-the-art palladium-catalyzed methods, the mechanistic understanding of these cyclizations would be of great help to expand their synthetic utility. Described herein are electrochemical aza-Wacker cyclizations under acidic conditions, which are expected to proceed via radical cations.

## Results and Discussion

The present work began by examining the electrochemical aza-Wacker cyclization using the alkene **1** as a model (Table 1). Based on the conditions reported by Yoon and Moeller, the initial screening was carried out using tetrabutylammonium triflate (Bu_4_NOTf)/1,2-dichloroethane (1,2-DCE) solution. Carbon felt (CF) was used as an anode instead of reticulated vitreous carbon (RVC), with platinum (Pt) as a cathode. To our delight, a constant-current condition at 1 mA was productive, and the desired five-membered pyrrolidine **2** was obtained in high yield (Table 1, entry 2). During the screening of conditions, the addition of acetonitrile (CH_3_CN) was found to be effective, probably due to the increased conductivity of the electrolyte solution (Table 1, entry 1). The reaction did not take place without electricity and most of the starting material was recovered (Table 1, entry 3). The addition of trifluoroacetic acid (TFA) was advantageous in terms of the reproducibility, which was in good accordance with the observation reported by Yoon (Table 1, entry 4). The use of acetic acid (AcOH) instead of TFA gave a slightly lower yield of the five-membered pyrrolidine **2** (Table 1, entry 5). Although a constant-potential condition at 1.8 V was also productive, the constant-current condition gave better results (Table 1, entry 6). Previously, we reported that lithium perchlorate (LiClO_4_)/nitromethane (CH_3_NO_2_) solution was an effective medium to facilitate radical cation reactions [21–25]. However, interestingly, it was not productive for the electrochemical aza-Wacker cyclization (Table 1, entry 7) and the six-membered piperidine **3**, instead of the five-membered pyrrolidine **2**, was obtained in excellent yield without electricity (Table 1, entry 8). Thus, it is proposed that the electrochemical aza-Wacker cyclization under acidic conditions proceeded via radical cations to give five-membered pyrrolidine **2**, while the six-membered piperidine **3** is formed through ionic cyclization under non-electrochemical conditions.

**Table 1:** Control studies for electrochemical aza-Wacker cyclization.^a^

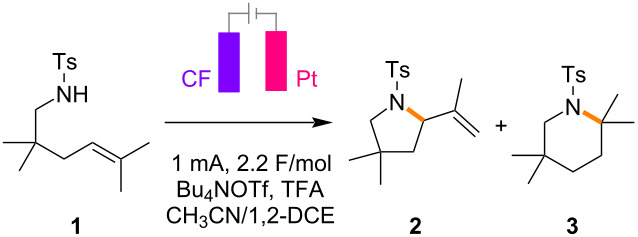		

Entry	Deviation from the optimal condition	Yields of **2** + **3**^b^

1		97 (0) + 0
2	no CH_3_CN	82 (0) + 0
3	no current	0 (75) + 0
4	no TFA	81 (3) + 0
5	AcOH instead of TFA	66 (5) + 0
6	constant potential at 1.8 V	74 (0) + 0
7*^c^*	LiClO_4_/CH_3_NO_2_	0 (0) + 0
8^c^	LiClO_4_/CH_3_NO_2_, no current	0 (0) + 96

^a^Reaction conditions: alkene **1** (0.20 mmol), Bu_4_NOTf (0.1 M), TFA (1 equiv), CH_3_CN (0.4 mL), and 1,2-DCE (3.6 mL). ^b^Determined by NMR analysis. The recovered starting material is reported in parentheses. ^c^LiClO_4_ (1 M) instead of Bu_4_NOTf (0.1 M).

**Scheme 3:**
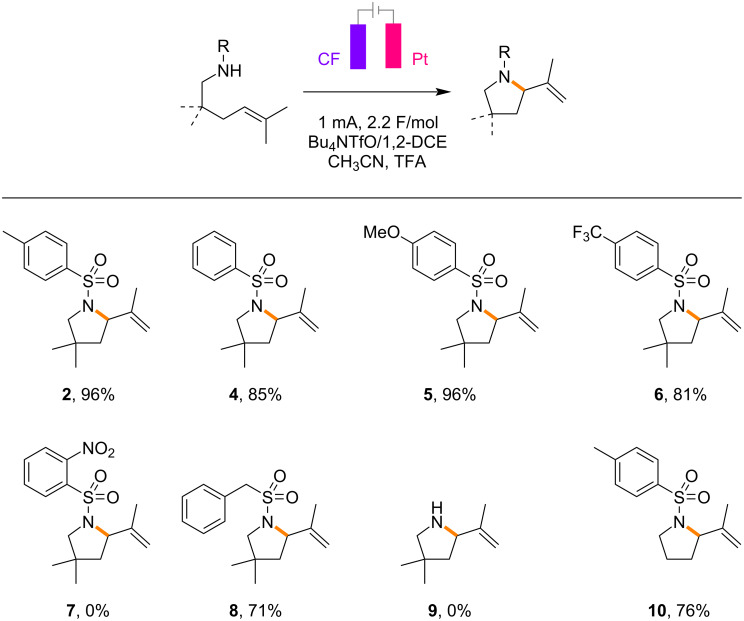
Scope of electrochemical aza-Wacker cyclization. Reaction conditions: the alkene (0.20 mmol), Bu_4_NOTf (0.1 M), TFA (1 equiv), CH_3_CN (0.4 mL), and 1,2-DCE (3.6 mL). Yields reported here are isolated yields.

With the optimized conditions in hand, the scope of the electrochemical aza-Wacker cyclization was investigated (Scheme 3). Various aryl sulfonamides **4**–**6** were compatible to give the respective five-membered pyrrolidines, except for that possessing a 2-nitro group **7**. As discussed later with cyclic voltammetric studies, the electron density in the aryl rings does not seem to have a significant impact on the reaction. While benzyl sulfonamide **8** was productive under the optimized conditions, unprotected amine **9** was not compatible. Although *gem*-dimethyl groups installed at the tether should have a positive impact on intramolecular cyclization, they were not essential for the reaction (**10**).

**Scheme 4:**
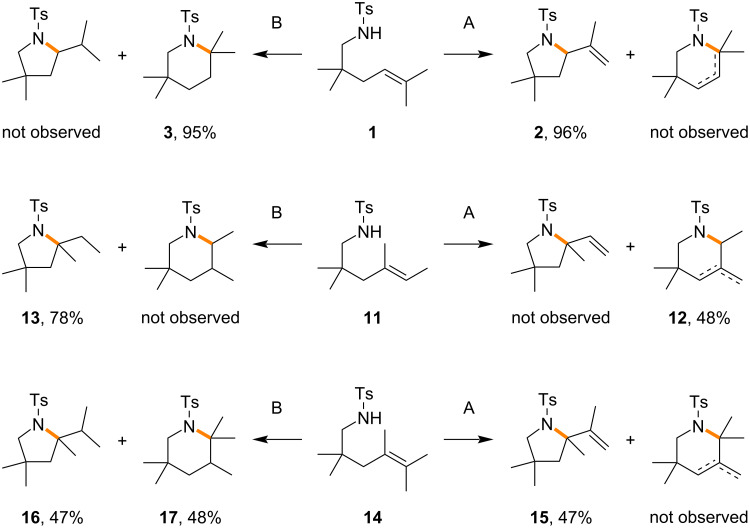
Mechanistic studies of aza-Wacker cyclization. A: Electrochemical (Bu_4_NOTf in CH_3_CN/1,2-DCE), B: non-electrochemical (LiClO_4_ in CH_3_NO_2_).

In order to obtain mechanistic insight into the aza-Wacker cyclization, differently substituted alkenes **11**, **14** were prepared and subjected to the reaction under electrochemical and non-electrochemical conditions (Scheme 4). In the case of the trisubstituted alkene **11**, the six-membered anti-Markovnikov product **12** was selectively obtained under electrochemical conditions, while the five-membered Markovnikov product **13** was obtained in good yield under non-electrochemical conditions. In the case of the tetrasubstituted alkene **14**, the five-membered pyrrolidine **15** was selectively obtained under electrochemical conditions, while both the five-membered pyrrolidine **16** and six-membered piperidine **17** were obtained in good mass balance under non-electrochemical conditions. Although the detailed mechanism remains an open question, the electrochemical aza-Wacker cyclizations might be radical reactions rather than ionic ones, since the six-membered piperidine was not obtained from the tetrasubstituted alkene **14**.

**Figure 1:**
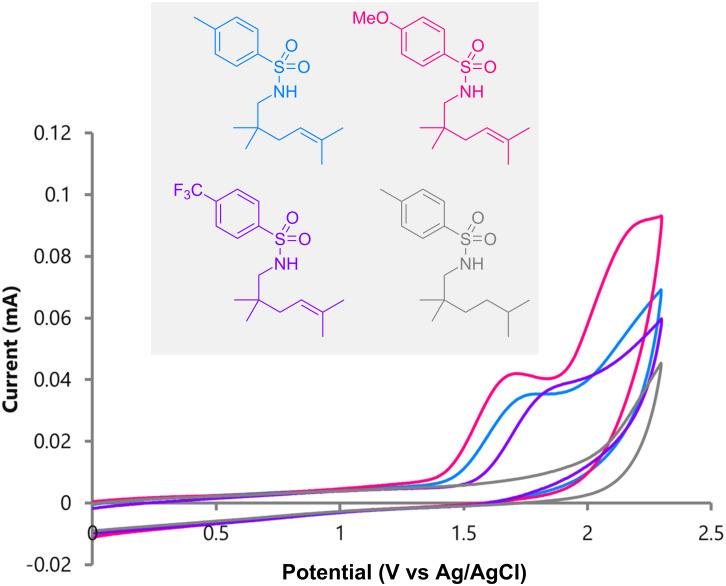
Cyclic voltammograms for aryl sulfonamides.

Cyclic voltammetric studies have provided further mechanistic insights into electrochemical aza-Wacker cyclizations. As reported by Yoon, a trisubstituted alkene is oxidized at significantly lower potential than aryl sulfonamides, suggesting that the reactions were initiated by single-electron oxidation of the alkenes. Although a drop in oxidation potential for the alkene was observed when tethered to an aryl sulfonamide, as detailed by Moeller, rapid intramolecular cyclization would be the key [26–28]. We also measured cyclic voltammograms for aryl sulfonamides with and without trisubstituted alkenes (Figure 1). As described above, the electron density in the aryl rings does not seem to have a significant impact on the reaction, since alkenes possessing methyl **2**, methoxy **5**, and trifluoromethyl **6** groups were all high yielding. This observation was supported by the cyclic voltammetric studies, namely, their oxidation potentials were similar. This suggests that the reactions are initiated by single-electron oxidation of alkenes instead of aryl sulfonamides, leading to unique radical cation aza-Wacker cyclizations. The cyclic voltammogram of the aryl sulfonamide without a trisubstituted alkene provides clear-cut experimental evidence of this, since the oxidation potential was recorded at a much higher value.

## Conclusion

In conclusion, we have demonstrated that electrochemical aza-Wacker cyclizations are enabled under acidic conditions, and are expected to proceed via radical cations. Synthetic outcomes and cyclic voltammetric studies suggest that the reactions are initiated by single-electron oxidation of the alkenes instead of the aryl sulfonamides. Although the detailed mechanism remains an open question, the electrochemical radical cation aza-Wacker cyclizations might be radical reactions rather than ionic ones, since five-membered pyrrolidine formation is preferred over six-membered piperidine formation. Further mechanistic studies of the electrochemical radical cation aza-Wacker cyclizations are underway in our laboratory.

## Experimental

Electrochemical aza-Wacker cyclizations: The appropriate alkene (0.20 mmol), TFA (0.20 mmol), and CH_3_CN (0.4 mL) were added to a solution of Bu_4_NOTf/1,2-DCE (0.10 M, 3.6 mL) while stirring at room temperature. The resulting reaction mixture was electrolyzed at 1 mA using a CF anode (10 mm × 10 mm) and a Pt cathode (10 mm × 20 mm) in an undivided cell with stirring. The solution was diluted with water and extracted with dichloromethane. The combined organic layers were dried over Na_2_SO_4_, filtered, and concentrated in vacuo. Yields were determined by ^1^H NMR analysis using benzaldehyde as an internal standard (Table 1). Silica gel column chromatography (hexane/ethyl acetate) gave the corresponding ring compounds.

## Supporting Information

File 1 General remarks and characterization data, including copies of ^1^H and ^13^C NMR spectra.

## Data Availability

All data that supports the findings of this study is available in the published article and/or the supporting information to this article.
