# Supplementary material for: Electrochemical radical cation aza-Wacker cyclizations
Source: Beilstein J Org Chem. 2024 Aug 5;20:1900–5. doi: 10.3762/bjoc.20.165 (PMC11318630; doi:10.3762/bjoc.20.165)

BEILSTEIN JOURNAL OF ORGANIC CHEMISTRY

# Supporting Information

for

# Electrochemical radical cation aza-Wacker cyclizations

Sota Adachi and Yohei Okada

*Beilstein J. Org. Chem.* **2024,** *20,* 1900–1905. doi:10.3762/bjoc.20.165

## General remarks and characterization data, including copies of $^{1}H$ and $^{13}C$ NMR spectra

License and Terms: This is a supporting information file under the terms of the Creative Commons Attribution License (https://creativecommons.org/licenses/by/4.0). Please note that the reuse, redistribution and reproduction in particular requires that the author(s) and source are credited and that individual graphics may be subject to special legal provisions.
The license is subject to the *Beilstein Journal of Organic Chemistry* terms and conditions: (https://www.beilstein-journals.org/bjoc/terms)

**Table of contents**

1. General remarks ........................................................................................................ S2

2. Synthetic procedures, characterization data ........................................................................ S3

3. Cyclic voltammetry studies ........................................................................................ S23

4. Copies of $^{1}H$ and $^{13}C$ NMR spectra ........................................................................ S25

## 1. General remarks

All reagents and solvents were purchased from commercial sources and used without further purification. Reactions were monitored by thin-layer chromatography (TLC) carried out on silica gel plates, with detection by UV absorption (254 nm) and by heating the plates after dipping them in a solution of 12 M molybdo(VI) phosphoric acid *n*-hydrate in 95% ethanol. Silica gel (particle size 40–50 μm, normal or reversed-phase) was used for column chromatography. $^{1}$H NMR spectra were collected on a 600, 500, or 400 MHz NMR spectrometer using the deuterated solvent as an internal deuterium reference. Chemical shift data are given in δ units calibrated with residual protic solvent. The multiplicity of a signal is indicated as follows: s, singlet; d, doublet; t, triplet; q, quartet; quint, quintet; sext, sextet; sept, septet; m, multiplet. $^{13}$C NMR spectra were collected at 150, 125, or 100 MHz with proton decoupling using the deuterated solvent as an internal carbon reference. Chemical shift data are given in *δ* units calibrated with residual solvent. High-resolution mass spectra (HRMS) were collected by electrospray ionization (ESI)- or direct analysis in real time (DART)-time-of-flight (TOF) spectrometers.

## 2. Synthetic procedure, characterization data

**S1**, **S2**–**S10**, **S12**, **S13**, **1**, and **11** were synthesized in a similar manner as previously reported by Yoon and co-workers.[1]

*Synthesis of 2,2,5-trimethylhex-4-enenitrile (**S1**) Yellow oil.*

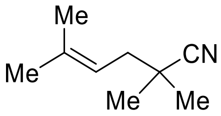


To a solution of $iPr_2NH$ (2.26 mL, 16.0 mmol) in THF (32 mL) stirred at 0 °C was added dropwise *n*-BuLi in hexane (1.52 M, 10.5 mL, 16.0 mmol). The resulting solution was stirred at 0 °C for 15 min, then, isobutyronitrile (1.45 mL, 16.0 mmol) was added dropwise, and the resulting solution was stirred at 0 °C for 15 min. Prenyl bromide (1.85 mL, 16.0 mmol) was added dropwise and the resulting reaction mixture was stirred at rt for 18 h. The reaction was quenched by sat. $NH_4Cl$ aq. (80 mL) and extracted by $Et_2O$ (40 mL × 3). The combined organic extracts were washed with water and brine, dried over $Na_2SO_4$, filtered, and concentrated in vacuo to give crude **S1** (2.2 g) which was used without further purification.

*Synthesis of 2,2,5-trimethylhex-4-en-1-amine (**S2**) Colorless oil.*

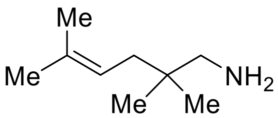


To a solution of $LiAlH_4$ (1.14 g, 30.0 mmol) in $Et_2O$ (40 mL) stirred at 0 °C was slowly added **S1** (2.2 g) and the resulting reaction mixture was stirred at rt for 2 h. The consumption of starting materials was checked by TLC, the reaction mixture was cooled to 0 °C, and quenched by dropwise addition of 3 M NaOH aq. (10 mL).

The resulting mixture was stirred for 15 min, dried over $Na_2SO_4$, filtered, and concentrated in vacuo. Silica gel column chromatography (DCM/MeOH 20:1) gave the titled compound in 87% (**S2**, 1.98 g, 14.0 mmol).

$^1$H NMR (600 MHz, $CDCl_3$) $\delta$ 5.18-5.15 (1H, m), 2.44 (2H, s), 1.89 (2H, d, $J$ = 7.6 Hz), 1.72 (3H, s), 1.61 (3H, s), 1.20 (2H, m), 0.84 (6H, s); $^{13}$C{$^1$H} NMR (150 MHz, $CDCl_3$) $\delta$ 133.0, 120.8, 52.8, 37.7, 35.8, 26.1, 24.7, 17.9; HRMS [M + H]$^+$ calculated for $[C_9H_{20}N]^+$ 142.1590, found 142.1586.

The characterization data is compatible with the reported values.[1]

*Synthesis of 4-methyl-N-(2,2,5-trimethylhex-4-en-1-yl)benzenesulfonamide (**1**) White solid.*

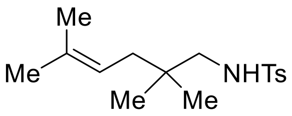


To a solution of **S2** (1.00 g, 7.08 mmol) and $Et_3N$ (1.48 mL, 10.6 mmol) in DCM (24 mL) stirred at 0 °C was added TsCl (1.48 g, 7.76 mmol). After stirring the resulting reaction mixture at rt for 2 h, the consumption of starting materials was checked by TLC, and the solution was concentrated in vacuo. Silica gel column chromatography (*n*-hexane/EtOAc 10:1) gave the titled compound in 84% (**1**, 1.76 g, 6.0 mmol).

$^1$H NMR (300 MHz, $CDCl_3$) $\delta$ 7.77 (2H, d, $J$ = 8.3 Hz), 7.31 (2H, d, $J$ = 7.9 Hz), 5.03 (1H, tt, $J$ = 7.9, 1.4 Hz), 4.83 (1H, t, $J$ = 6.5 Hz), 2.66 (2H, d, $J$ = 6.9 Hz), 2.43 (3H, s), 1.86 (2H, d, $J$ = 7.6 Hz), 1.67 (3H, s), 1.54 (3H, s), 0.83 (6H, s); $^{13}$C{$^1$H} NMR (100 MHz, $CDCl_3$) $\delta$ 143.3, 137.0, 134.1, 129.7, 127.1, 119.8, 53.1, 37.9, 34.9, 26.0, 24.9, 21.6, 17.9; HRMS [M + H]$^+$ calculated for $[C_{16}H_{26}NO_2S]^+$ 296.1679, found 296.1700.

The characterization data is compatible with the reported values.[1]

*Synthesis of N-(2,2,5-trimethylhex-4-en-1-yl)benzenesulfonamide (**S3**) Colorless oil.*

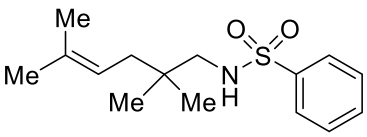


To a solution of **S2** (0.500 g, 3.54 mmol) and $Et_3N$ (0.740 mL, 5.31 mmol) in DCM (12 mL) stirred at 0 °C was added benzenesulfonyl chloride (0.498 mL, 3.89 mmol). After stirring the resulting reaction mixture at rt for 3.5 h, the consumption of starting materials was checked by TLC, and the solution was concentrated in vacuo. Silica gel column chromatography (*n*-hexane/EtOAc 10:1) gave the titled compound in 91% (**S3**, 905 mg, 3.2 mmol).

$^1$H NMR (300 MHz, $CDCl_3$) $\delta$ 7.88-7.84 (2H, m), 7.61-7.49 (3H, m), 5.05 (1H, m), 4.44 (1H, m), 2.70 (2H, d, $J$ = 6.9 Hz), 1.87 (2H, d, $J$ = 7.6 Hz), 1.68 (3H, s), 1.55 (3H, s), 0.84 (6H, s); $^{13}$C{$^1$H} NMR (100 MHz, $CDCl_3$) $\delta$ 140.1, 134.2, 132.6, 129.1, 127.1, 119.8, 53.1, 37.9, 35.0, 26.1, 24.9, 18.0; HRMS $[M + H]^+$ calculated for $[C_9H_{20}N]^+$ 142.1590, found 142.1586.

The characterization data is compatible with the reported values.[1]

*Synthesis of 4-methoxy-N-(2,2,5-trimethylhex-4-en-1-yl)benzenesulfonamide (**S4**) White solid.*

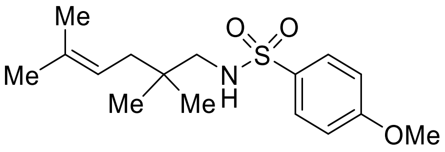


To a solution of **S2** (0.500 g, 3.54 mmol) and $Et_3N$ (0.740 mL, 5.31 mmol) in DCM (12 mL) stirred at 0 °C was added 4-methoxybenzenesulfonyl chloride (0.802 g, 3.89 mmol). After stirring the resulting reaction mixture at rt for 2 h, the consumption of starting materials was checked by TLC, and the solution was

concentrated in vacuo. Silica gel column chromatography (*n*-hexane/EtOAc 7:1) gave the titled compound in 94% (**S4** 1.03 g, 3.3 mmol).

$^1$H NMR (300 MHz, $CDCl_3$) $\delta$ 7.79 (2H, d, $J$ = 8.9 Hz), 6.98 (2H, d, $J$ = 8.9 Hz), 5.04 (1H, m), 4.49 (1H, t, $J$ = 6.9 Hz), 3.87 (3H, s), 2.67 (2H, d, $J$ = 6.9 Hz), 1.86 (2H, d, $J$ = 7.6 Hz), 1.67 (3H, s), 1.55 (3H, s), 0.83 (6H, s); $^{13}C\{^1H\}$ NMR (100 MHz, $CDCl_3$) $\delta$ 162.7, 133.9, 131.6, 129.1, 119.8, 114.1, 55.6, 53.0, 37.8, 34.9, 26.0, 24.8, 17.8; HRMS $[M + H]^+$ calculated for $[C_{16}H_{26}NO_3S]^+$ 312.1628, found 312.1614.

The characterization data is compatible with the reported values.[1]

*Synthesis of 4-(trifluoromethyl)-N-(2,2,5-trimethylhex-4-en-1-yl)benzenesulfonamide (**S5**) White solid.*

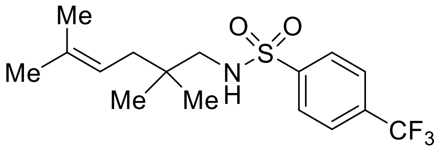


To a solution of **S2** (0.500 g, 3.54 mmol) and $Et_3N$ (0.740 mL, 5.31 mmol) in DCM (12 mL) stirred at 0 °C was added 4-(trifluoromethyl)benzenesulfonyl chloride (0.949 g, 3.89 mmol). After stirring the resulting reaction mixture at rt for 2 h, the consumption of starting materials was checked by TLC, and the solution was concentrated in vacuo. Silica gel column chromatography (*n*-hexane/EtOAc 10:1) gave the titled compound in 91% (**S5**, 1.13 g, 3.23 mmol).

$^1$H NMR (300 MHz, $CDCl_3$) $\delta$ 7.98 (2H, d, $J$ = 8.3 Hz), 7.80 (2H, d, $J$ = 8.3 Hz), 5.05 (1H, tt, $J$ = 1.7, 8.4 Hz), 4.47 (1H, t, $J$ = 6.2 Hz), 2.74 (2H, d, $J$ = 6.5 Hz), 1.88 (2H, d, $J$ = 7.9 Hz), 1.68 (3H, s), 1.56 (3H,s), 0.86 (6H, s); $^{13}C\{^1H\}$ NMR (100 MHz, $CDCl_3$) $\delta$ 143.7, 134.4, 127.6, 126.3, 126.3, 119.5, 53.2, 37.9, 35.0, 26.0, 24.9, 17.9; HRMS $[M + H]^+$ calculated for $[C_{16}H_{23}F_3NO_2S]^+$ 350.1396, found 350.1389.

The characterization data is compatible with the reported values.[1]

*Synthesis of 2-nitro-N-(2,2,5-trimethylhex-4-en-1-yl)benzenesulfonamide (**S6**) Colorless oil.*

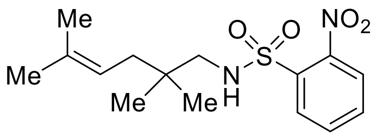


To a solution of **S2** (0.500 g, 3.54 mmol) and $Et_3N$ (0.740 mL, 5.31 mmol) in DCM (12 mL) stirred at 0 °C was added 2-NsCl (0.862 g, 3.89 mmol). After stirring the resulting reaction mixture at rt for 1 h, the consumption of starting materials was checked by TLC, and the solution was removed by vacuo. Silica gel column chromatography (*n*-hexane/EtOAc 7:1) gave the titled compound in 80% (**S6**, 0.920 g, 2.8 mmol).

$^1$H NMR (400 MHz, $CDCl_3$) $\delta$ 8.13-8.10 (1H, m), 7.87-7.85 (1H, m), 7.76-7.73 (2H, m), 5.30 (1H, t, $J$ = 6.4 Hz), 5.12-5.08 (1H, m), 2.84 (2H, d, $J$ = 6.9 Hz), 1.93 (2H, d, $J$ = 7.8 Hz), 1.70 (3H, s), 1.59 (3H,s), 0.89 (6H, s); $^{13}$C{$^1$H} NMR (100 MHz, $CDCl_3$) $\delta$ 148.1, 134.5, 133.7, 133.5, 132.8, 131.1, 125.4, 119.5, 53.5, 37.9, 35.1, 26.1, 25.0, 17.9; HRMS [M + H]$^+$ calculated for $[C_{15}H_{23}N_2O_4S]^+$ 327.1373, found 327.1392.

The characterization data is compatible with the reported values.[1]

*Synthesis of 1-phenyl-N-(2,2,5-trimethylhex-4-en-1-yl)methanesulfonamide (**S7**) White solid.*

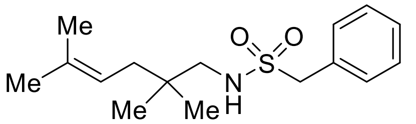


To a solution of **S2** (0.500 g, 3.54 mmol) and $Et_3N$ (0.740 mL, 5.31 mmol) in DCM (12 mL) stirred at 0 °C was added benzylsulfonyl chloride (0.742 g, 3.89 mmol). After stirring the resulting reaction mixture at rt for 1 h, the consumption of starting materials was checked by TLC, and the solution was removed by vacuo. Silica gel column chromatography (*n*-hexane/EtOAc 10:1) gave the titled compound in 84% (**S7**, 875 mg, 2.96 mmol).

$^{1}H$ NMR (400 MHz, $CDCl_3$) $\delta$ 7.40-7.36 (5H, m), 5.10-5.06 (1H, m), 4.38 (1H, t, $J$ = 6.4 Hz), 4.23 (2H, s), 2.75 (2H, d, $J$ = 6.4 Hz), 1.86 (2H, d, $J$ = 7.3 Hz), 1.69 (3H, s), 1.54 (3H,s), 0.85 (6H, s); $^{13}C\{^{1}H\}$ NMR (100 MHz, $CDCl_3$) $\delta$ 134.0, 130.6, 129.5, 128.8, 128.7, 119.7, 58.5, 53.5, 37.8, 35.1, 26.0, 24.7, 17.9; HRMS [M + H]$^+$ calculated for $[C_{16}H_{26}NO_2S]^+$ 296.1679, found 296.1650.

The characterization data is compatible with the reported values.[1]

*Synthesis of 5-methylhex-4-enenitrile (**S8**) Yellow oil.*

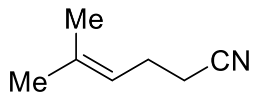

To a solution of MeCN (0.370 mL, 7.04 mmol) in THF (18 mL) stirred at −78 °C was added dropwise *n*-BuLi in hexane (1.52 M, 4.77 mL, 7.25 mmol). The resulting solution was stirred at −78 °C for 15 min, prenyl bromide (1.00 mL, 8.66 mmol) in THF (9 mL) was added dropwise and the resulting reaction mixture was stirred at rt for 2 h. The reaction was quenched by sat. $NH_4Cl$ aq. (50 mL) and extracted by $Et_2O$ (40 mL × 3). The combined organic extracts were washed with water and brine, dried over $Na_2SO_4$, filtered, and concentrated in vacuo to give crude **S8** (2.2 g) which was used without further purification.

*Synthesis of 5-methylhex-4-en-1-amine (**S9**) Yellow oil.*

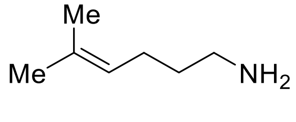

To a solution of $LiAlH_4$ (1.29 g, 33.9 mmol) in $Et_2O$ (75 mL) stirred at 0 °C was slowly added **S8** (2.2 g) in $Et_2O$. After stirring the resulting reaction mixture at rt for 2 h the consumption of starting materials was checked by TLC, the reaction mixture was cooled to 0 °C, and quenched by dropwise addition of 3 M aq.

NaOH (10 mL). The resulting mixture was stirred 15 min, dried over $Na_2SO_4$, filtered, and concentrated in vacuo to give crude **S9** (1.3 g) which was used without further purification.

*Synthesis of 4-methyl-N-(5-methylhex-4-en-1-yl)benzenesulfonamide (**S10**) Colorless oil.*

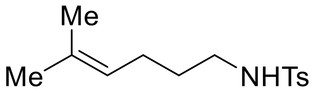


To a solution of **S9** (1.3 g) and $Et_3N$ (2.40 mL, 17.2 mmol) in DCM (30 mL) stirred at 0 °C was added TsCl (2.68 g, 14 mmol). After stirring the resulting reaction mixture at rt for 2 h, the consumption of starting materials was checked by TLC, and the solution was concentrated in vacuo. Silica gel column chromatography (*n*-hexane/EtOAc 10:1) gave the titled compound in 58% (**S10**, 1.09 g, 4.1 mmol) over 3 steps.

$^1$H NMR (600 MHz, $CDCl_3$) $\delta$ 7.77 (2H, d, $J$ = 8.9 Hz), 7.30 (2H, d, $J$ = 7.6 Hz), 5.21-5.15 (1H, m), 4.98 (1H, t, $J$ = 6.9 Hz), 2.91 (2H, q, $J$ = 6.9 Hz), 2.42 (3H, s), 1.94 (2H, q, $J$ = 7.6 Hz), 1.64 (3H, s), 1.52 (3H,s), 1.48 (2H, quint, $J$ = 7.6 Hz); $^{13}$C{$^1$H} NMR (150 MHz, $CDCl_3$) $\delta$ 143.3, 137.1, 132.6, 129.7, 127.2, 123.2, 43.0, 29.6, 25.7, 25.1, 21.6, 17.7; HRMS $[M + H]^+$ calculated for $[C_{14}H_{22}NO_2S]^+$ 268.1366, found 268.1390. The characterization data is compatible with the reported values.[1]

*Synthesis of (E)-1-Bromo-2-methylbut-2-ene (**S11**). Colorless oil.*

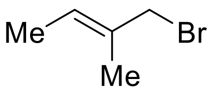


Synthesized according to the method reported by Alexanian and co-workers.[2] The characterization data is compatible with the reported values.[3]

*Synthesis of (E)-2,2,4-trimethylhex-4-enenitrile (**S12**) Yellow oil.*

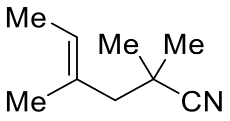


To a solution of $iPr_2NH$ (1.13 mL, 8.00 mmol) in THF (20 mL) stirred at 0 °C was added dropwise *n*-BuLi in hexane (1.52 M, 5.25 mL, 8.00 mmol). The resulting solution was stirred at 0 °C for 15 min, isobutyronitrile (0.725 mL, 8.00 mmol) was added dropwise, and the resulting solution was stirred at 0 °C for 15 min. Then, **S11** (1.19 g, 8.00 mmol) in THF (5 mL) was added dropwise and the resulting reaction mixture was stirred at rt for 18 h. The reaction was quenched by sat. $NH_4Cl$ aq. (40 mL) and extracted by $Et_2O$ (40 mL × 3). The combined organic extracts were washed with water and brine, dried over $Na_2SO_4$, filtered, and concentrated in vacuo to give crude **S12** (1.3 g) which was used without further purification.

*Synthesis of (E)-2,2,4-trimethylhex-4-en-1-amine (**S13**) Colorless oil.*

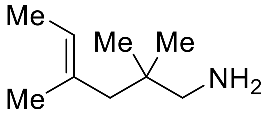


To a solution of $LiAlH_4$ (0.500 g, 13.2 mmol) in THF (20 mL) stirred at 0 °C was slowly added **S12** (1.3 g). After stirring the resulting reaction mixture at rt for 2 h, the consumption of starting materials was checked by TLC, the reaction mixture was cooled to 0 °C, and quenched by dropwise addition of 3 M aq. NaOH (10 mL). The resulting mixture was stirred 15 min, dried over $Na_2SO_4$, filtered, and concentrated in vacuo to give crude **S13** (1.0 g) which was used without further purification.

*Synthesis of (E)-4-methyl-N-(2,2,4-trimethylhex-4-en-1-yl)benzenesulfonamide (**11**) White solid.*

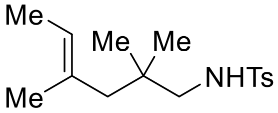


To a solution of **S13** (1.0 g) and $Et_3N$ (1.48 mL, 10.6 mmol) in DCM (24 mL) stirred at 0 °C was added TsCl (1.48 g, 7.76 mmol). After stirring the resulting reaction mixture at rt for 2 h, the consumption of starting materials was checked by TLC, and the solution was concentrated in vacuo. Silica gel column chromatography (*n*-hexane/EtOAc 10:1) gave the titled compound in 34% (**11**, 800 mg, 2.7 mmol, *cis*/*trans* = 1:15) over 3 steps.

$^1$H NMR (600 MHz, $CDCl_3$) $\delta$ 7.73 (2H, d, $J$ = 8.3 Hz), 7.31 (2H, d, $J$ = 8.3 Hz), 5.12 (1H, q, $J$ = 7.6 Hz), 4.46 (1H, t, $J$ = 6.9 Hz), 2.68 (2H, d, $J$ = 6.9 Hz), 2.43 (3H, s), 1.89 (2H, s), 1.60 (3H, s), 1.54 (3H,d, $J$ = 6.9 Hz), 0.85 (6H, s); $^{13}$C{$^1$H} NMR (150 MHz, $CDCl_3$) $\delta$ 143.4, 137.0, 132.8, 129.7, 127.2, 123.5, 53.5, 49.5, 35.1, 25.7, 21.6, 18.6, 13.7; HRMS $[M + H]^+$ calculated for $[C_{16}H_{26}NO_2S]^+$ 296.1679, found 296.1671.

The characterization data is compatible with the reported values.[1]

*Synthesis of 1-bromo-2,3-dimethylbut-2-ene (**S14**) Colorless oil.*

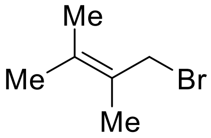


Synthesized according to the method reported by Zhang and co-workers.[3] The characterization data is compatible with the reported values.[3]

All spectral data were in agreement with previously reported values.

*Synthesis of 2,2,4,5-tetramethylhex-4-enenitrile (**S15**) Yellow oil.*

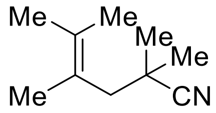


To a solution of $iPr_2NH$ (1.13 mL, 8.00 mmol) in THF (16 mL) stirred at 0 °C was added dropwise *n*-BuLi in hexane (1.52 M, 5.25 mL, 8.00 mmol). The resulting solution was stirred at 0 °C for 15 min, isobutyronitrile (0.725 mL, 8.00 mmol) was added dropwise, and the resulting solution was stirred at 0 °C for 15 min. Then, **S14** (1.30 g, 8.00 mmol) in THF (5 mL) was added dropwise and the resulting reaction mixture was stirred at rt for 18 h. The reaction mixture was quenched by sat. $NH_4Cl$ aq. (40 mL) and extracted by $Et_2O$ (40 mL × 3). The combined organic extracts were washed with water and brine, dried over $Na_2SO_4$, filtered, and concentrated in vacuo to give crude **S15** (0.46 g) which was used without further purification.

*Synthesis of 2,2,4,5-tetramethylhex-4-en-1-amine (**S16**) Yellow oil.*

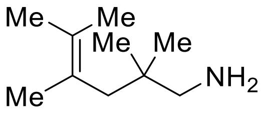


To a solution of $LiAlH_4$ (171 mg, 4.50 mmol) in THF (20 mL) stirred at 0 °C was slowly added **S15** (0.46 g) in THF (5 mL). After stirring the resulting reaction mixture at rt for 2 h, the consumption of starting materials was checked by TLC, the reaction mixture was cooled to 0 °C, and quenched by dropwise addition of 3 M NaOH aq. (10 mL). The resulting mixture was stirred 15 min, dried over $Na_2SO_4$, filtered, and concentrated in vacuo to give crude **S16** (0.47 g) which was used without further purification.

*Synthesis of 4-methyl-N-(2,2,4,5-tetramethylhex-4-en-1-yl)benzenesulfonamide (**14**) White solid.*

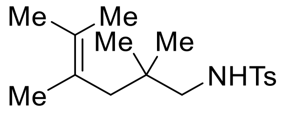


To a solution of **S16** (0.47 g) and $Et_3N$ (0.630 mL, 4.50 mmol) in DCM (12 mL) stirred at 0 °C was added TsCl (0.630 g, 3.30 mmol). After stirring the resulting reaction mixture at rt for 2 h, the consumption of starting materials was checked by TLC, and the solution was removed by vacuo. Silica gel column chromatography (*n*-hexane/EtOAc 10:1) gave the titled compound in 20% (**14**, 491 mg, 1.6 mmol) over 3 steps.

$^1$H NMR (600 MHz, $CDCl_3$) $\delta$ 7.74 (2H, d, $J$ = 8.3 Hz), 7.31 (2H, d, $J$ = 7.6 Hz), 4.74 (1H, t, $J$ = 6.2 Hz), 2.70 (2H, d, $J$ = 6.9 Hz), 2.43 (3H, s), 2.00 (2H, s), 1.63 (6H, s), 1.57 (3H,s), 0.87 (6H, s); $^{13}$C{$^1$H} NMR (150 MHz, $CDCl_3$) $\delta$ 143.3, 137.1, 129.7, 128.4, 127.2, 125.0, 54.4, 44.4, 36.7, 25.7, 21.8, 21.7, 21.6, 21.0; HRMS $[M + H]^+$ calculated for $[C_{17}H_{28}NO_2S]^+$ 310.1835, found 310.1860.

*Synthesis of 2,2,5-trimethylhexanenitrile (**S17**) Yellow oil.*

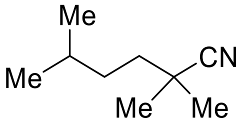


To a solution of $iPr_2NH$ (2.26 mL, 16.0 mmol) in THF (32 mL) stirred at 0 °C was added dropwise *n*-BuLi in hexane (1.52M, 10.5 mL, 16.0 mmol). The resulting solution was stirred at 0 °C for 15 min, isobutyronitrile (1.45 mL, 16.0 mmol) was added dropwise, and the resulting solution was stirred at 0 °C for 15 min. A solution of 1-bromo-3-methylbutane (2.01 mL, 16.0 mmol) in THF (2 mL) was added dropwise and the resulting reaction mixture was stirred at rt for 18 h. The reaction was quenched by sat. $NH_4Cl$ aq. (80 mL) and extracted by $Et_2O$ (40 mL × 3). The combined organic extracts were washed with water and brine, dried over $Na_2SO_4$, filtered, and concentrated in vacuo to give crude **S17** (3.1 g) which was used without further purification.

*Synthesis of 2,2,5-trimethylhexan-1-amine (**S18**) Yellow oil.*

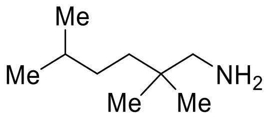

To a solution of $LiAlH_4$ (1.34 g, 35.2 mmol) in $Et_2O$ (40 mL) stirred at 0 °C was slowly added **S17** (3.1 g). After stirring the resulting reaction mixture at rt for 2 h, the consumption of starting materials was checked by TLC, the reaction mixture was cooled to 0 °C, and quenched by dropwise addition of 3 M NaOH aq. (10 mL). The resulting mixture was stirred 15 min, dried over $Na_2SO_4$, filtered, and concentrated in vacuo to give crude **S18** (2.4 g) which was used without further purification.

*Synthesis of 4-methyl-N-(2,2,5-trimethylhexyl)benzenesulfonamide (**S19**) White solid.*

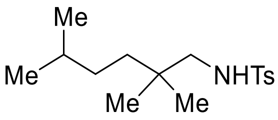

To a solution of **S18** (2.4 g) and $Et_3N$ (3.35 mL, 24.0 mmol) in DCM (30 mL) stirred at 0 °C was added TsCl (3.66 g, 19.2 mmol). After stirring the resulting reaction mixture at rt for 2 h, the consumption of starting materials was checked by TLC, and the solvent was removed by vacuo. Silica gel column chromatography (*n*-hexane/EtOAc 10:1) gave the titled compound in 49% (**S19**, 2.32 g, 7.8 mmol) over 3 steps.

$^1$H NMR (400 MHz, $CDCl_3$) $\delta$ 7.76 (2H, d, $J$ = 8.2 Hz), 7.31 (2H, d, $J$ = 8.2 Hz), 4.73 (1H, t, $J$ = 6.4 Hz), 2.66 (2H, d, $J$ = 6.9 Hz), 2.43 (3H, s), 1.37 (1H, sept, $J$ = 6.4 Hz), 1.16-1.12 (2H, m), 1.03-0.97 (2H, m), 0.83-0.82 (12H, m); $^{13}$C{$^1$H} NMR (100 MHz, $CDCl_3$) $\delta$ 143.3, 137.1, 129.7, 127.2, 53.1, 37.2, 33.7, 32.7, 28.7, 24.9, 22.7, 21.6; HRMS $[M + H]^+$ calculated for $[C_{16}H_{28}NO_2S]^+$ 298.1835, found 298.1856.

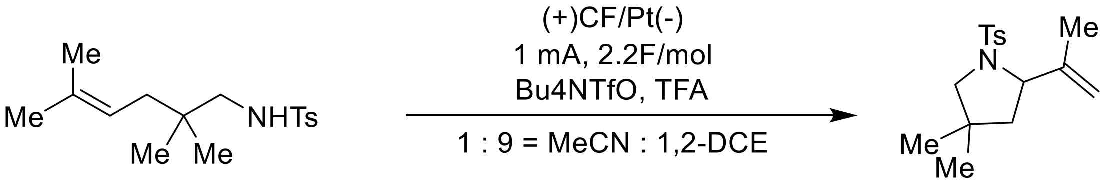


*General procedure for electrochemical aza-Wacker cyclizations.* The appropriate alkene (0.20 mmol), TFA (0.20 mmol), and $CH_3CN$ (0.4 mL) were added to a solution of $Bu_4NOTf$/1,2-DCE (0.10 M, 3.6 mL) while stirring at room temperature. The resulting reaction mixture was electrolyzed at 1 mA using CF anode (10 mm × 10 mm) and Pt cathode (10 mm × 20 mm) in an undivided cell with stirring. Then, the solution was diluted with sat. aq. $NaHCO_3$ and extracted with dichloromethane. The combined organic layers were dried over $Na_2SO_4$, filtered, and concentrated in vacuo. Silica gel column chromatography (*n*-hexane/EtOAc 10:1) gave the corresponding ring compounds.

*4,4-Dimethyl-2-(prop-1-en-2-yl)-1-tosylpyrrolidine (**2**) White solid.*

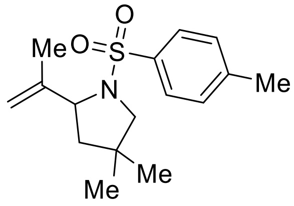


$^1$H NMR (400 MHz, $CDCl_3$) $\delta$ 7.70 (2H, d, $J$ = 8.2 Hz), 7.30 (2H, d, $J$ = 7.8 Hz), 4.96 (1H, s), 4.82 (1H, m), 4.06 (1H, t, $J$ = 8.7 Hz), 3.26 (1H, d, $J$ = 11.0 Hz), 3.19 (1H, d, $J$ = 11.0 Hz), 2.42 (3H, s), 1.72 (3H, s), 1.66-1.59 (2H, m), 1.03 (3H,s), 0.57 (3H,s); $^{13}$C{$^1$H} NMR (100 MHz, $CDCl_3$) $\delta$ 145.3, 143.2, 135.3, 129.5, 127.6, 112.0, 65.6, 62.0, 46.3, 37.4, 26.3, 25.9, 21.6, 17.0; HRMS $[M + H]^+$ calculated for $[C_{16}H_{24}NO_2S]^+$ 294.1522, found 294.1504.

The characterization data is compatible with the reported values.[1]

*4,4-Dimethyl-1-(phenylsulfonyl)-2-(prop-1-en-2-yl)pyrrolidine (**4**) Colorless oil.*

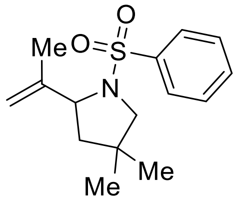


$^1$H NMR (600 MHz, $CDCl_3$) $\delta$ 7.83-7.82 (2H, m), 7.58 (1H, tt, $J$ = 6.9, 2.1 Hz), 7.52 (2H, t, $J$ = 7.6 Hz), 4.97 (1H, s), 4.83 (1H, m), 4.09 (1H, dd, $J$ = 9.6, 7.6 Hz), 3.28 (1H, dd, $J$ = 11.0, 1.4 Hz), 3.20 (1H, d, $J$ = 11.0 Hz), 1.71 (3H, s), 1.68-1.61 (2H, m), 1.03 (3H, s), 0.55 (3H, s); $^{13}C\{^1H\}$ NMR (100 MHz, $CDCl_3$) $\delta$ 145.2, 138.3, 132.6, 128.9, 127.5, 112.1, 65.7, 62.0, 46.3, 37.5, 26.2, 25.8, 17.0; HRMS $[M + H]^+$ calculated for $[C_{15}H_{22}NO_2S]^+$ 280.1366, found 280.1339.

The characterization data is compatible with the reported values.[1]

*1-((4-Methoxyphenyl)sulfonyl)-4,4-dimethyl-2-(prop-1-en-2-yl)pyrrolidine (**5**) White solid.*

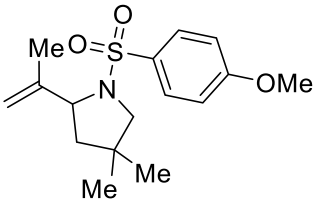


$^1$H NMR (400 MHz, $CDCl_3$) $\delta$ 7.77-7.34 (2H, m), 6.99-6.96 (2H, m), 4.96 (1H, s), 4.82 (1H, t, $J$ = 1.4 Hz), 4.04 (1H, t, $J$ = 8.7 Hz), 3.87 (3H, s), 3.22 (2H, q, $J$ = 10.5 Hz), 1.73 (3H, s), 1.69-1.60 (2H, m), 1.04 (3H,s), 0.60 (3H,s); $^{13}C\{^1H\}$ NMR (100 MHz, $CDCl_3$) $\delta$ 162.8, 145.4, 130.1, 129.6, 114.0, 111.9, 65.6, 62.0, 55.7, 46.4, 37.4, 26.3, 26.0, 17.0; HRMS $[M + H]^+$ calculated for $[C_{16}H_{24}NO_3S]^+$ 310.1471, found 310.1479.

The characterization data is compatible with the reported values.[1]

*4,4-Dimethyl-2-(prop-1-en-2-yl)-1-((4-(trifluoromethyl)phenyl)sulfonyl)pyrrolidine (**6**) White solid.*

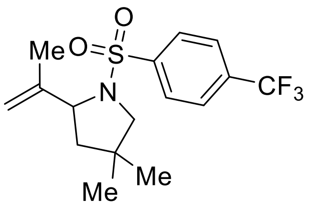

$^{1}$H NMR (400 MHz, $CDCl_3$) $\delta$ 7.95 (2H, d, $J$ = 8.2 Hz), 7.78 (2H, d, $J$ = 8.2 Hz), 4.95 (1H, s), 4.83 (1H, t, $J$ = 1.4 Hz), 4.16 (1H, dd, $J$ = 9.6, 7.3 Hz), 3.35 (1H, dd, $J$ = 10.5, 1.8 Hz), 3.19 (1H, d, $J$ = 11.0 Hz), 1.73 (1H, ddd, $J$ = 12.8, 7.8, 1.4 Hz), 1.67-1.61 (1H, m), 1.64 (3H, s), 1.06 (3H,s), 0.67 (3H,s); $^{13}C\{^1H\}$ NMR (100 MHz, $CDCl_3$) $\delta$ 144.5, 142.3, 134.2 (d, $J$ = 33.6 Hz), 127.9, 126.0 (q, $J$ = 3.8 Hz), 123.4 (d, $J$ = 272.2 Hz), 112.8, 65.8, 62.0, 46.1, 37.7, 25.9, 25.8, 16.9; HRMS $[M + H]^+$ calculated for $[C_{16}H_{21}F_3NO_2S]^+$ 348.1240, found 348.1225.

The characterization data is compatible with the reported values.[1]

*1-(Benzylsulfonyl)-4,4-dimethyl-2-(prop-1-en-2-yl)pyrrolidine (**8**) White solid.*

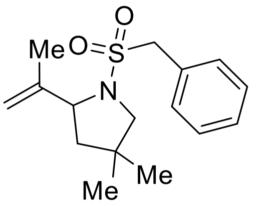

$^{1}$H NMR (600 MHz, $CDCl_3$) $\delta$ 7.44-7.33 (5H, m), 5.03 (1H, s), 4.88 (1H, t, $J$ = 2.1 Hz), 4.49 (1H, dd, $J$ = 9.6, 7.6 Hz), 4.23 (1H, d, $J$ = 13.1 Hz), 4.13 (1H, d, $J$ = 13.8 Hz), 3.30 (1H, dd, $J$ = 10.3, 2.1 Hz), 2.77 (1H, d, $J$ = 10.3 Hz), 1.82 (1H, ddd, $J$ = 12.4, 7.6, 1.4 Hz), 1.71 (3H, s), 1.57 (1H, dd, $J$ = 12.4, 9.6 Hz), 1.03 (3H,s), 1.02 (3H,s); $^{13}C\{^1H\}$ NMR (150 MHz, $CDCl_3$) $\delta$ 144.8, 130.9, 129.3, 128.8, 128.7, 128.6, 112.8, 65.3, 61.9, 59.4, 46.0, 38.5, 25.7, 25.7, 17.8; HRMS $[M + H]^+$ calculated for $[C_{16}H_{24}NO_2S]^+$ 294.1522, found 294.1552.

The characterization data is compatible with the reported values.[1]

*2-(Prop-1-en-2-yl)-1-tosylpyrrolidine (**10**) Colorless oil.*

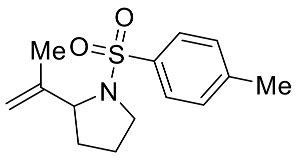

$^1$H NMR (400 MHz, CDCl$_3$) $\delta$ 7.72 (2H, d, $J$ = 8.2 Hz), 7.31 (2H, d, $J$ = 8.2 Hz), 5.00 (1H, s), 4.86 (1H, s), 4.04 (1H, t, $J$ = 6.4 Hz), 3.49-3.43 (1H, m), 3.28 (1H, dt, $J$ = 10.1, 7.3 Hz), 2.43 (3H, s), 1.85-1.76 (1H, m), 1.74 (3H, s), 1.72-1.66 (2H, m), 1.59-1.50 (1H, m); $^{13}$C{$^1$H} NMR (100 MHz, CDCl$_3$) $\delta$ 145.1, 143.3, 135.0, 129.6, 127.6, 111.8, 65.0, 49.3, 31.5, 24.1, 21.6, 18.7; HRMS [M + H]$^+$ calculated for [$C_{14}H_{20}NO_2S$]$^+$ 266.1209, found 266.1217.

The characterization data is compatible with the reported values.[1]

*3,3,5,6-Tetramethyl-1-tosyl-1,2,3,6-tetrahydropyridine and 2,5,5-trimethyl-3-methylene-1-tosylpiperidine (**12**, 2:1 r.r.) White solid.*

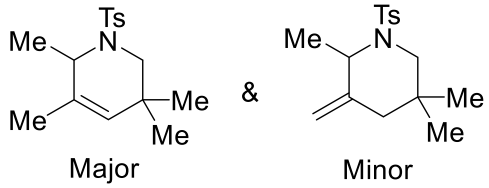

*Isolated as a 2:1 mixture of regioisomers.*

Major: $^1$H NMR (600 MHz, CDCl$_3$) $\delta$ 7.72 (2H, d, $J$ = 8.3 Hz), 7.28-7.26 (2H, m), 5.14 (1H, s), 4.18 (1H, q, $J$ = 6.9 Hz), 3.42 (1H, d, $J$ = 12.4 Hz), 2.78 (1H, d, $J$ = 13.1 Hz), 2.41 (3H, s), 1.63 (3H, s), 1.04 (3H, d, $J$ = 6.9 Hz), 0.93 (3H, s), 0.91 (3H, s); $^{13}$C{$^1$H} NMR (150 MHz, CDCl$_3$) $\delta$ 142.9, 138.8, 132.8, 131.1, 129.6, 127.2, 52.6, 49.7, 33.3, 27.0, 26.8, 21.6, 20.8, 16.3.

Minor: $^{1}$H NMR (600 MHz, $CDCl_3$) $\delta$ 7.69 (2H, d, $J$ = 8.3 Hz), 7.28-7.26 (2H, m), 4.87 (1H, s), 4.65 (1H, t, $J$ = 2.1 Hz), 4.62 (1H, q, $J$ = 6.9 Hz), 3.27 (1H, dd, $J$ = 14.4, 1.4 Hz), 2.83 (1H, d, $J$ = 13.1 Hz), 2.41 (3H, s), 2.14 (1H, d, $J$ = 13.8 Hz), 1.81 (1H, dd, $J$ = 15.8, 2.1 Hz), 1.09 (3H, d, $J$ = 6.9 Hz), 0.94 (3H, s), 0.74 (3H, s); $^{13}C\{^{1}H\}$ NMR (150 MHz, $CDCl_3$) $\delta$ 144.2, 142.9, 138.4, 129.6, 127.1, 110.9, 55.2, 50.6, 42.2, 32.7, 28.0, 23.5, 21.6, 16.5.

HRMS $[M + H]^+$ calculated for $[C_{16}H_{24}NO_2S]^+$ 294.1522, found 294.1526.

*2,4,4-Trimethyl-2-(prop-1-en-2-yl)-1-tosylpyrrolidine (**15**) White solid.*

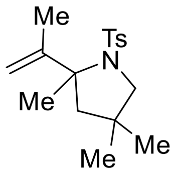


$^{1}$H NMR (400 MHz, $CDCl_3$) $\delta$ 7.74 (2H, d, $J$ = 8.2 Hz), 7.27 (2H, d, $J$ = 8.2 Hz), 5.09 (1H, s), 4.87 (1H, s), 3.23 (1H, d, $J$ = 10.1 Hz), 3.18 (1H, d, $J$ = 9.6 Hz), 2.42 (3H, s), 1.97 (1H, d, $J$ = 13.3 Hz), 1.79 (3H, s), 1.65 (3H, s), 1.59 (1H, d, $J$ = 13.3 Hz), 1.06 (3H, s), 0.94 (3H, s); $^{13}C\{^{1}H\}$ NMR (150 MHz, $CDCl_3$) $\delta$ 149.7, 142.8, 138.3, 129.3, 127.4, 110.9, 71.3, 62.4, 54.9, 36.5, 28.3, 27.7, 25.7, 21.6, 19.8; HRMS $[M + H]^+$ calculated for $[C_{17}H_{26}NO_2S]^+$ 308.1679, found 308.1656.

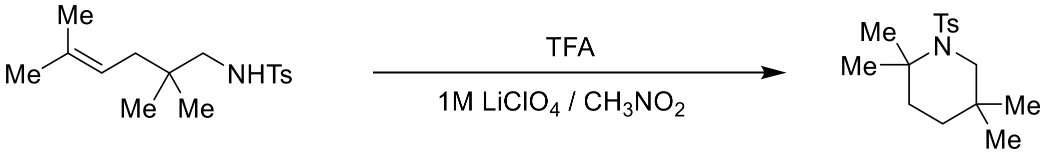

*General procedure for non-electrochemical cyclization.* The appropriate alkene (0.20 mmol) and TFA (0.20 mmol) were added to a solution of $LiClO_4/CH_3NO_2$ (1.0 M, 4 mL) and the resulting reaction mixture was stirred at rt for 18 h. The solution was diluted with sat. $NaHCO_3$ aq. and extracted with EtOAc. The combined organic layers were dried over $Na_2SO_4$, filtered, and concentrated in vacuo. Silica gel column chromatography (*n*-hexane/EtOAc 10:1) gave the corresponding ring compounds.

*2,2,5,5-Tetramethyl-1-tosylpiperidine (**3**) White solid.*

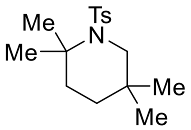

$^1$H NMR (400 MHz, $CDCl_3$) $\delta$ 7.67 (2H, d, $J$ = 8.7 Hz), 7.24 (2H, d, $J$ = 8.2 Hz), 3.14 (2H, s), 2.39 (3H, s), 1.47-1.44 (2H, m), 1.33-1.30 (2H, m), 1.24 (6H, s), 0.94 (6H, s); $^{13}$C{$^1$H} NMR (100 MHz, $CDCl_3$) $\delta$ 142.7, 140.3, 129.4, 127.3, 57.6, 54.2, 38.0, 33.8, 31.1, 26.3, 25.8, 21.6; HRMS [M + H]$^+$ calculated for $[C_{16}H_{16}NO_2S]^+$ 296.1679, found 296.1683.

*2-Ethyl-2,4,4-trimethyl-1-tosylpyrrolidine (**13**) White solid.*

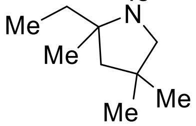

$^1$H NMR (400 MHz, $CDCl_3$) $\delta$ 7.74 (2H, d, $J$ = 8.7 Hz), 7.27 (2H, d, $J$ = 8.2 Hz), 3.09 (1H, d, $J$ = 9.6 Hz), 3.04 (1H, d, $J$ = 9.6 Hz), 2.41 (3H, s), 1.97 (1H, sext, $J$ = 6.0 Hz), 1.87-1.78 (2H, m), 1.49 (1H, d, $J$ = 12.4

Hz), 1.48 (3H, s), 1.01 (3H, s), 0.97 (3H, s), 0.89 (3H, t, $J$ = 7.3 Hz); $^{13}C\{^1H\}$ NMR (100 MHz, $CDCl_3$) $\delta$ 142.6, 138.7, 129.3, 127.3, 69.7, 61.7, 52.5, 36.2, 34.8, 27.7, 27.5, 27.1, 21.5, 9.5; HRMS $[M + H]^+$ calculated for $[C_{16}H_{26}NO_2S]^+$ 296.1679, found 296.1665.

*2-Isopropyl-2,4,4-trimethyl-1-tosylpyrrolidine (**16**), 2,2,3,5,5-pentamethyl-1-tosylpiperidine (**17**) White solid.*

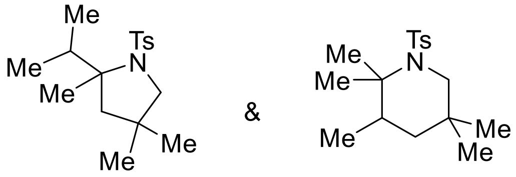


**16** **17**

*Isolated as a 1:1 mixture of **16** & **17**.*

(**16**): $^1H$ NMR (400 MHz, $CDCl_3$) $\delta$ 7.72 (2H, d, $J$ = 8.2 Hz), 7.24-7.22 (2H, m), 3.10 (1H, d, $J$ = 10.1 Hz), 2.98 (1H, d, $J$ = 10.1 Hz), 2.52 (1H, sept, $J$ = 6.9 Hz), 2.38 (3H, s), 1.85 (1H, d, $J$ = 13.3 Hz), 1.53 (3H, s), 1.27 (1H, d, $J$ = 13.3 Hz), 0.97 (3H, s), 0.90 (3H, d, $J$ = 6.9 Hz), 0.84 (3H, s), 0.79 (3H, d, $J$ = 6.9 Hz); $^{13}C\{^1H\}$ NMR (100 MHz, $CDCl_3$) $\delta$ 142.5, 138.9, 129.3, 127.2, 73.1, 61.8, 47.6, 38.0, 36.0, 27.9, 27.0, 24.5, 18.6, 17.1.

(**17**): $^1H$ NMR (400 MHz, $CDCl_3$) $\delta$ 7.66 (2H, d, $J$ = 8.7 Hz), 7.24-7.22 (2H, m), 3.66 (1H, dd, $J$ = 13.3, 1.4 Hz), 2.82 (1H, d, $J$ = 12.8 Hz), 2.38 (3H, s), 1.78 (1H, sext, $J$ = 6.4 Hz), 1.39 (3H, s), 1.23-1.13 (2H, m), 1.03 (3H, s), 0.95 (3H, s), 0.87 (3H, s), 0.73 (3H, d, $J$ = 6.9 Hz); $^{13}C\{^1H\}$ NMR (100 MHz, $CDCl_3$) $\delta$ 142.5, 141.1, 129.4, 127.1, 62.6, 53.9, 43.3, 37.8, 31.2, 29.0, 27.4, 26.7, 21.5, 17.8, 16.1.

HRMS $[M + H]^+$ calculated for $[C_{17}H_{28}NO_2S]^+$ 310.1835, found 310.1837.

## References

[1] Reed, N. L.; Lutovsky, G. A.; Yoon, T. P. *J. Am. Chem. Soc.* **2021**, *143*, 6065–6070.

[2] Venning, A. R. O.; Kwiatkowski, M. R.; Roque Peña, J. E.; Lainhart, B. C.; Guruparan, A. A.; Alexanian, E. J. *J. Am. Chem. Soc.* **2017**, *139*, 11595–11600.

[3] Kou, X.; Shao, Q.; Ye, C.; Yang, G.; Zhang, W. *J. Am. Chem. Soc.* **2018**, *140*, 7587–7597.

### 3. Cyclic Voltammetry Studies

Cyclic voltammetry experiments were performed in 1,2-DCE (4.5 mL), $CH_3CN$ (0.5 mL) with analyte (2 mM) and $Bu_4NOTf$ (0.1 M) using a glassy carbon working electrode, platinum wire electrode, Ag/AgCl KCl aq. reference electrode, and a scan rate of 50 mV/s.

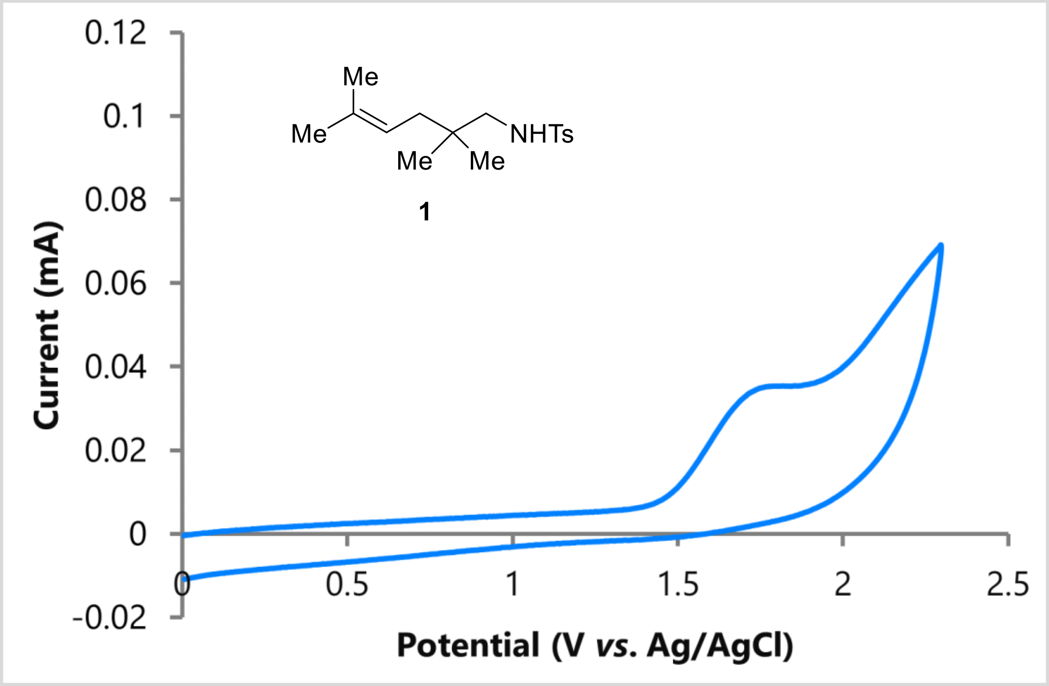


**Figure S1.** Cyclic voltammogram of alkene **1**, $E_P$ = 1.77 V.

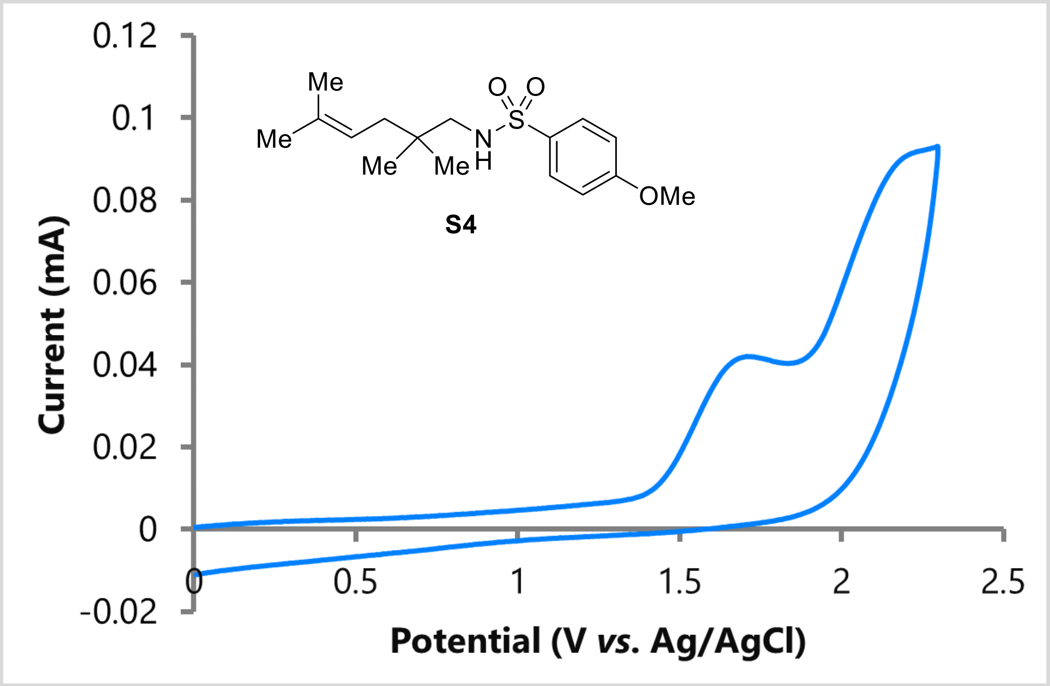


**Figure S2.** Cyclic voltammogram of alkene **S4**, $E_P$ = 1.70 V.

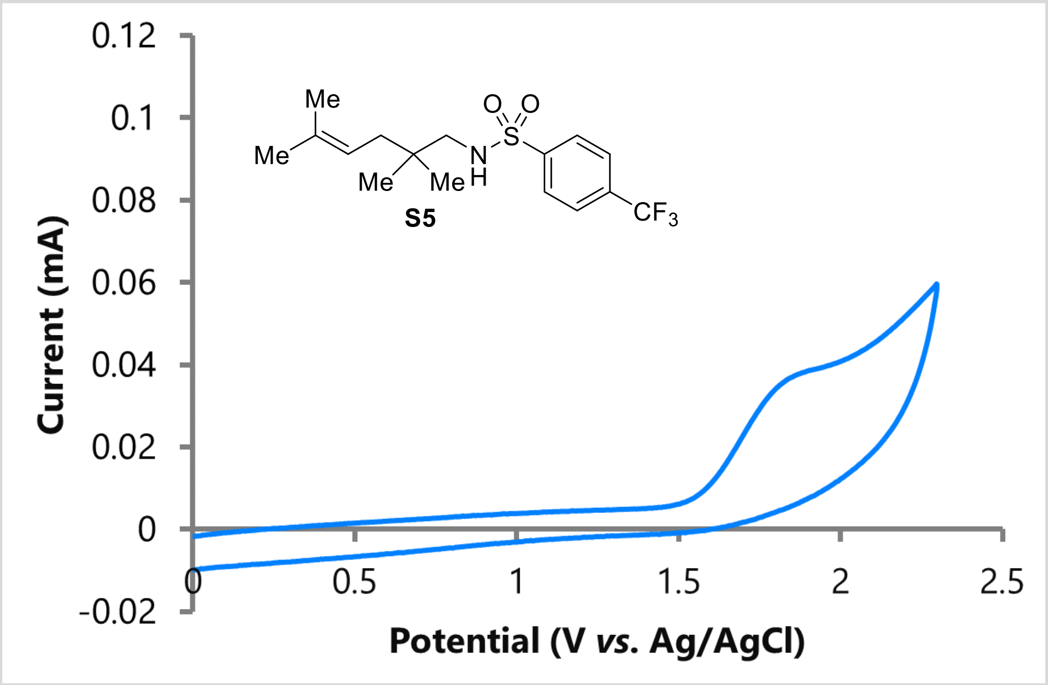


**Figure S3.** Cyclic voltammogram of alkene **S5**, $E_P$ = 1.86 V.

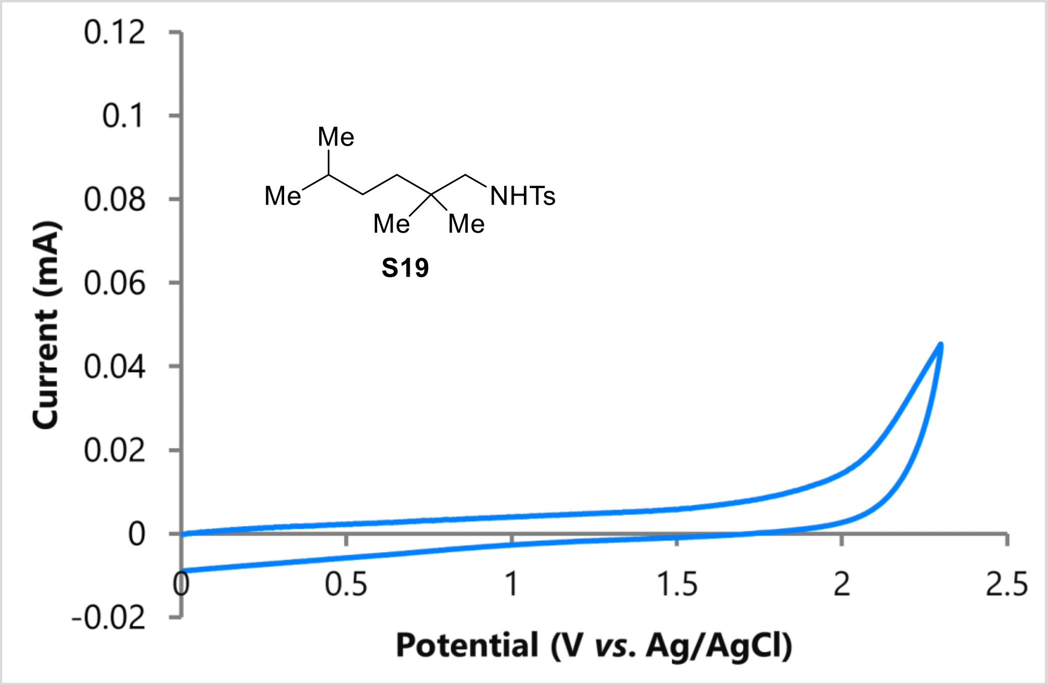


**Figure S4.** Cyclic voltammogram of the aryl sulfonamide without trisubstituted alkene **S19**, $E_P$ > 2.3 V.

## 4. Copies of 1H and 13C NMR spectra

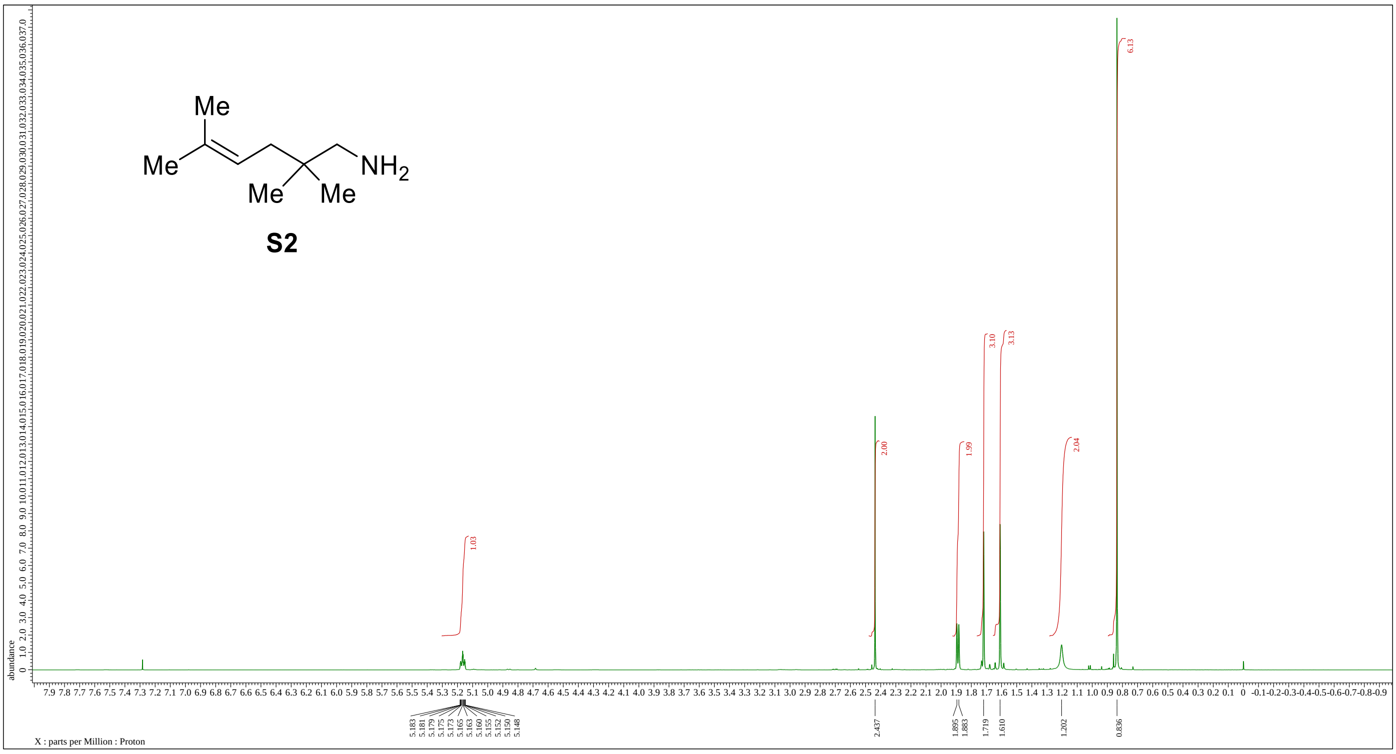


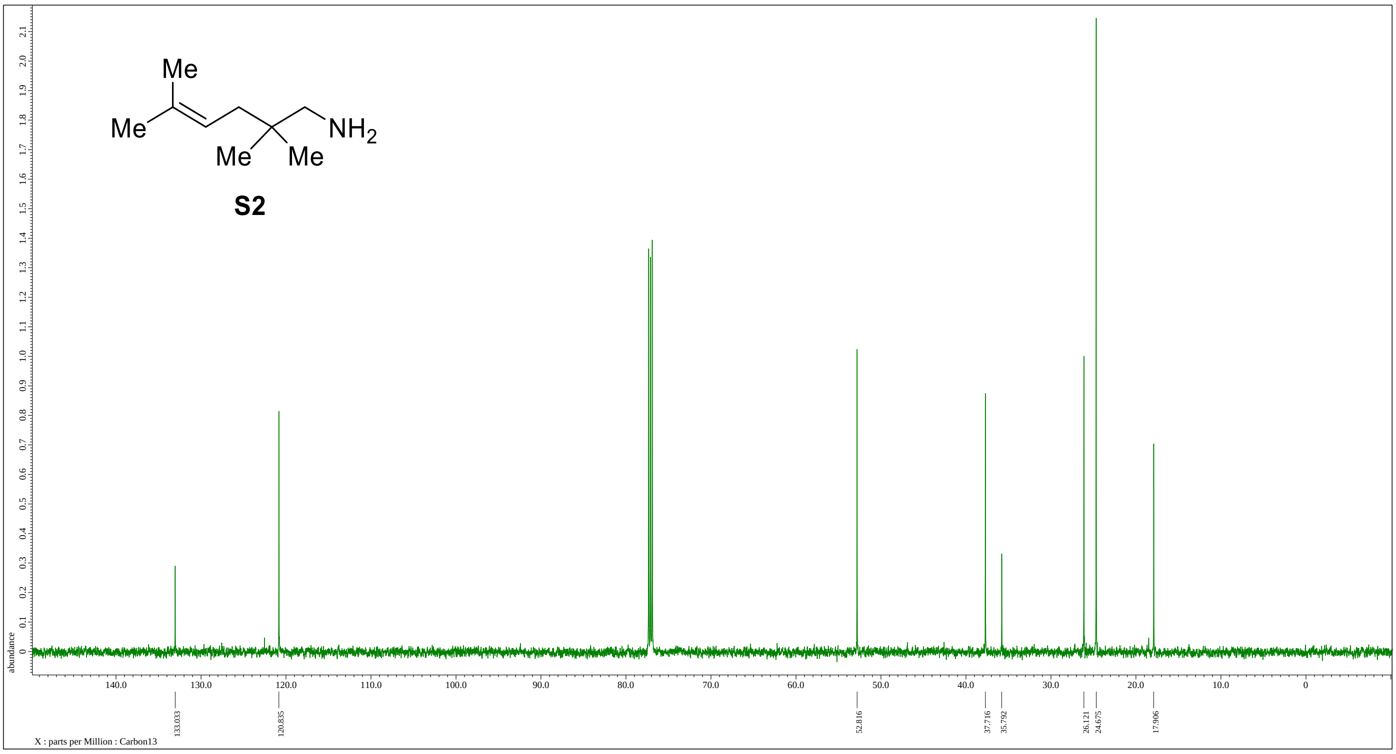

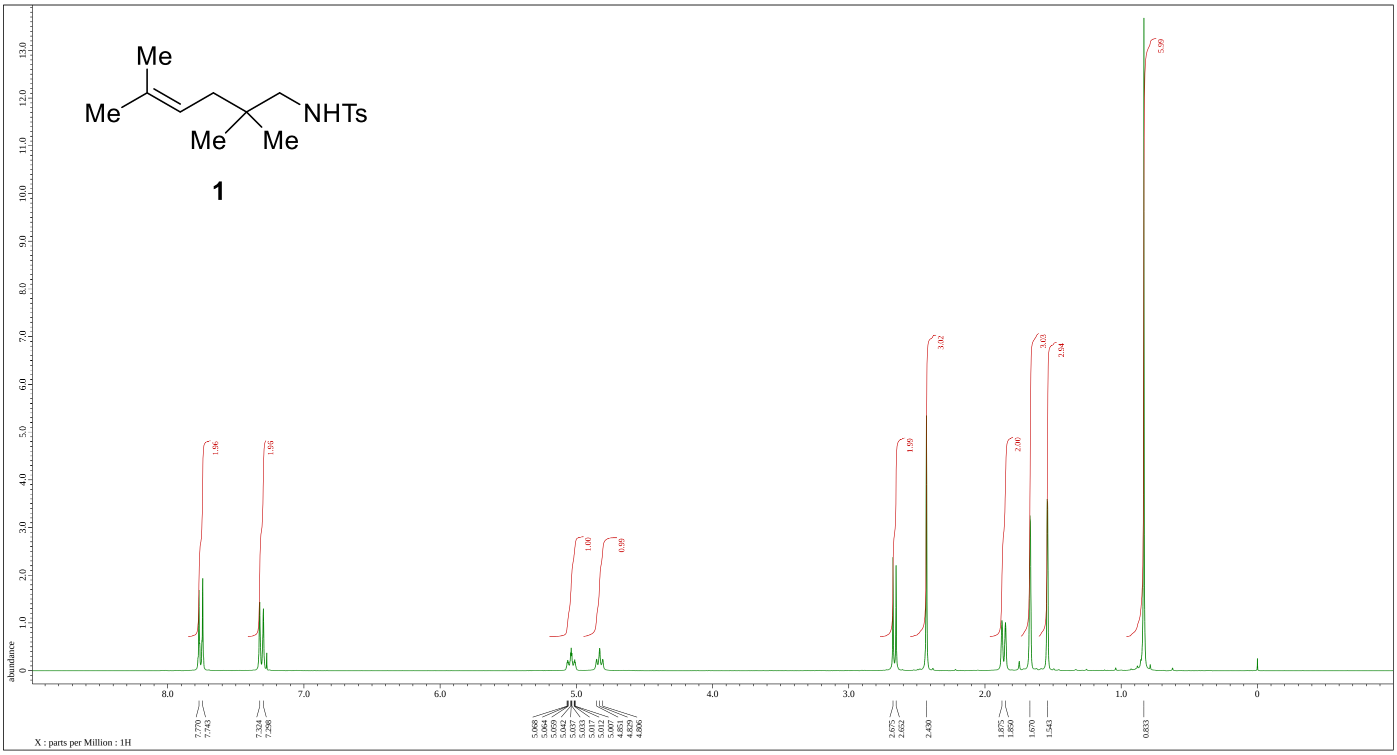
Me
Me
NHTs
Me
Me
1
abundance
X : parts per Million : 1H
1.96
1.96
1.00
0.99
1.99
3.02
2.00
3.03
2.94
5.99
7.770
7.743
7.324
7.298
5.068
5.064
5.059
5.042
5.037
5.033
5.017
5.012
5.007
4.851
4.829
4.806
2.675
2.652
2.430
1.875
1.850
1.670
1.543
0.833

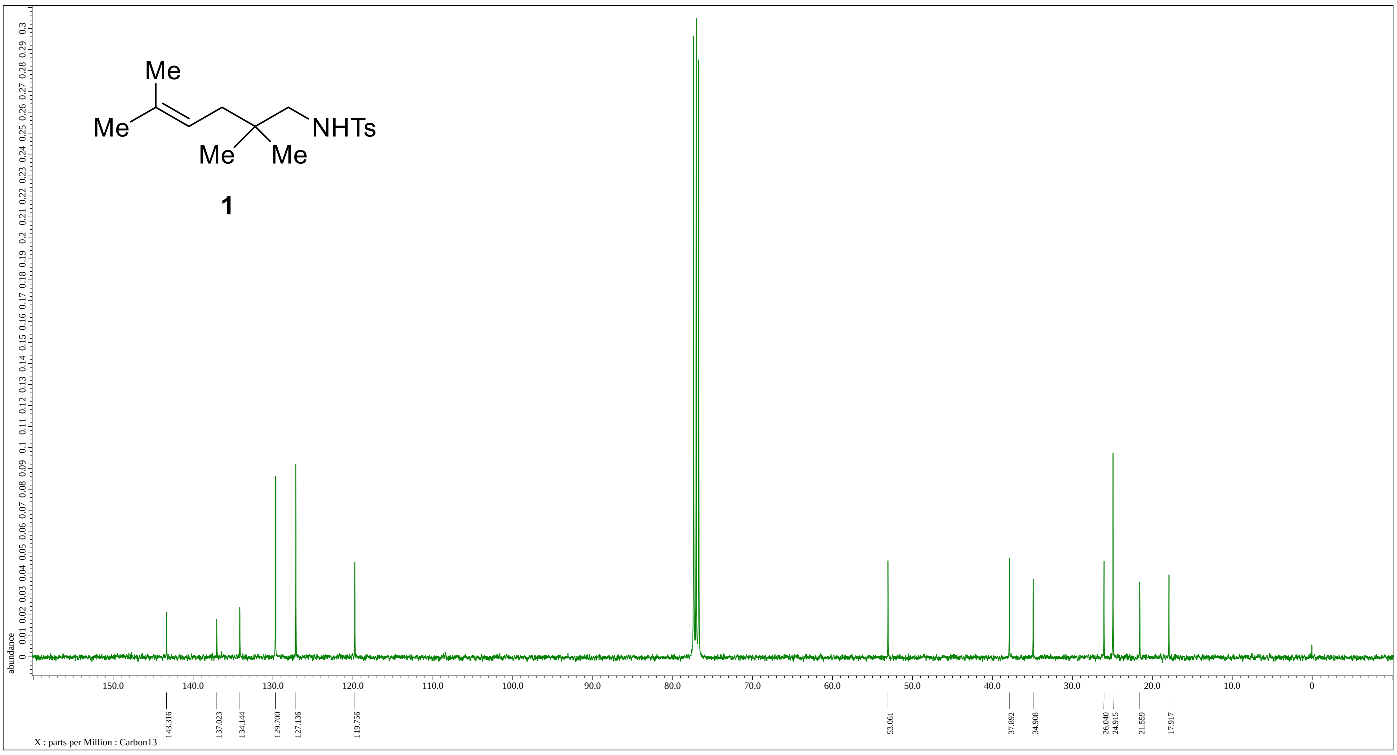
Me
Me
NHTs
Me
Me
1
abundance
X : parts per Million : Carbon13
143.316
137.023
134.144
129.700
127.136
119.756
53.061
37.892
34.908
26.040
24.915
21.559
17.917

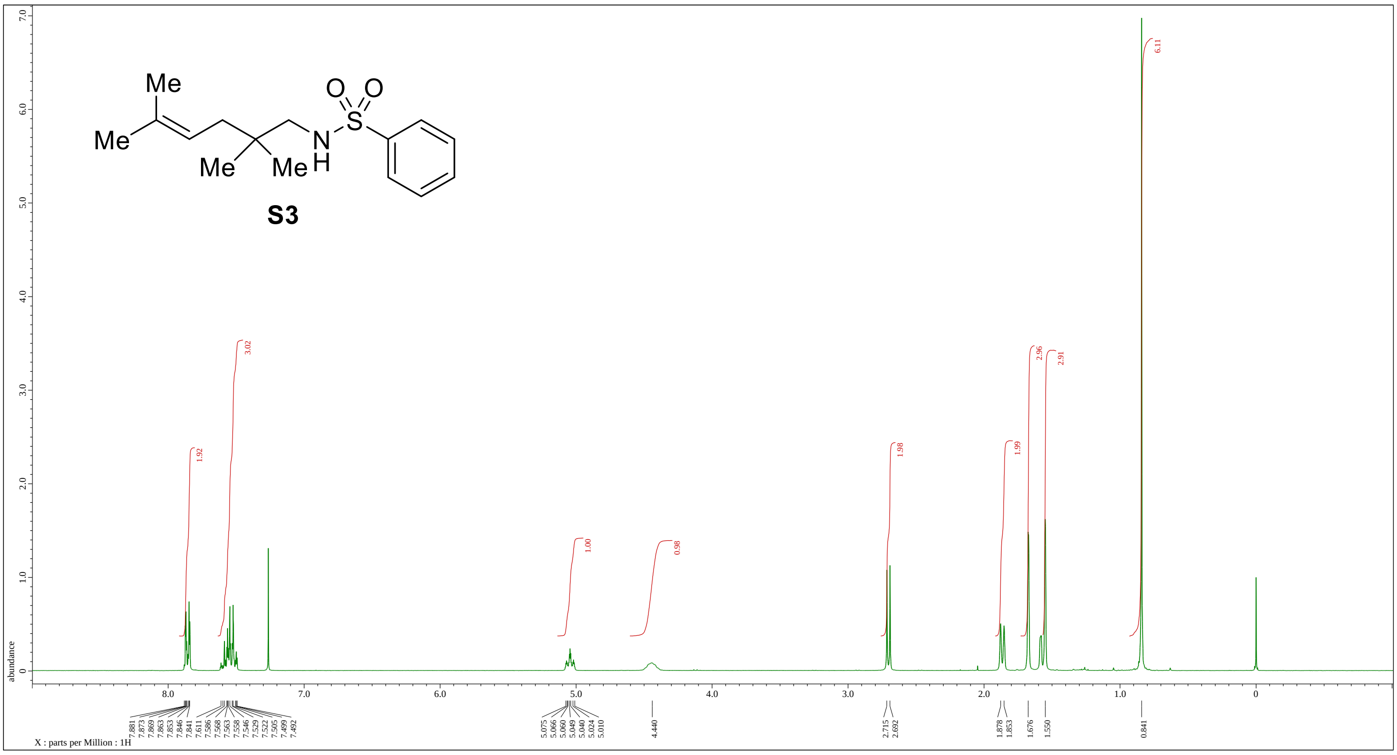
Me
Me
Me
Me
N
H
S
O
O
S3
abundance
X : parts per Million : 1H

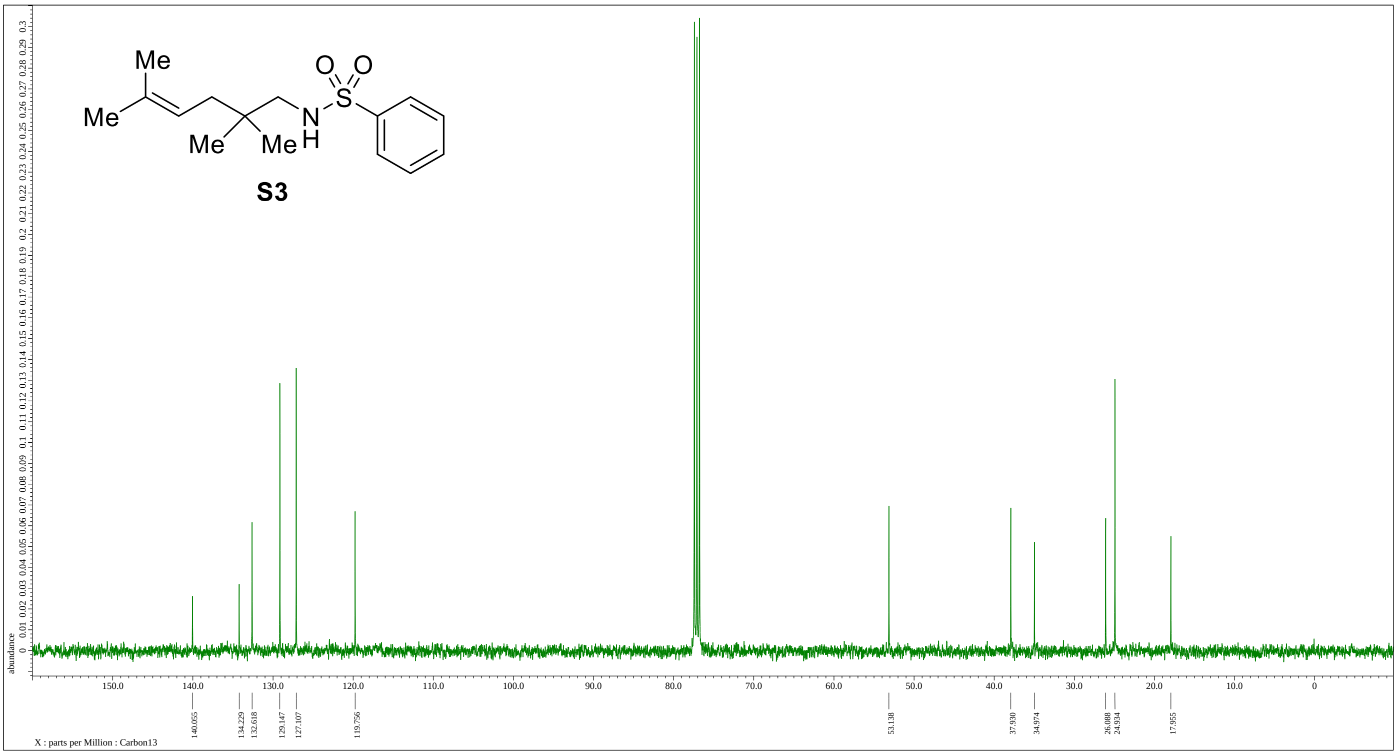
Me
Me
Me
Me
N
H
S
O
O
S3
abundance
X : parts per Million : Carbon13

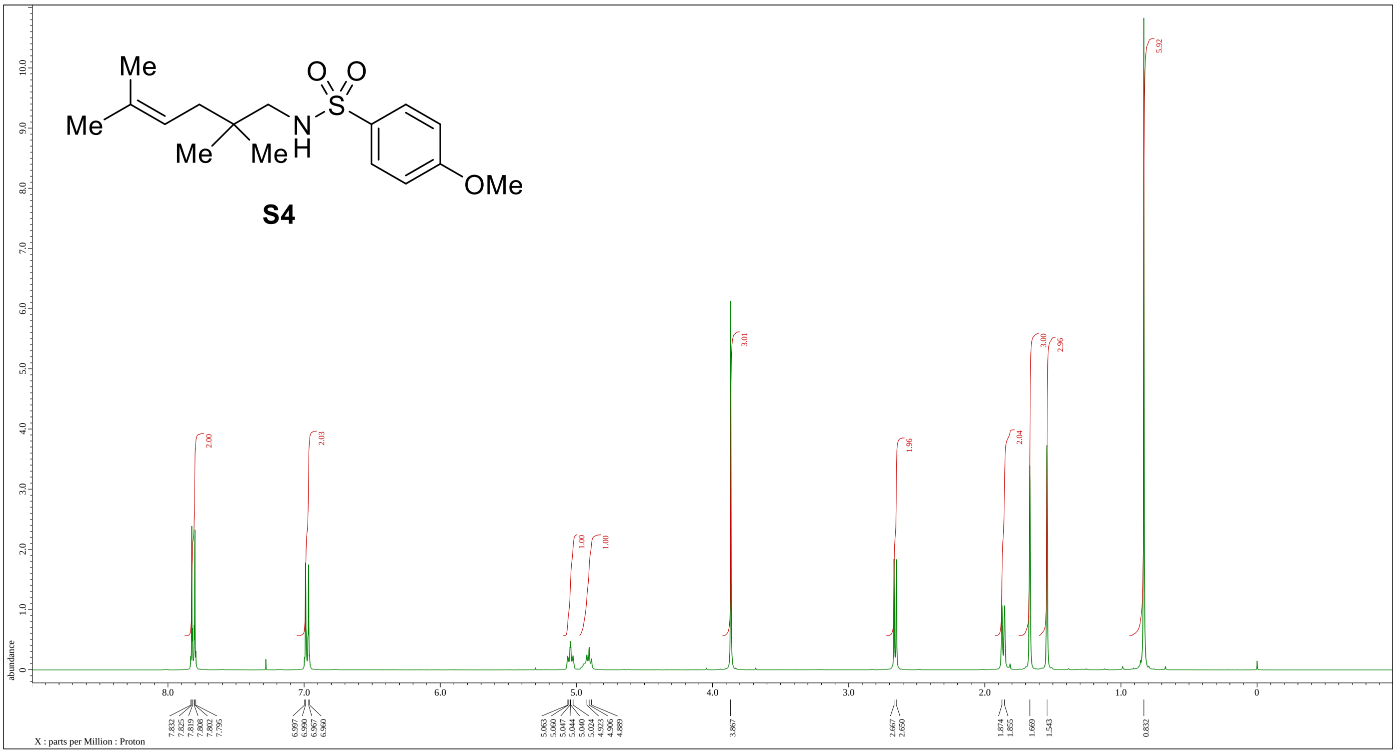
Me
Me
Me
Me
O
O
S
N
H
OMe
S4
2.00
2.03
1.00
1.00
3.01
1.96
2.04
3.00
2.96
5.92
abundance
7.832
7.825
7.819
7.808
7.802
7.795
6.997
6.990
6.967
6.960
5.063
5.060
5.047
5.044
5.040
5.024
4.923
4.906
4.889
3.867
2.667
2.650
1.874
1.855
1.669
1.543
0.832
X : parts per Million : Proton

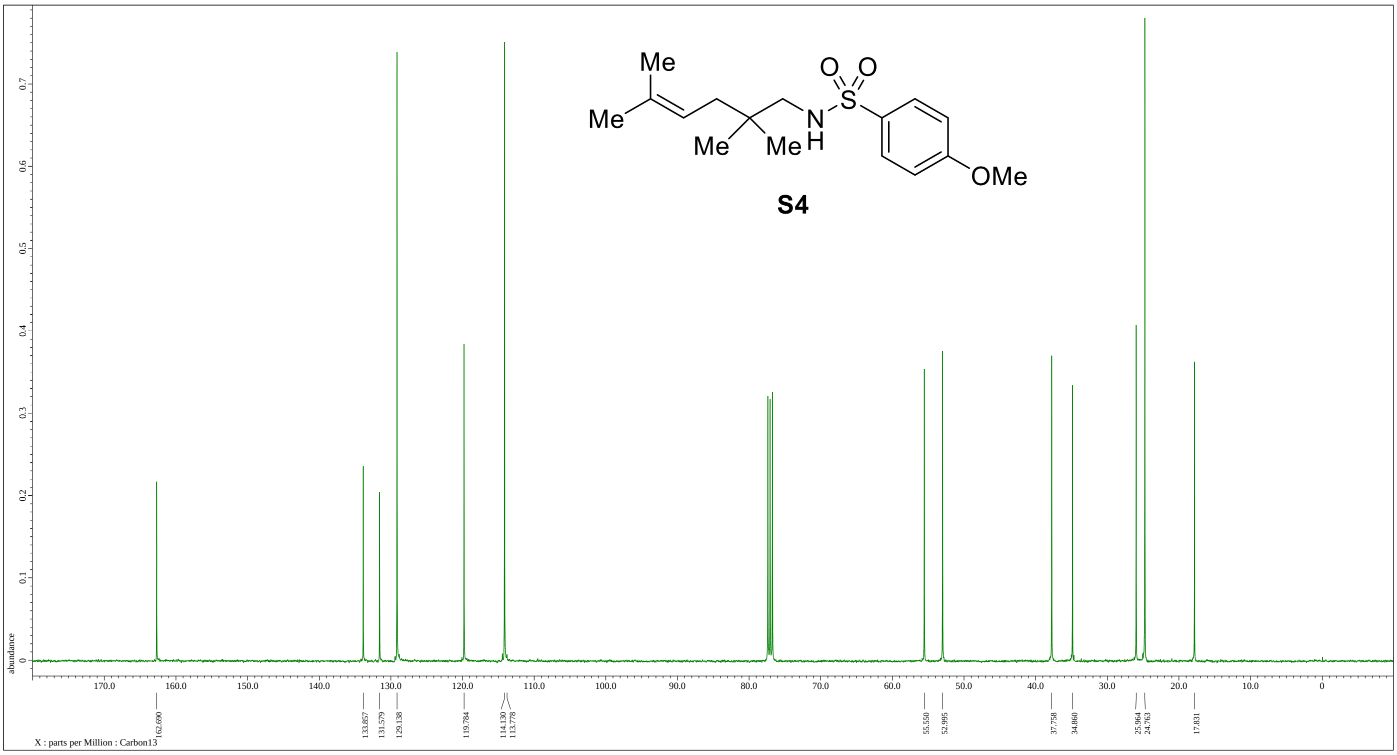
Me
Me
Me
Me
O
O
S
N
H
OMe
S4
abundance
162.690
133.857
131.579
129.138
119.784
114.130
113.778
55.550
52.995
37.758
34.860
25.964
24.763
17.831
X : parts per Million : Carbon13

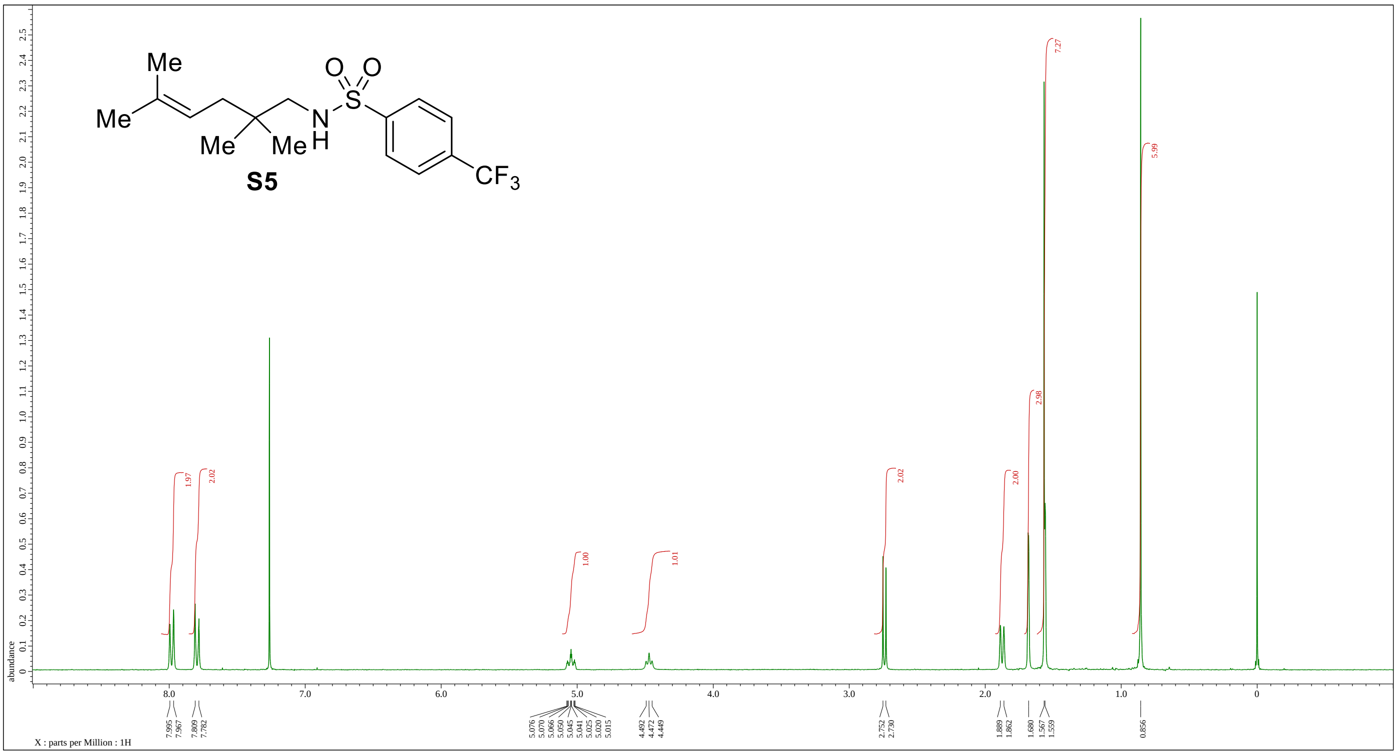

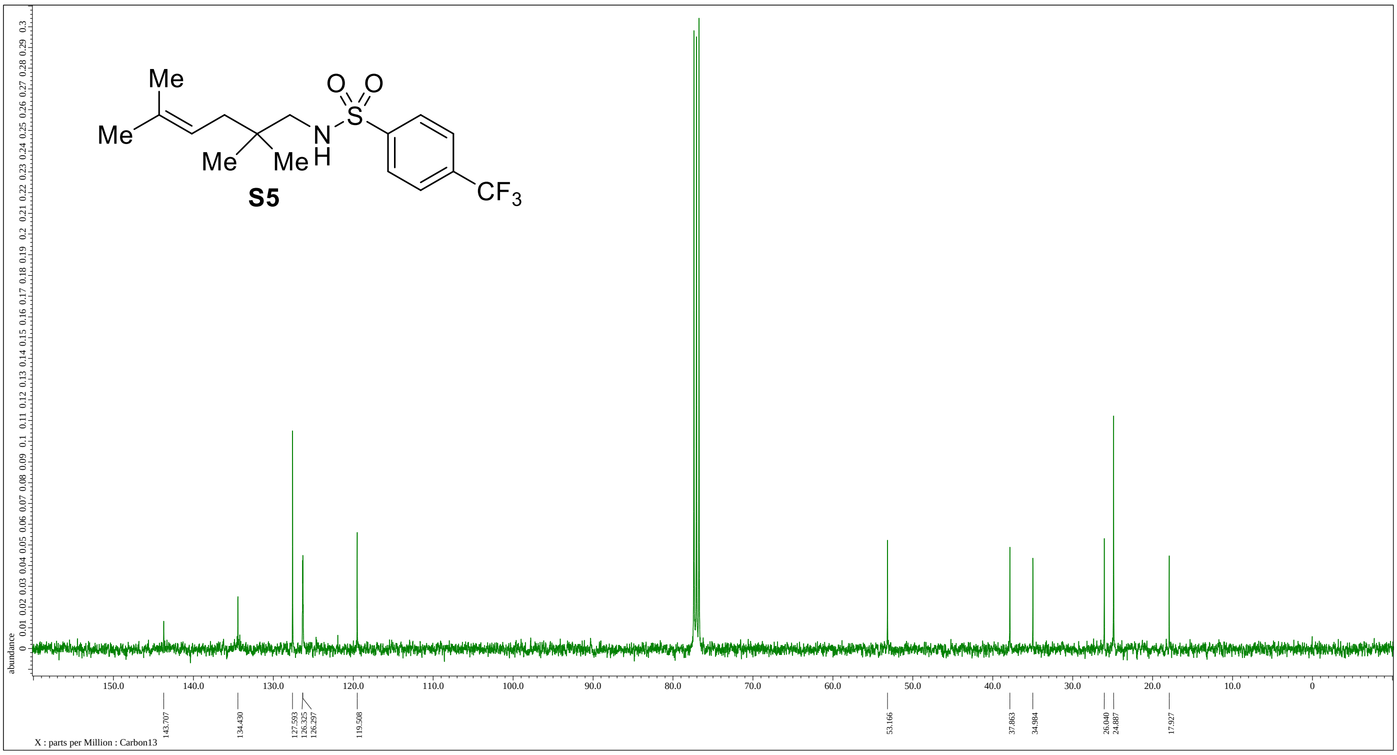

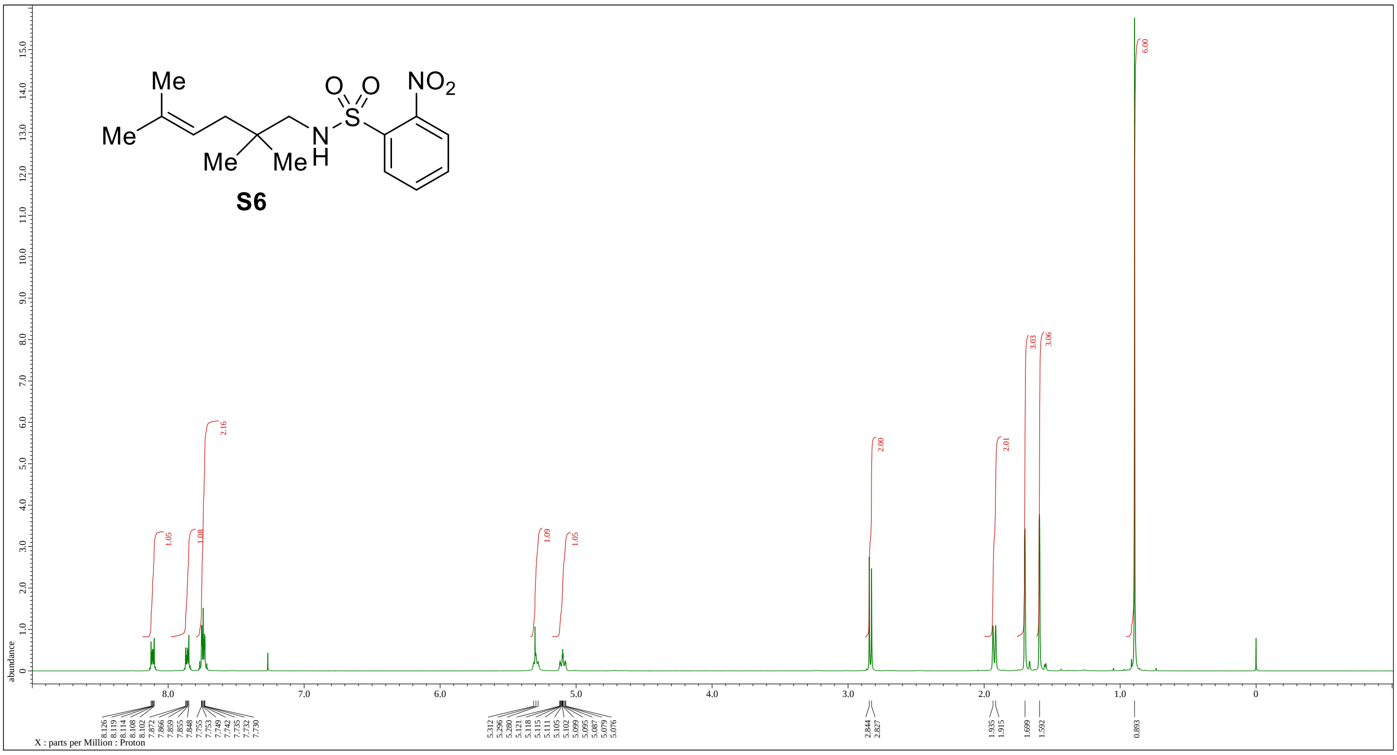
Me
Me
Me
Me
H
N
S
O O
NO2
S6
abundance
1.05
1.08
2.16
1.09
1.05
2.00
2.01
3.03
3.06
6.00
8.126 8.119 8.114 8.108 8.102 7.872 7.866 7.859 7.855 7.848 7.755 7.753 7.749 7.742 7.735 7.732 7.730
5.312 5.296 5.280 5.121 5.118 5.115 5.111 5.105 5.102 5.099 5.095 5.087 5.079 5.076
2.844 2.827
1.935 1.915
1.699
1.592
0.893
X : parts per Million : Proton

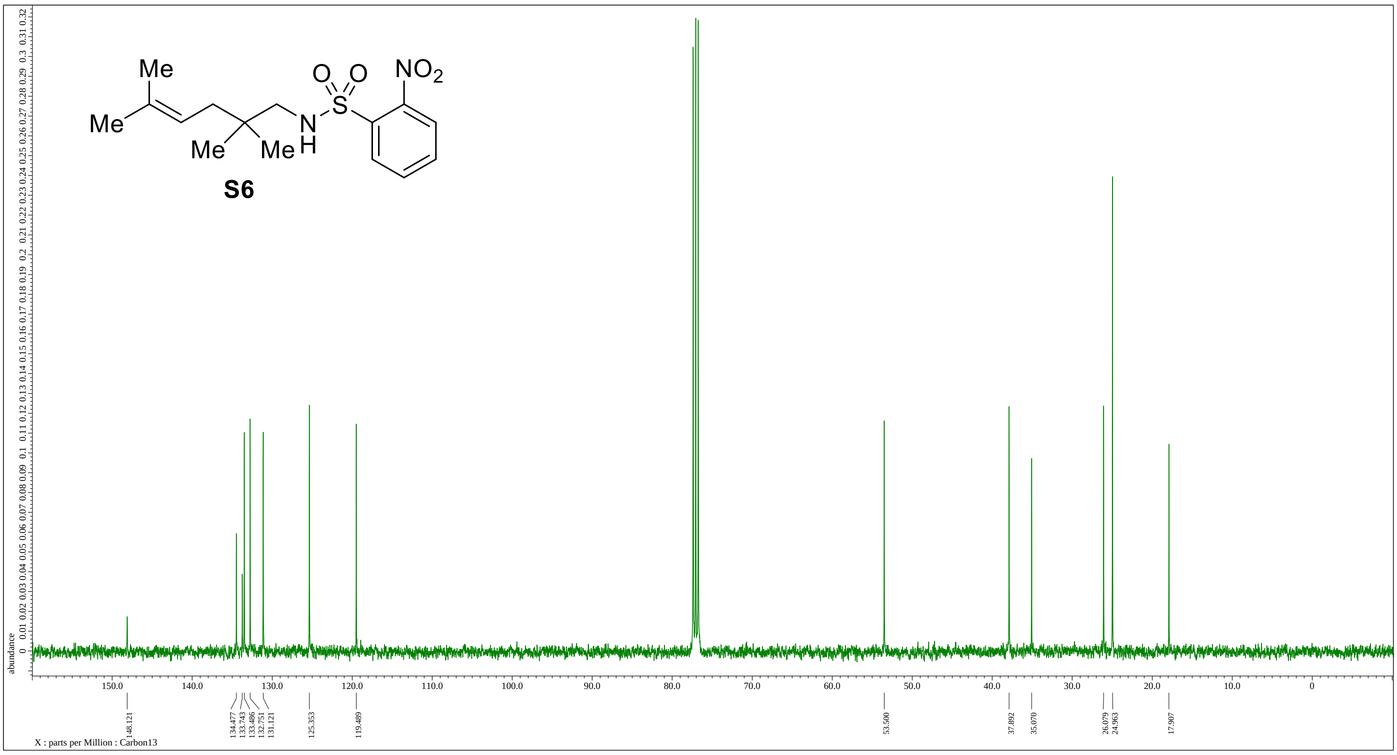
Me
Me
Me
Me
H
N
S
O O
NO2
S6
abundance
148.121
134.477 133.743 133.486 132.751 131.121
125.353
119.489
53.500
37.892
35.070
26.079 24.963
17.907
X : parts per Million : Carbon13

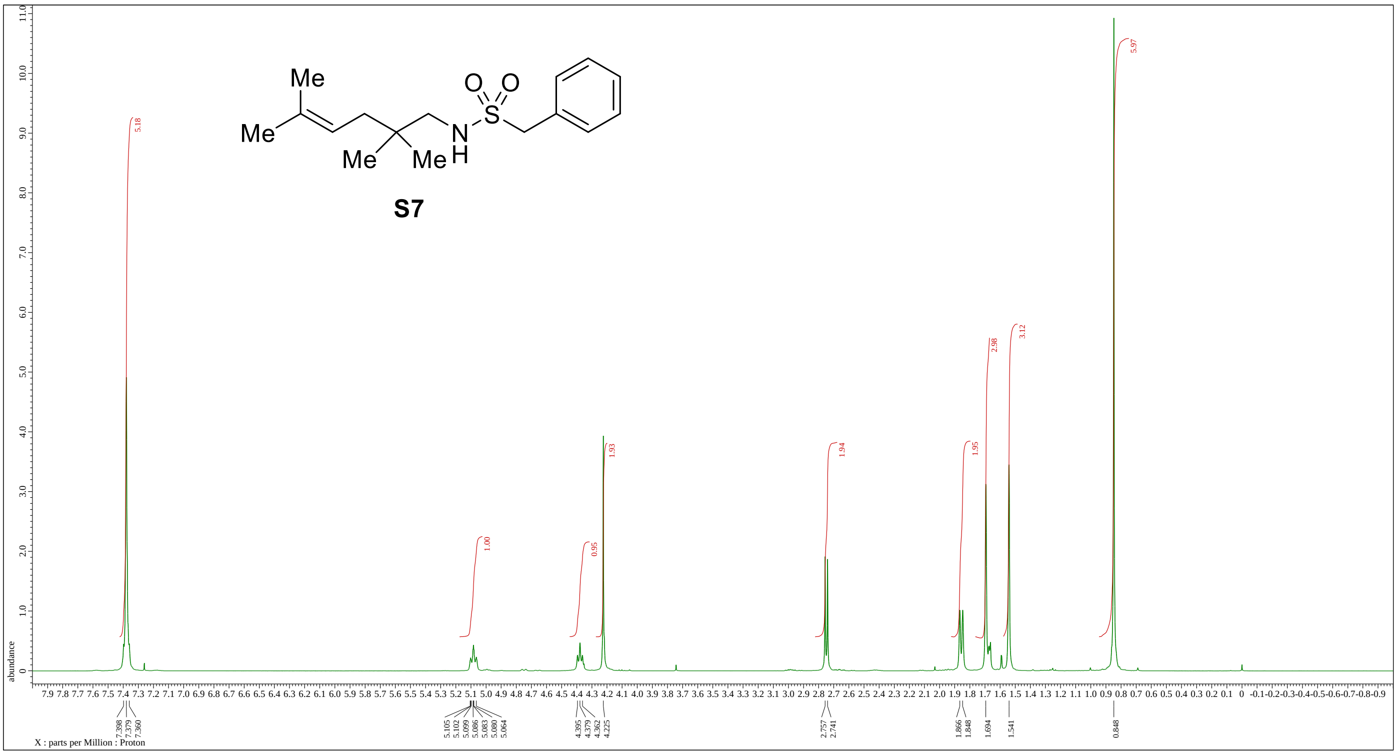

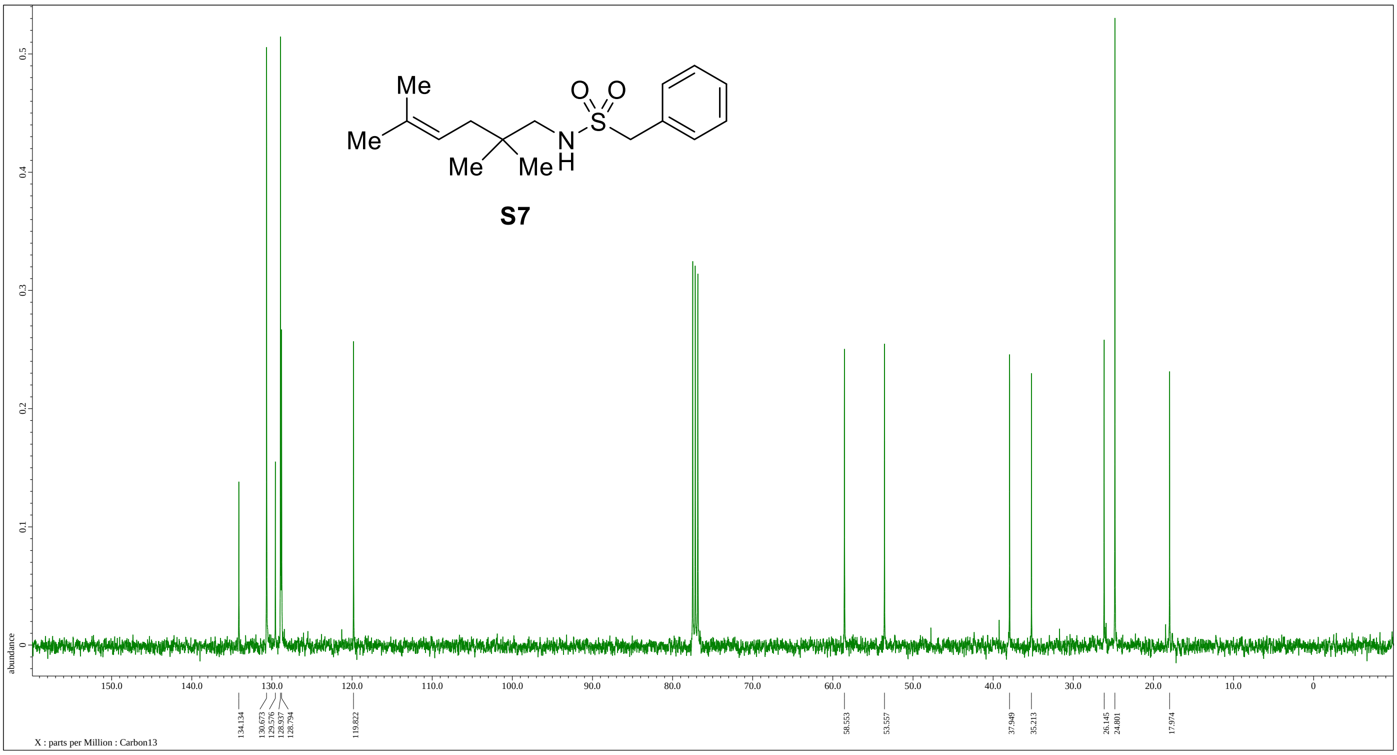

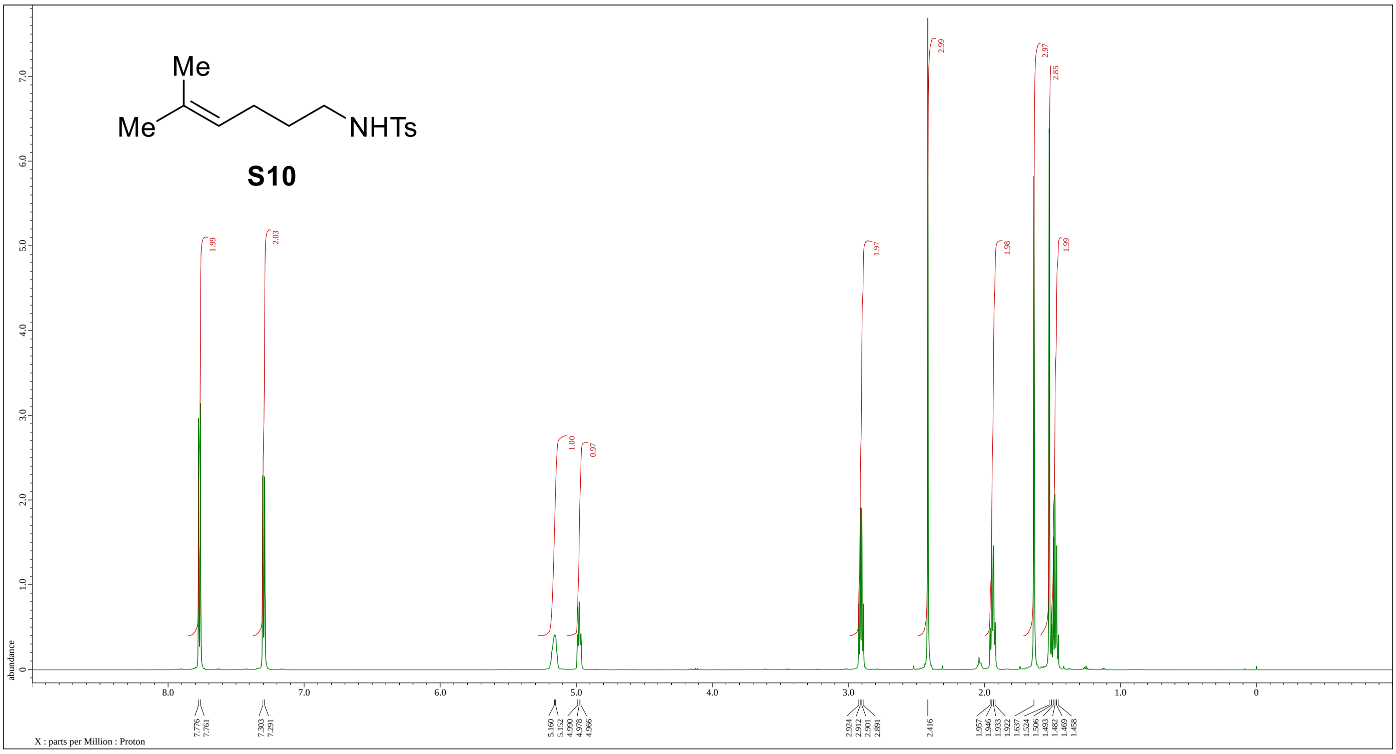

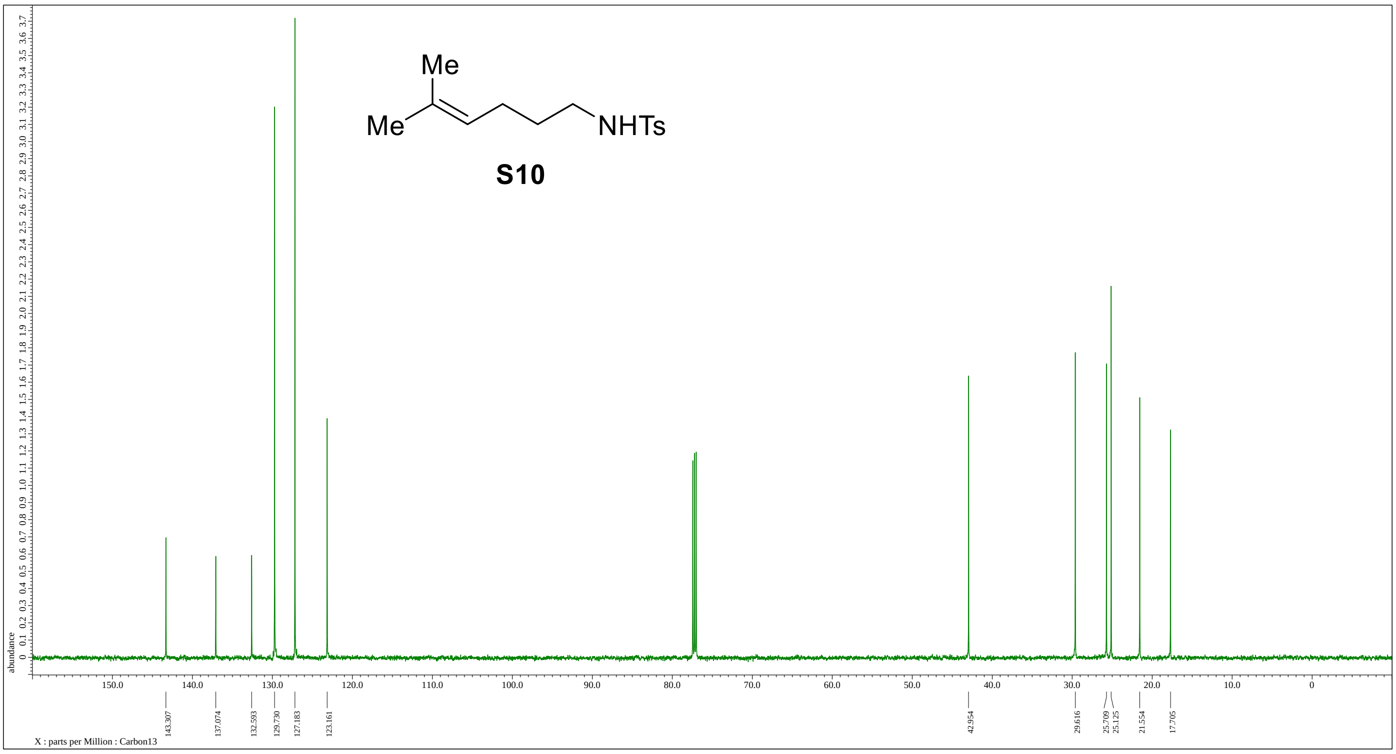

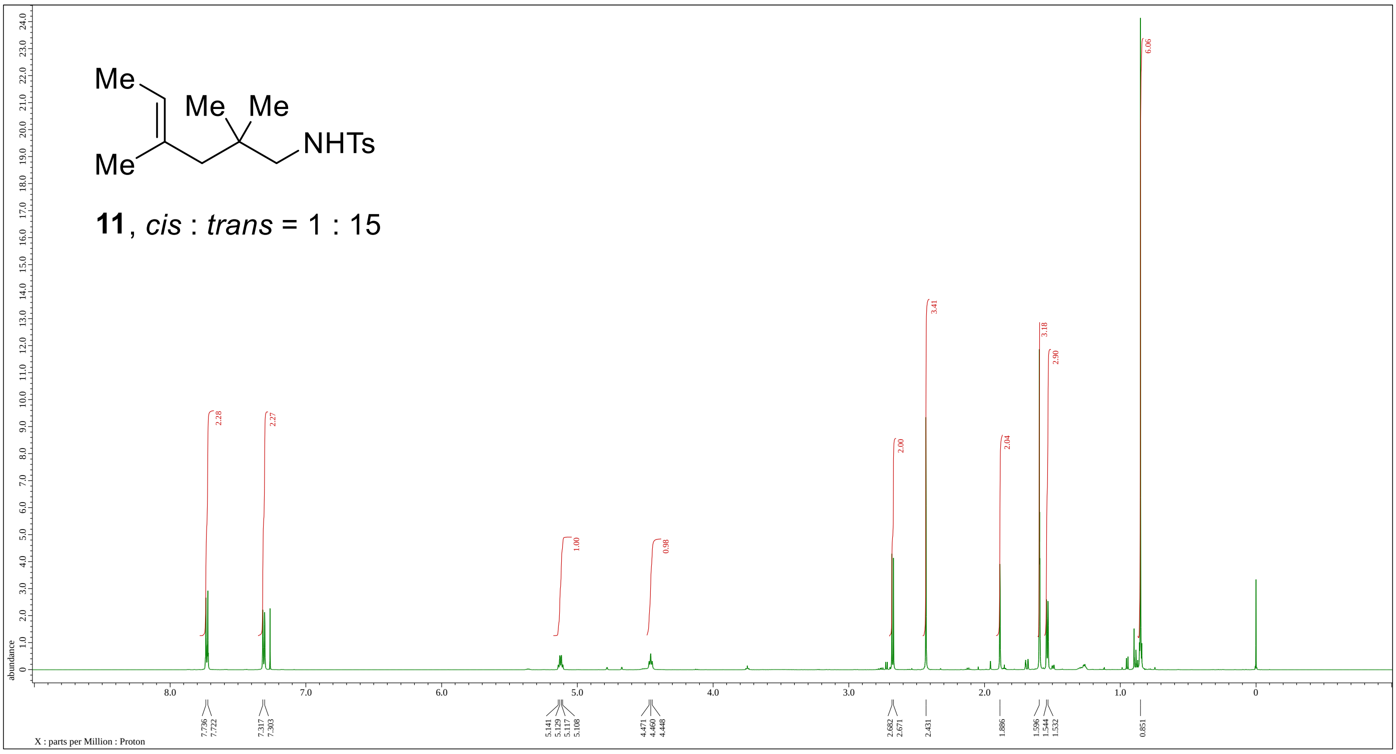
Me
Me Me
NHTs
Me
11, cis : trans = 1 : 15
abundance
X : parts per Million : Proton
7.736
7.722
7.317
7.303
5.141
5.129
5.117
5.108
4.471
4.460
4.448
2.682
2.671
2.431
1.886
1.596
1.544
1.532
0.851
2.28
2.27
1.00
0.98
2.00
3.41
2.04
3.18
2.90
6.06

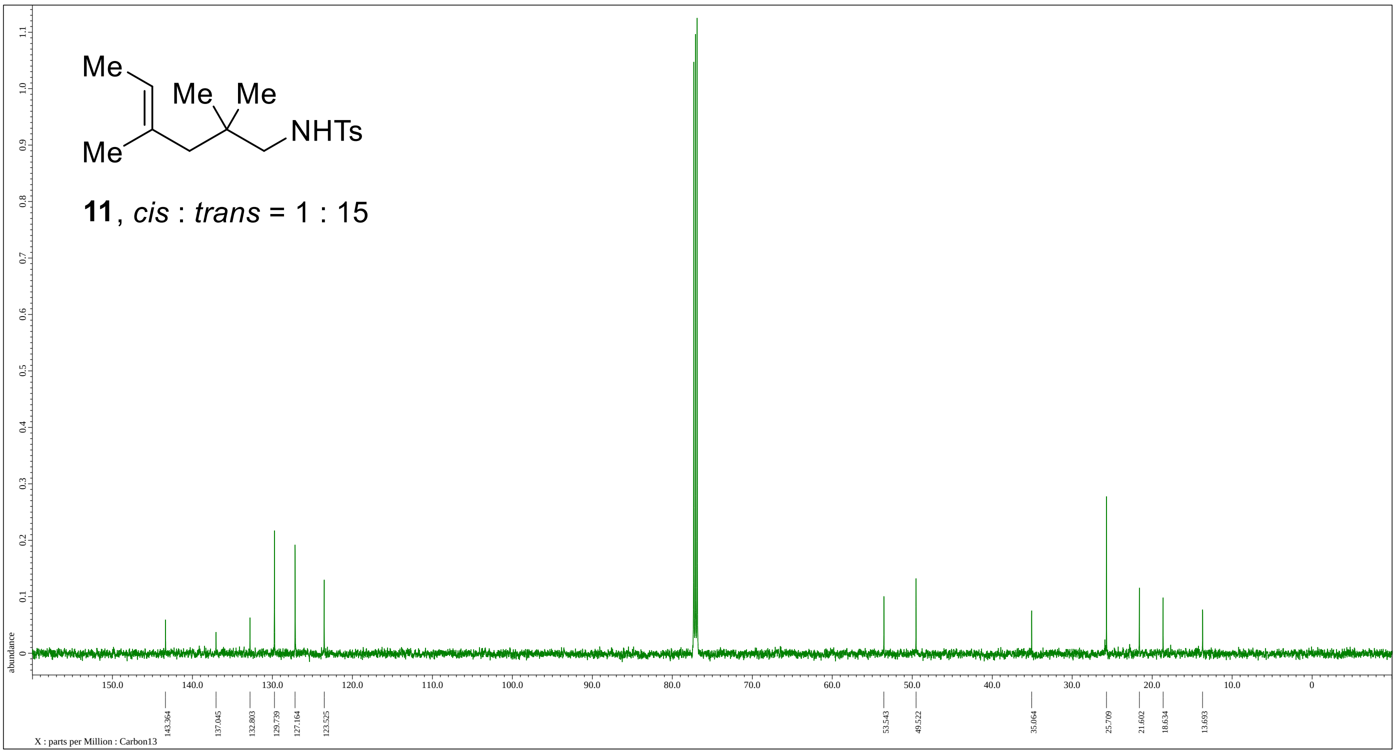
Me
Me Me
NHTs
Me
11, cis : trans = 1 : 15
abundance
X : parts per Million : Carbon13
143.364
137.045
132.803
129.739
127.164
123.525
53.543
49.522
35.064
25.709
21.602
18.634
13.693

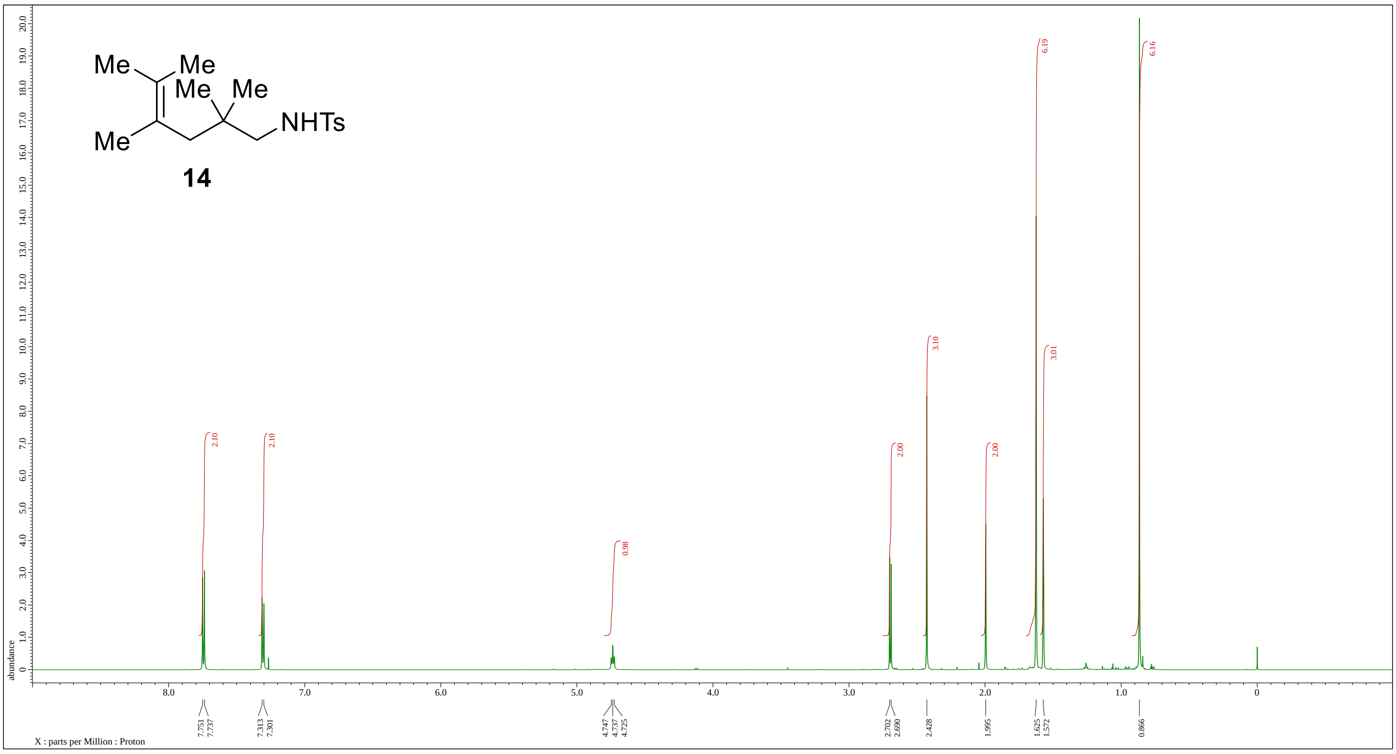
Me
Me
Me
Me
Me
NHTs
14
X : parts per Million : Proton
abundance

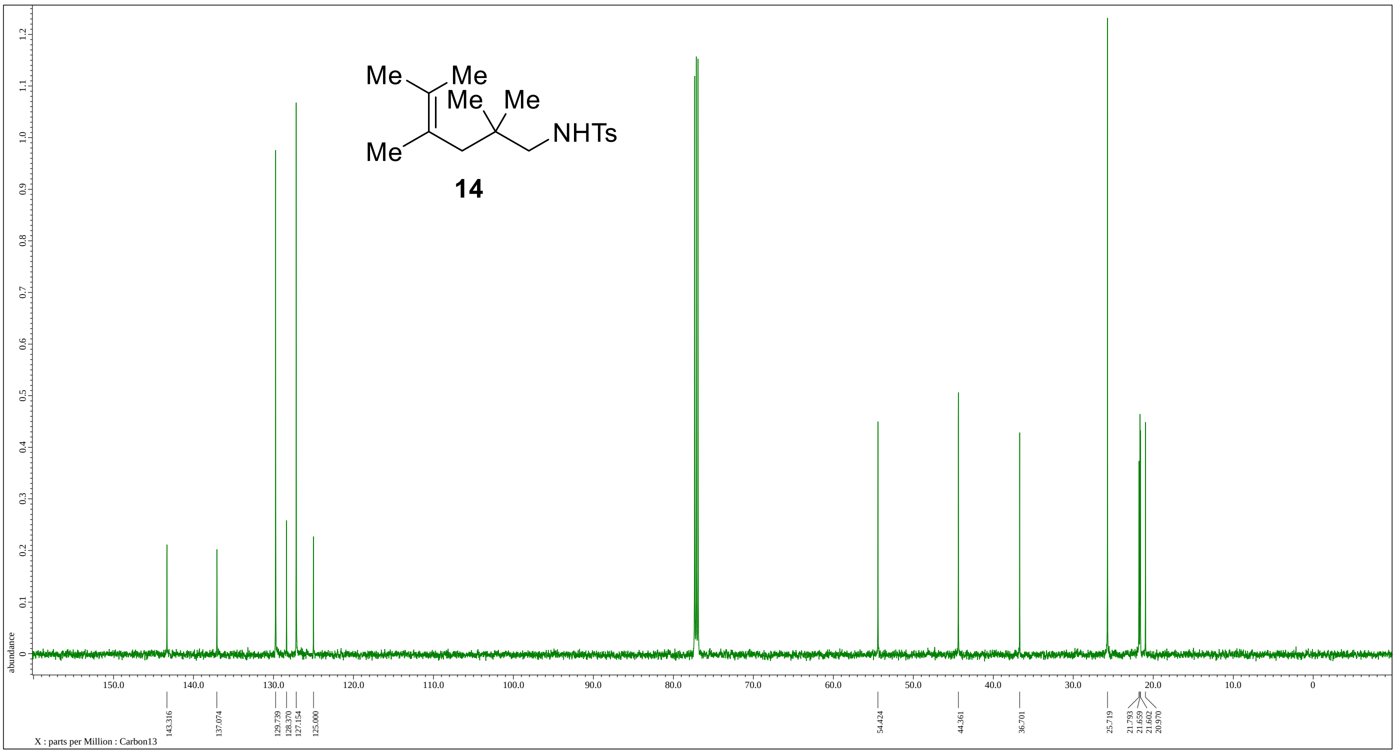
Me
Me
Me
Me
Me
NHTs
14
X : parts per Million : Carbon13
abundance

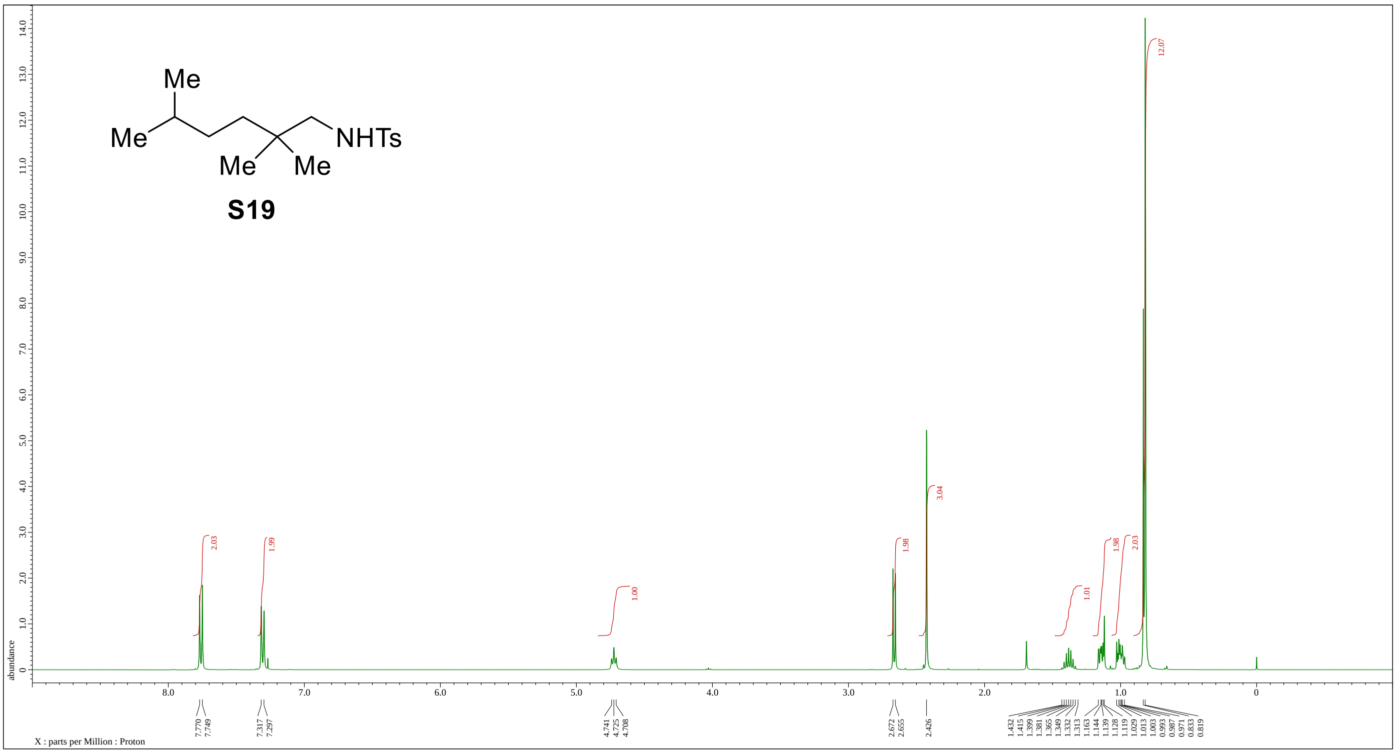
Me
Me
NHTs
Me
Me
S19
abundance
X : parts per Million : Proton
2.03
1.99
1.00
1.98
3.04
1.01
1.98
2.03
12.07
7.770
7.749
7.317
7.297
4.741
4.725
4.708
2.672
2.655
2.426
1.432
1.415
1.399
1.381
1.365
1.349
1.332
1.313
1.163
1.144
1.139
1.128
1.119
1.029
1.013
1.003
0.993
0.987
0.971
0.833
0.819

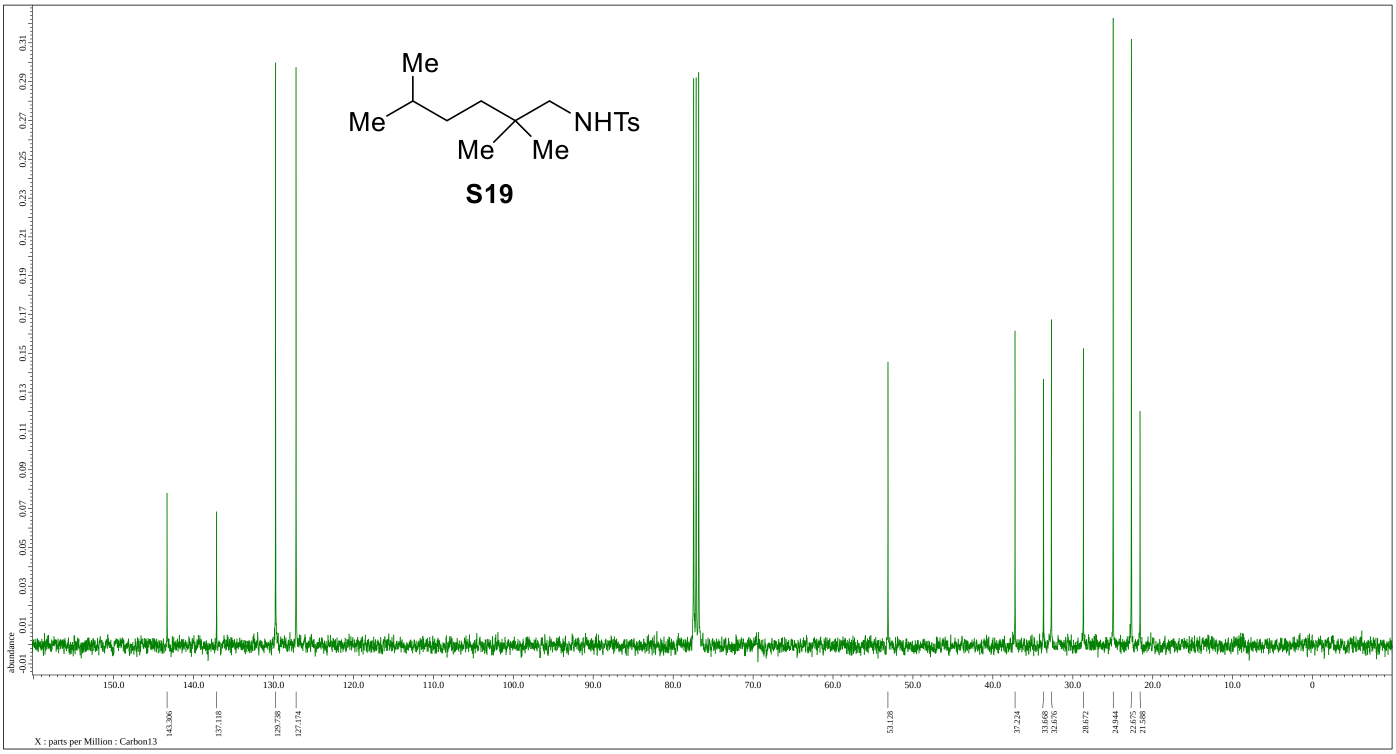
Me
Me
NHTs
Me
Me
S19
abundance
X : parts per Million : Carbon13
143.306
137.118
129.738
127.174
53.128
37.224
33.668
32.676
28.672
24.944
22.675
21.588

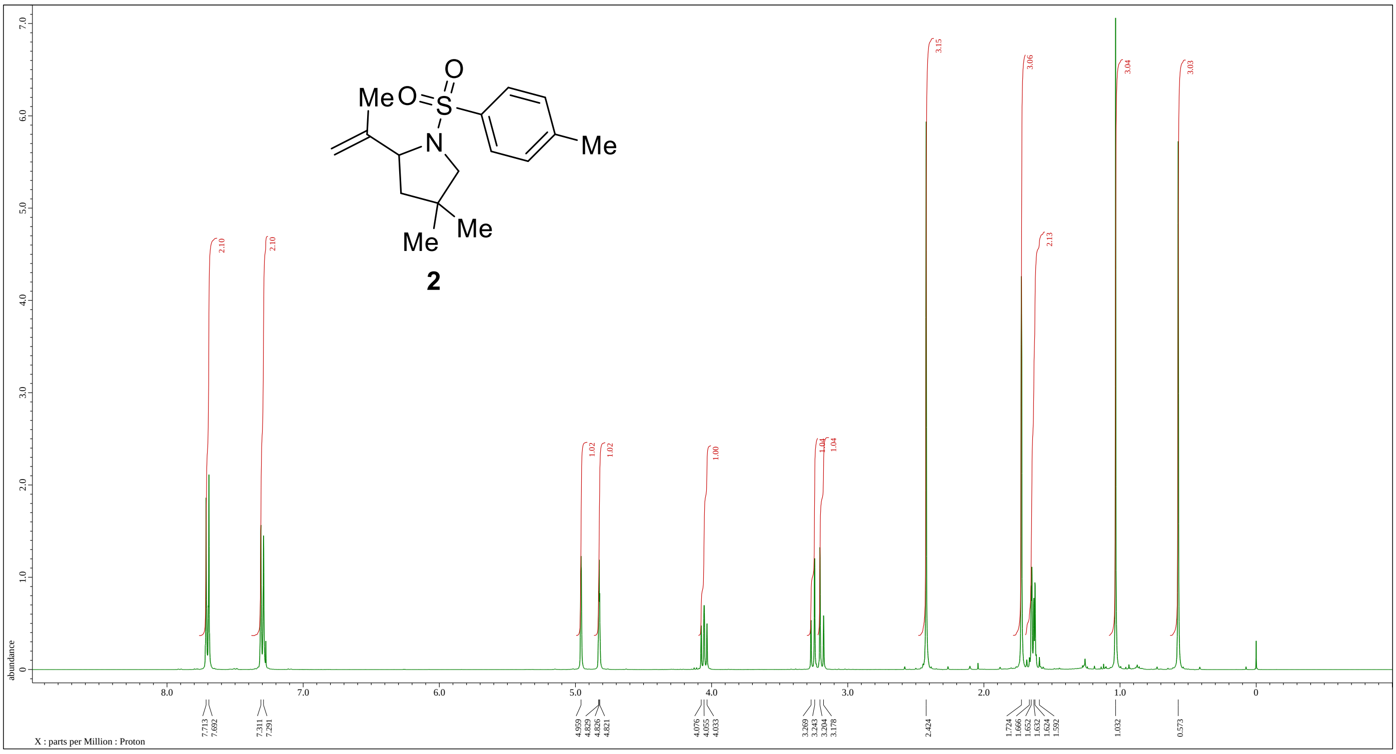

2
X : parts per Million : Proton
abundance


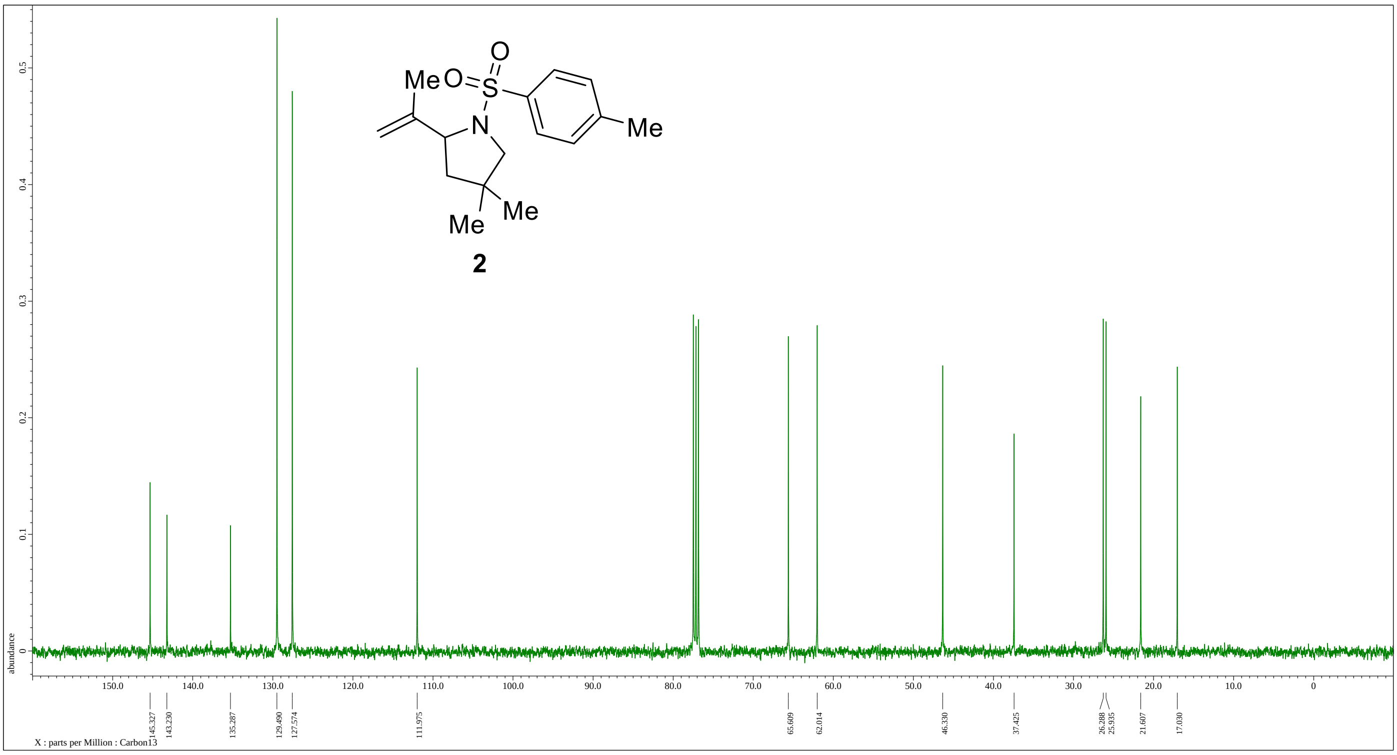

2
X : parts per Million : Carbon13
abundance

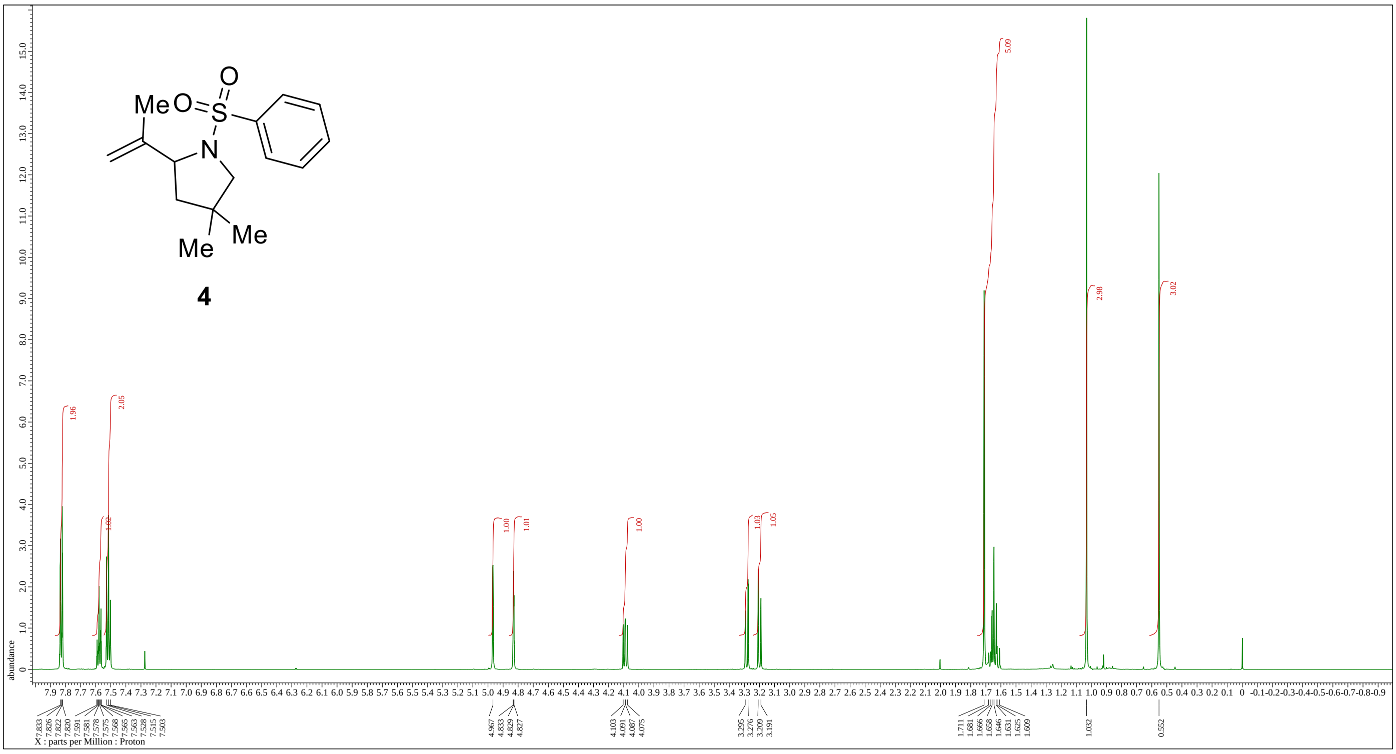

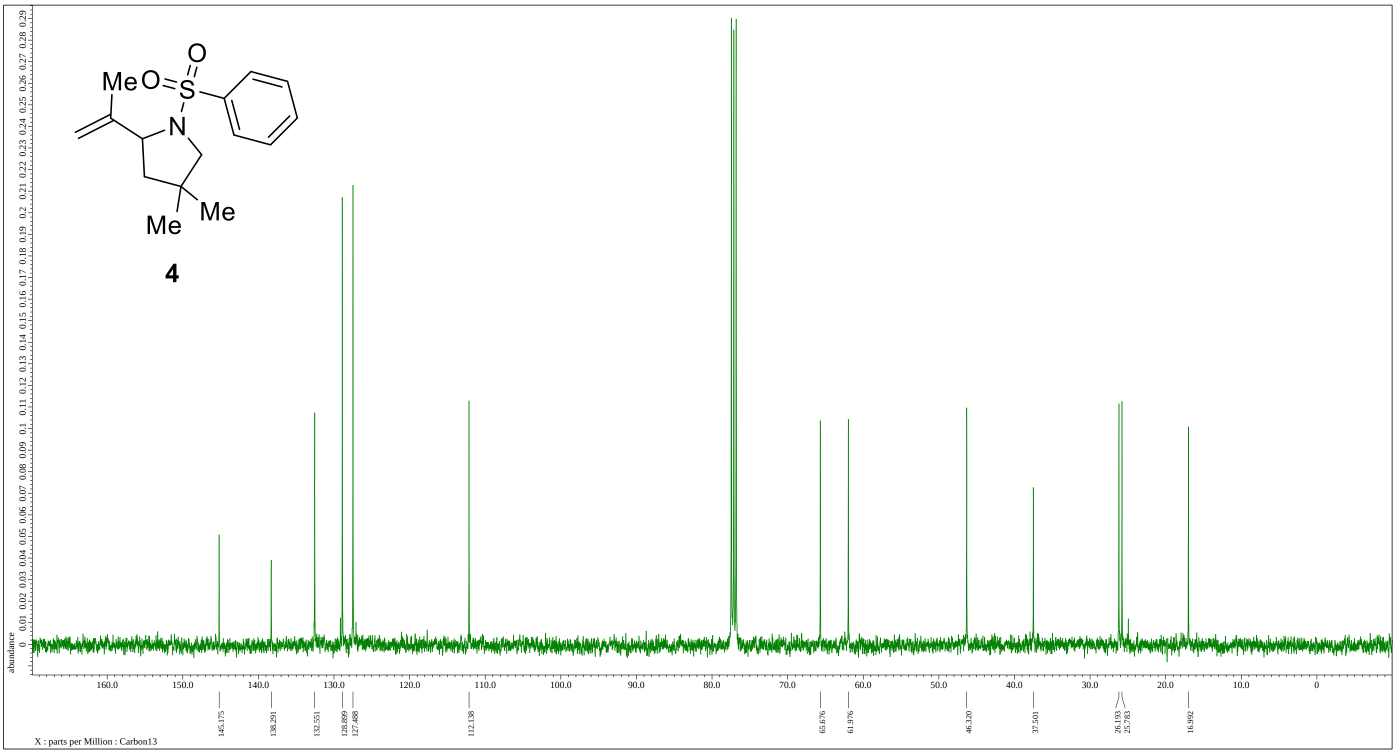

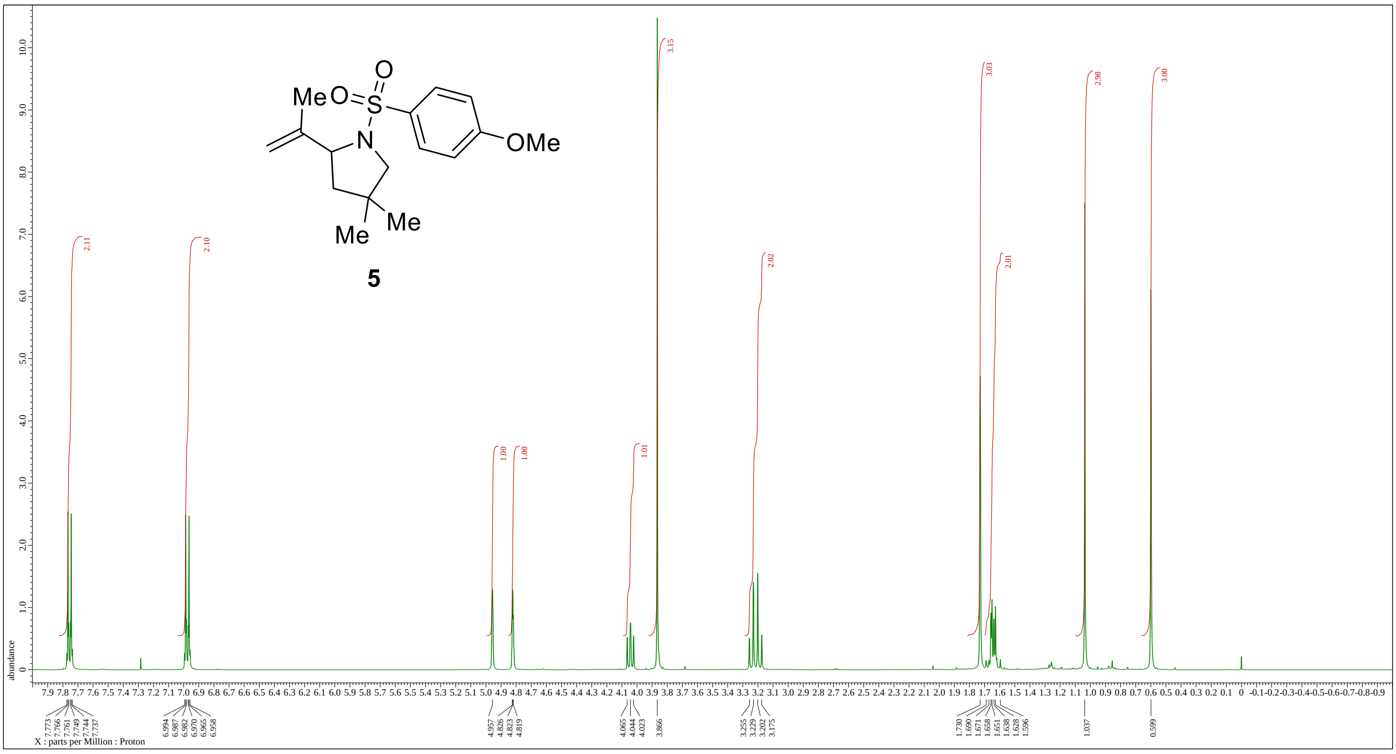

5
X : parts per Million : Proton
abundance


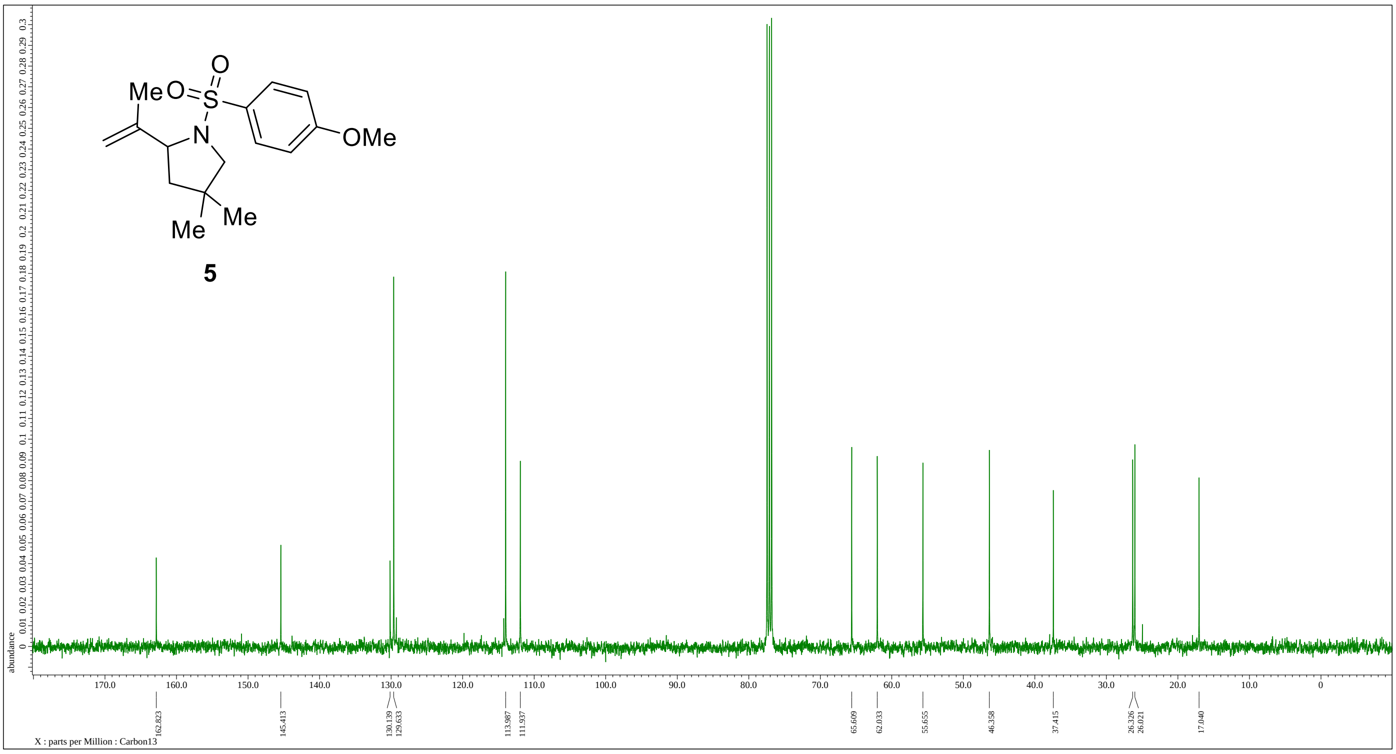

5
X : parts per Million : Carbon13
abundance

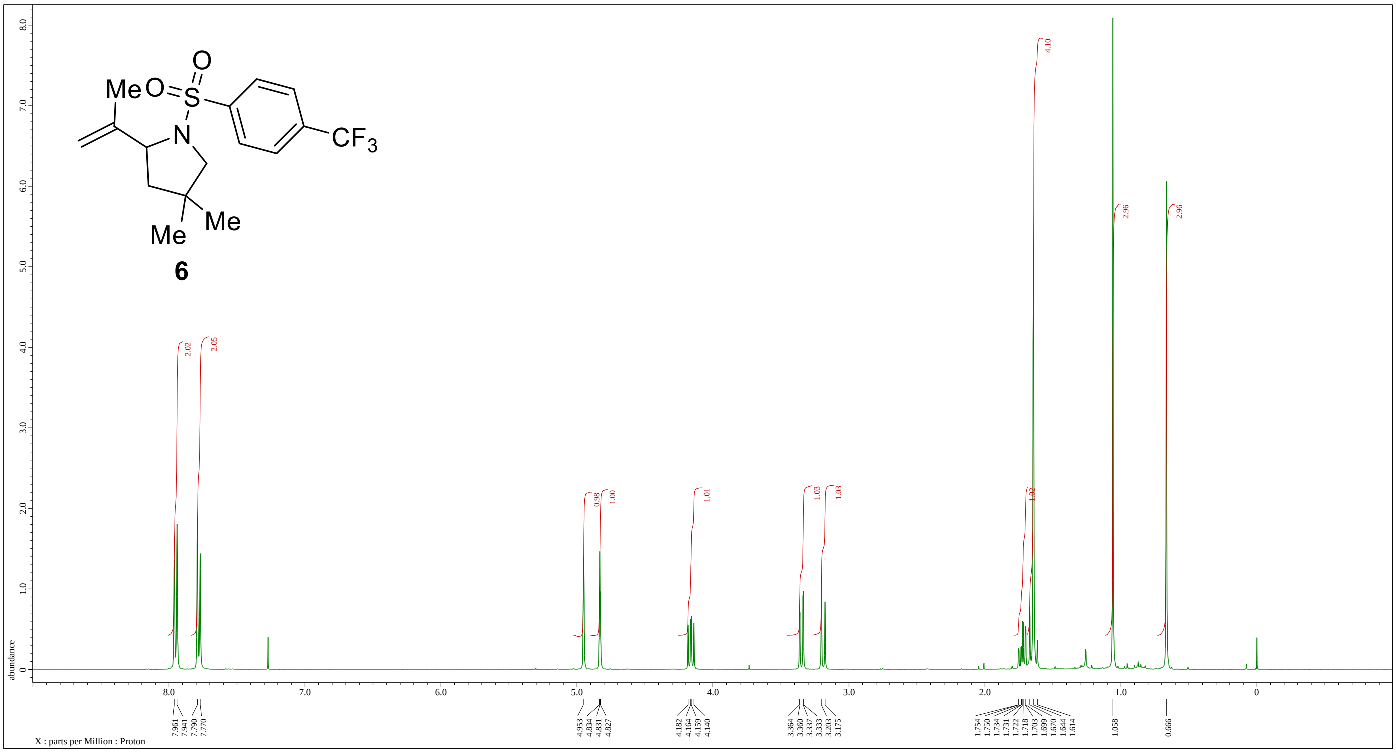
Me
O
O
S
N
CF3
Me
Me
6
X : parts per Million : Proton
abundance

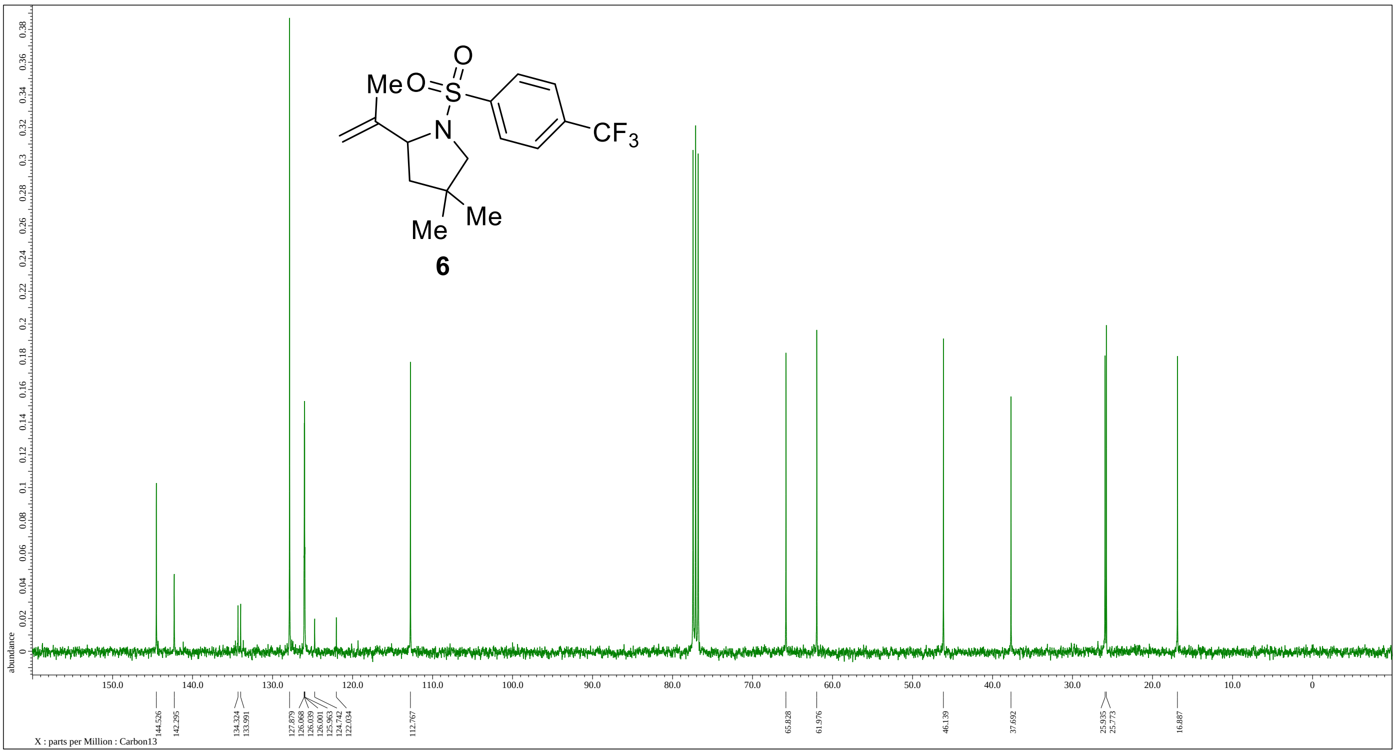
Me
O
O
S
N
CF3
Me
Me
6
X : parts per Million : Carbon13
abundance

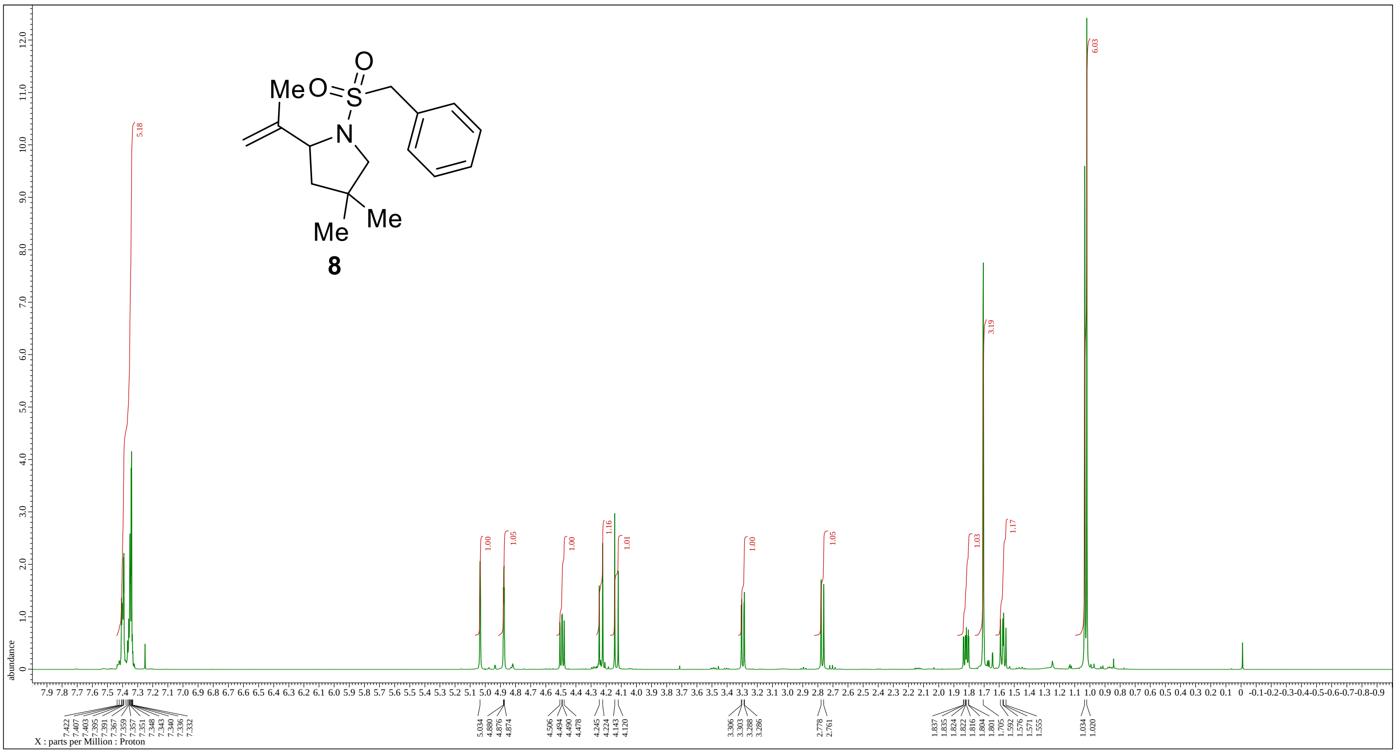
Me
O
S
N
Me
Me
8
abundance
5.18
1.00
1.05
1.00
1.16
1.01
1.00
1.05
1.03
3.19
1.17
6.03
7.422 7.407 7.403 7.395 7.391 7.367 7.359 7.357 7.351 7.348 7.343 7.340 7.336 7.332
5.034 4.880 4.876 4.874
4.506 4.494 4.490 4.478
4.245 4.224 4.143 4.120
3.306 3.303 3.288 3.286
2.778 2.761
1.837 1.835 1.824 1.822 1.816 1.804 1.801 1.795 1.592 1.576 1.571 1.555
1.034 1.020
X : parts per Million : Proton

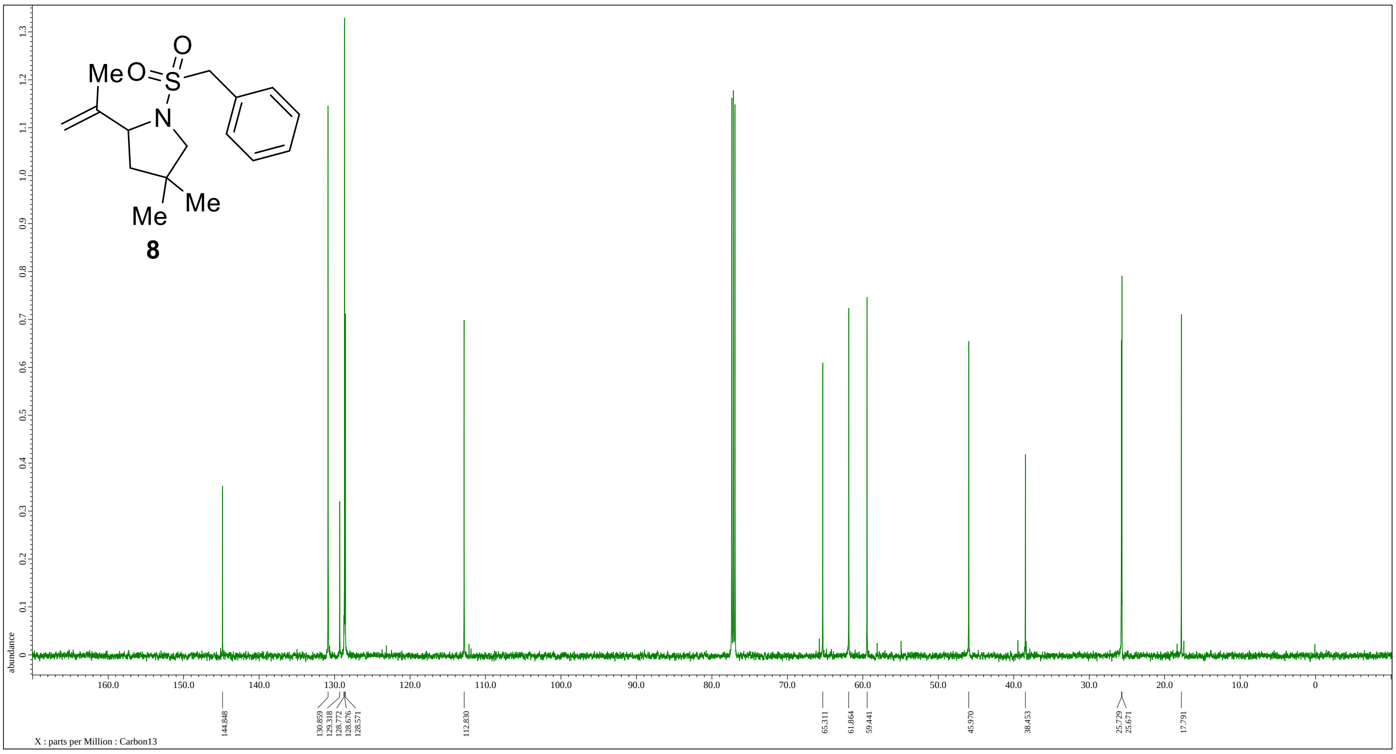
Me
O
S
N
Me
Me
8
abundance
144.848
130.859 129.318 128.772 128.676 128.571
112.830
65.311
61.864
59.441
45.970
38.453
25.729 25.671
17.791
X : parts per Million : Carbon13

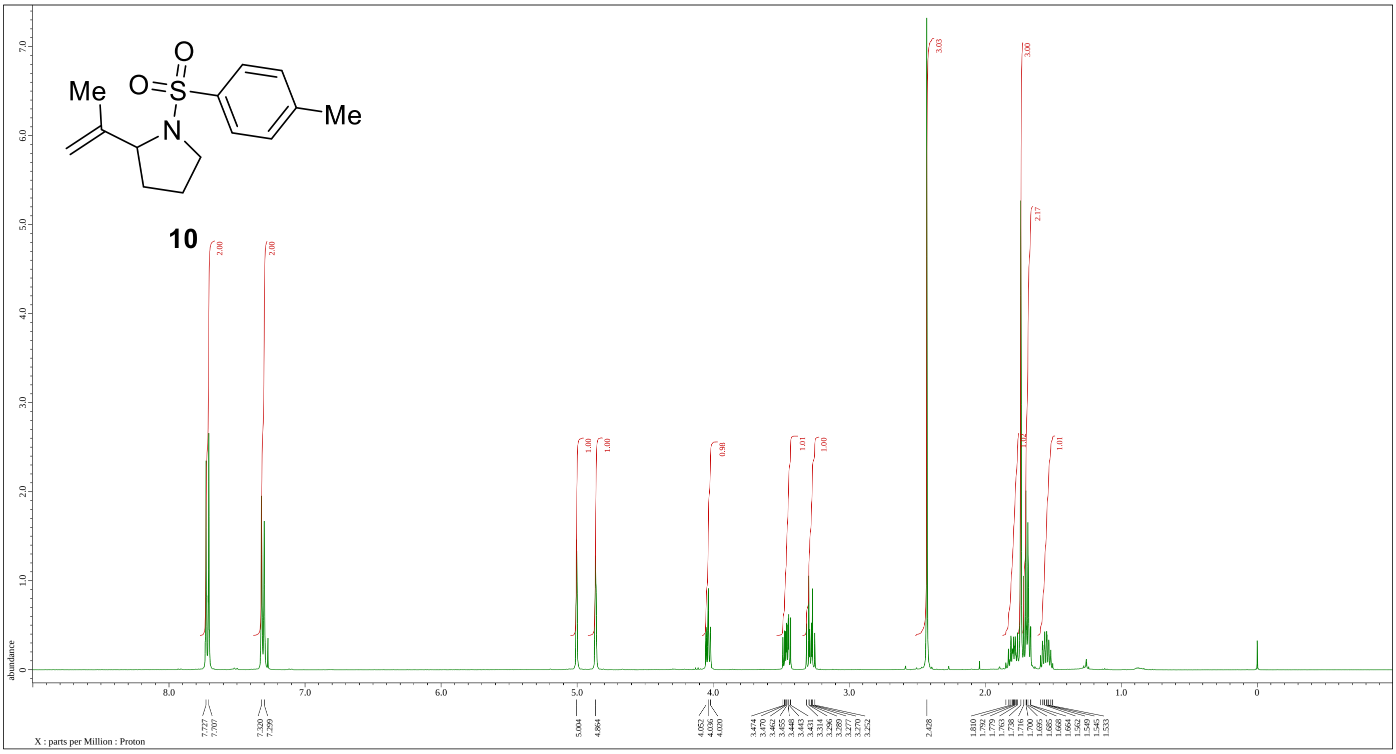

Me
O
O=S
N
Me
10
abundance
X : parts per Million : Proton
7.727
7.707
7.320
7.299
5.004
4.864
4.052
4.036
4.020
2.428
2.00
2.00
1.00
1.00
0.98
1.01
1.00
3.03
3.00
2.17
1.01


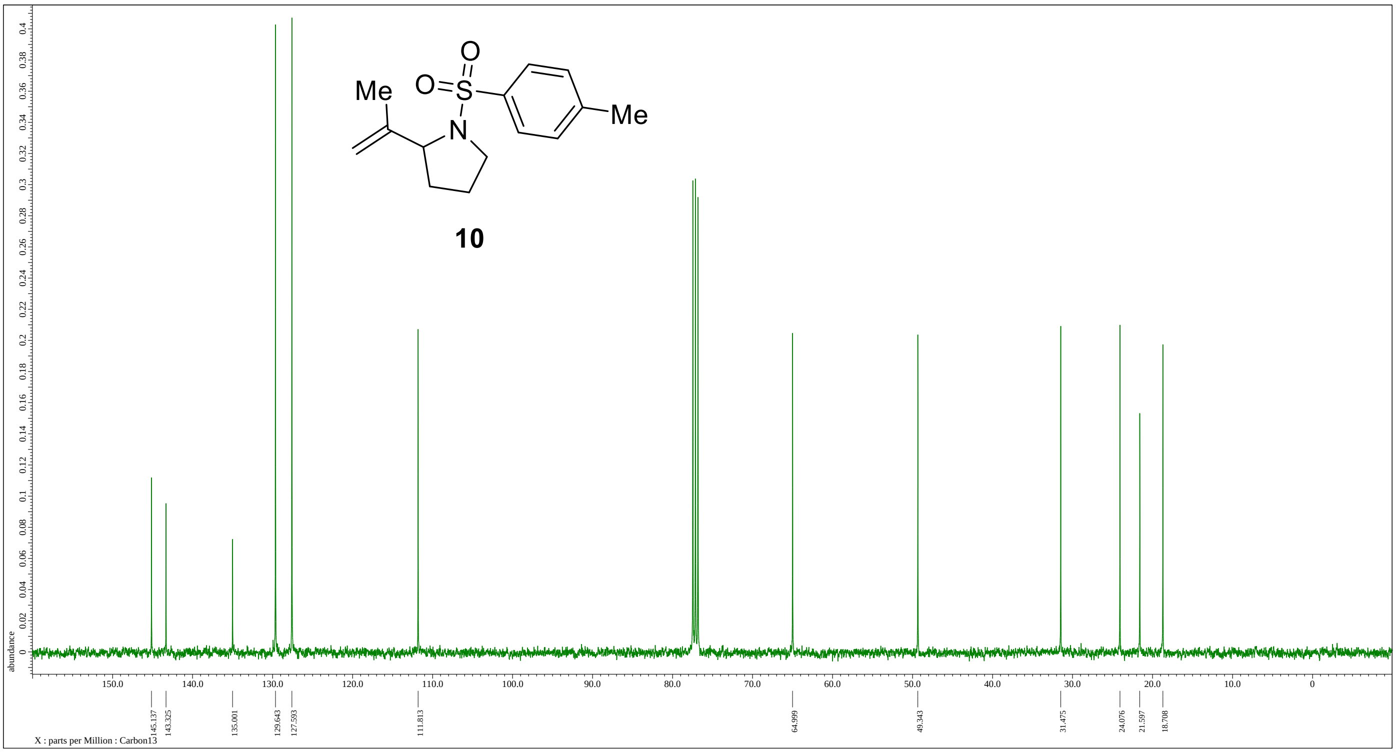

Me
O
O=S
N
Me
10
abundance
X : parts per Million : Carbon13
145.137
143.325
135.001
129.643
127.593
111.813
64.999
49.343
31.475
24.076
21.597
18.708

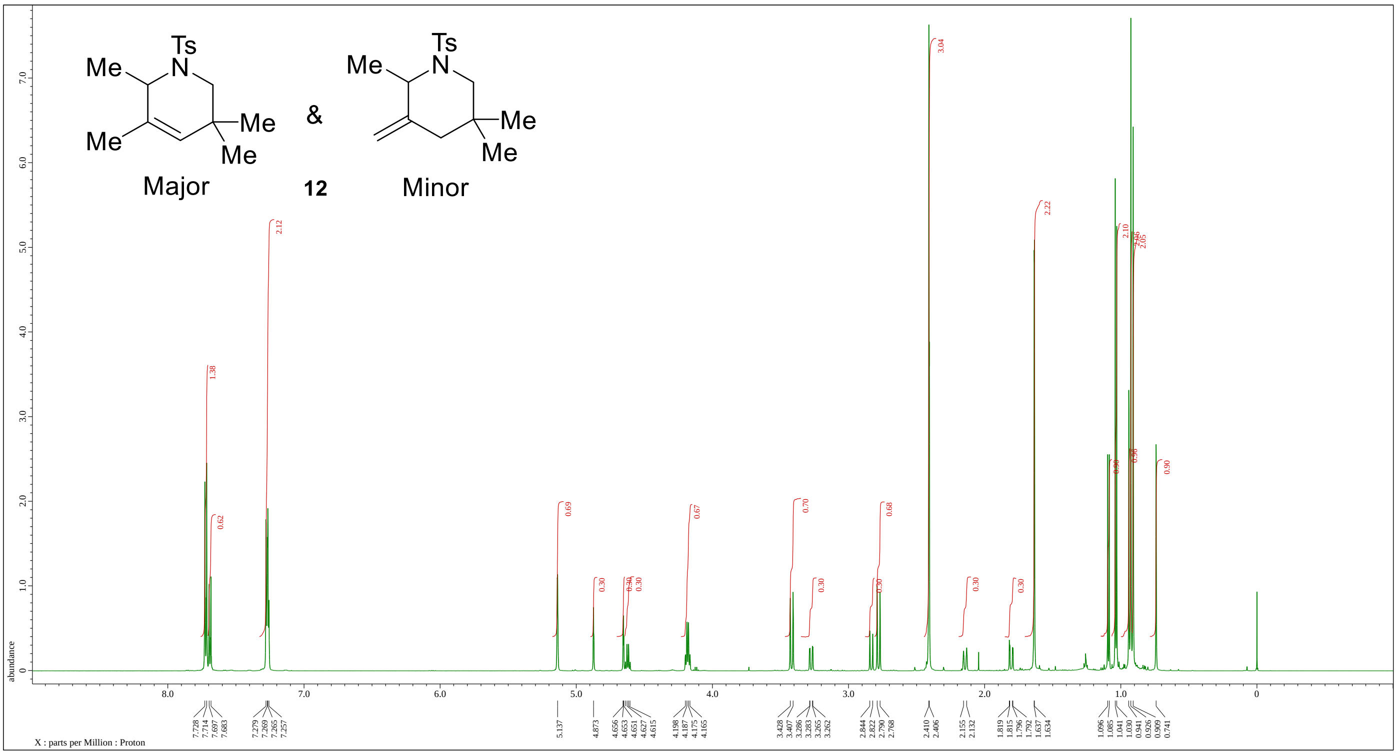
Major
12
Minor
X : parts per Million : Proton
abundance

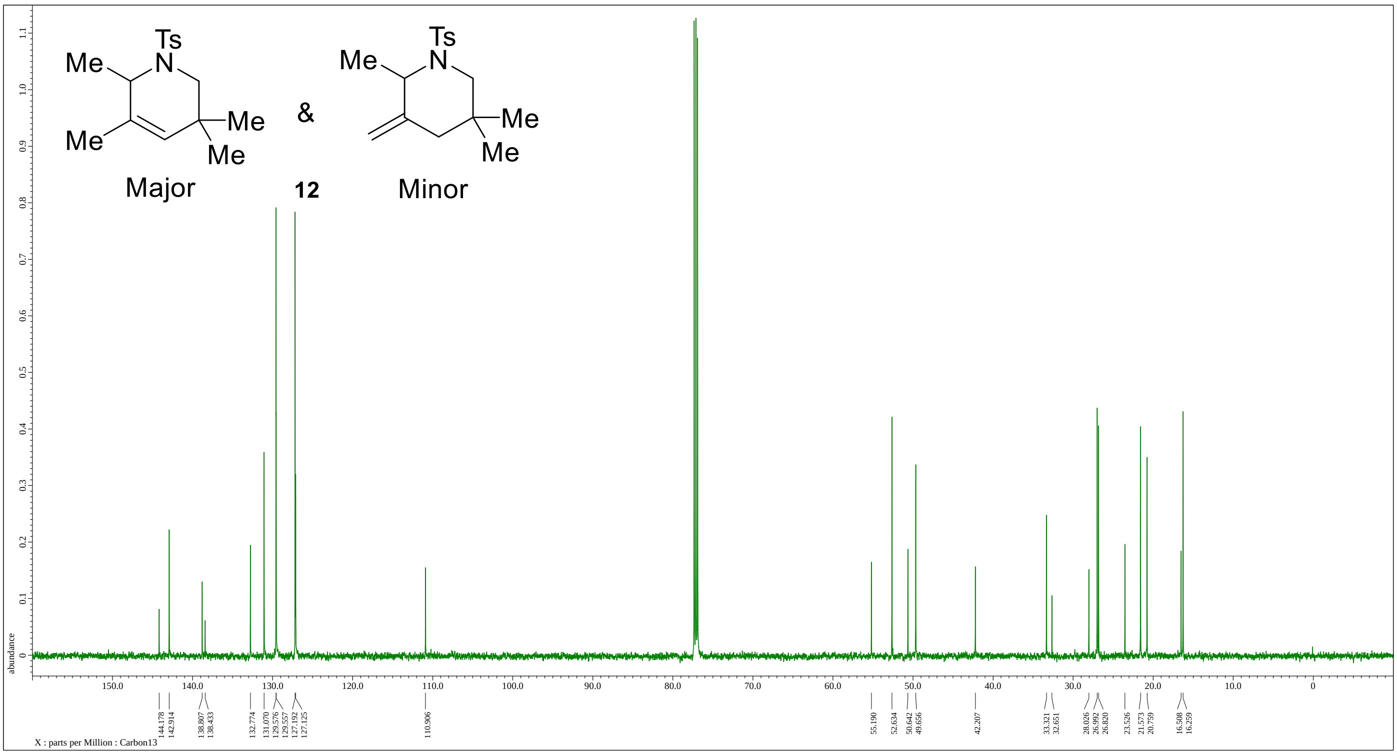
Major
12
Minor
X : parts per Million : Carbon13
abundance

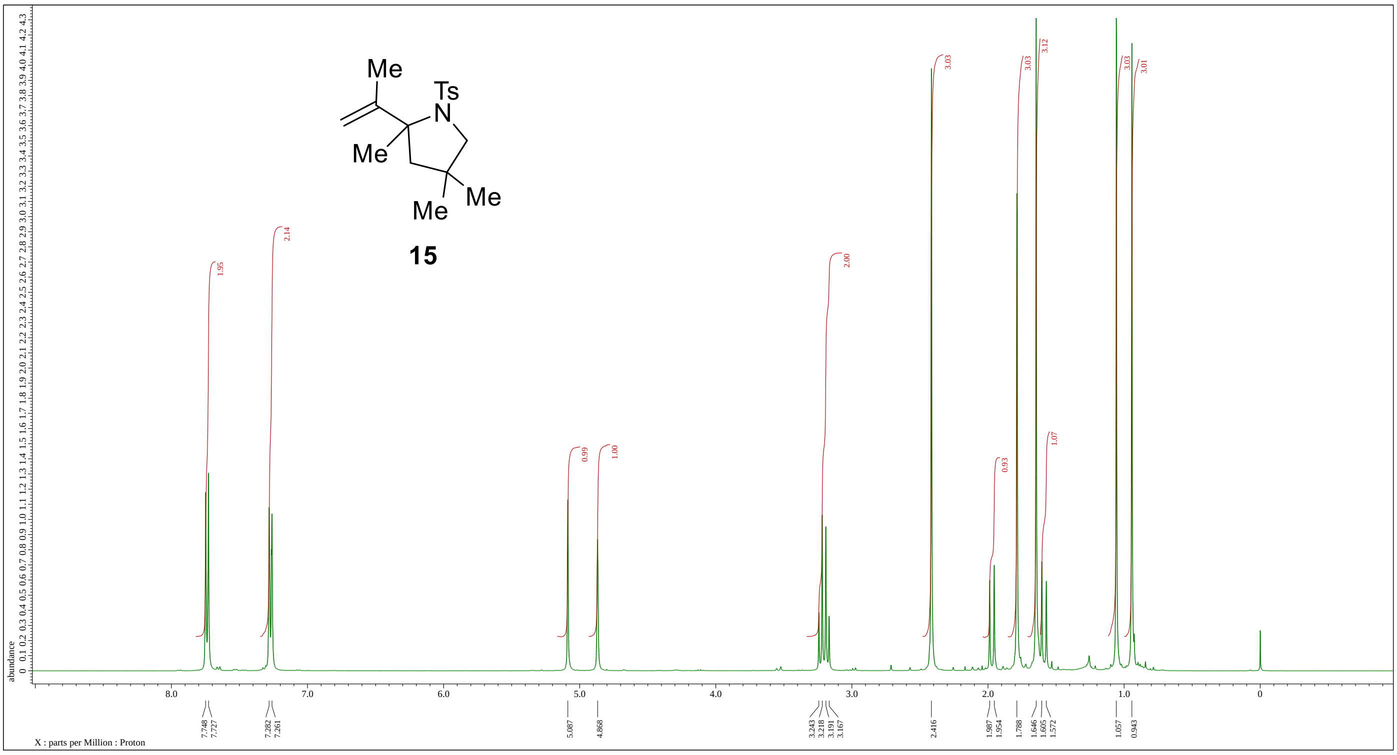
Me
Ts
N
Me
Me
Me
15
abundance
X : parts per Million : Proton
1.95
2.14
0.99
1.00
2.00
3.03
0.93
3.03
3.12
1.07
3.03
3.01
7.748
7.727
7.282
7.261
5.087
4.868
3.243
3.218
3.191
3.167
2.416
1.987
1.954
1.788
1.646
1.605
1.572
1.057
0.943

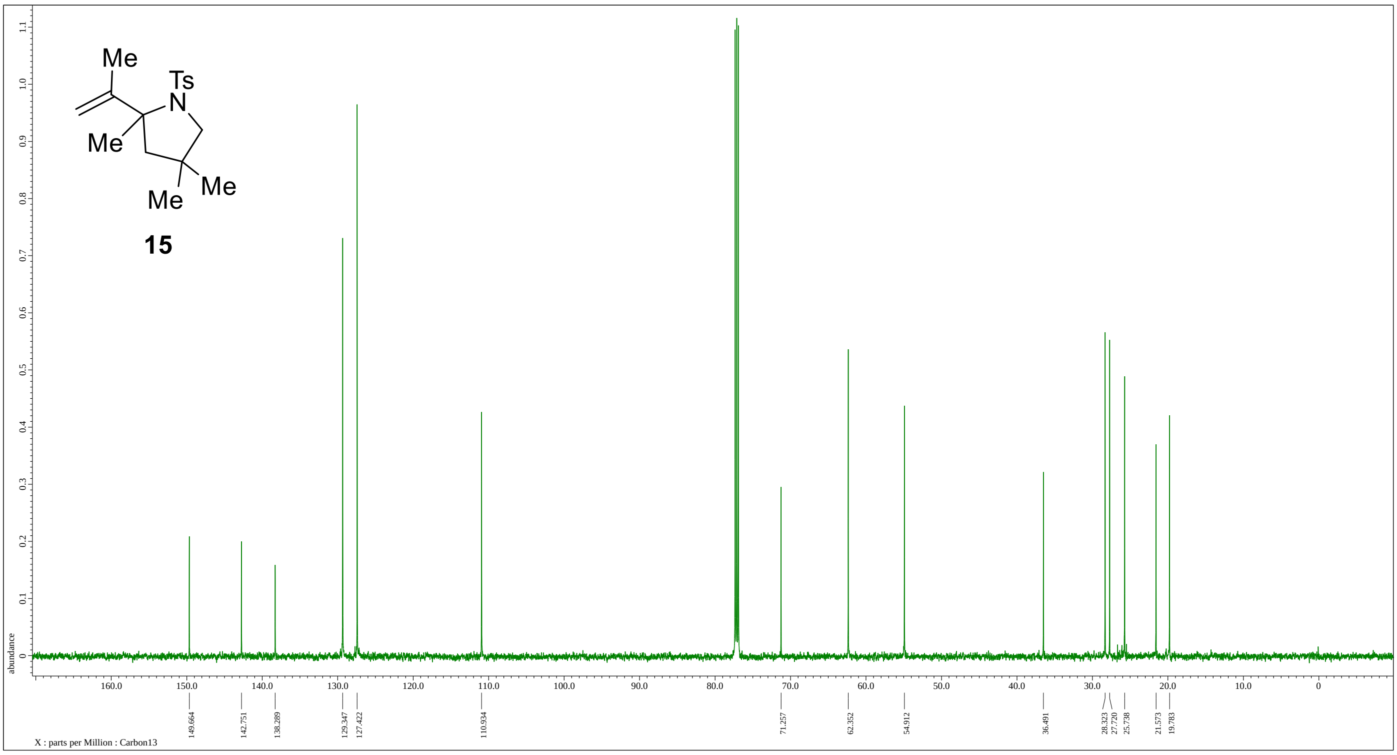
Me
Ts
N
Me
Me
Me
15
abundance
X : parts per Million : Carbon13
149.664
142.751
138.289
129.347
127.422
110.934
71.257
62.352
54.912
36.491
28.323
27.720
25.738
21.573
19.783

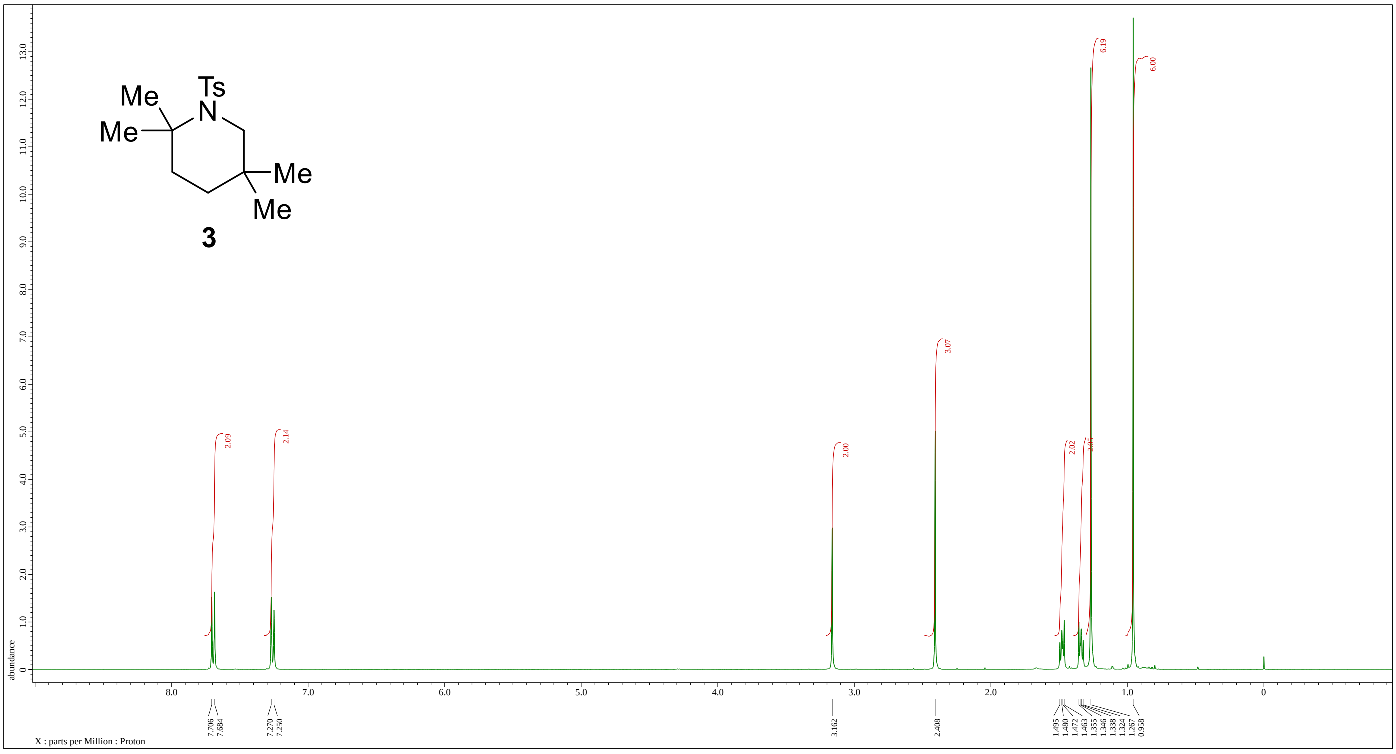
Me
Ts
N
Me
Me
Me
3
abundance
X : parts per Million : Proton
2.09
2.14
2.00
3.07
2.02
6.19
6.00
7.706
7.684
7.270
7.250
3.162
2.408
1.495
1.480
1.472
1.463
1.355
1.346
1.338
1.324
1.267
0.958

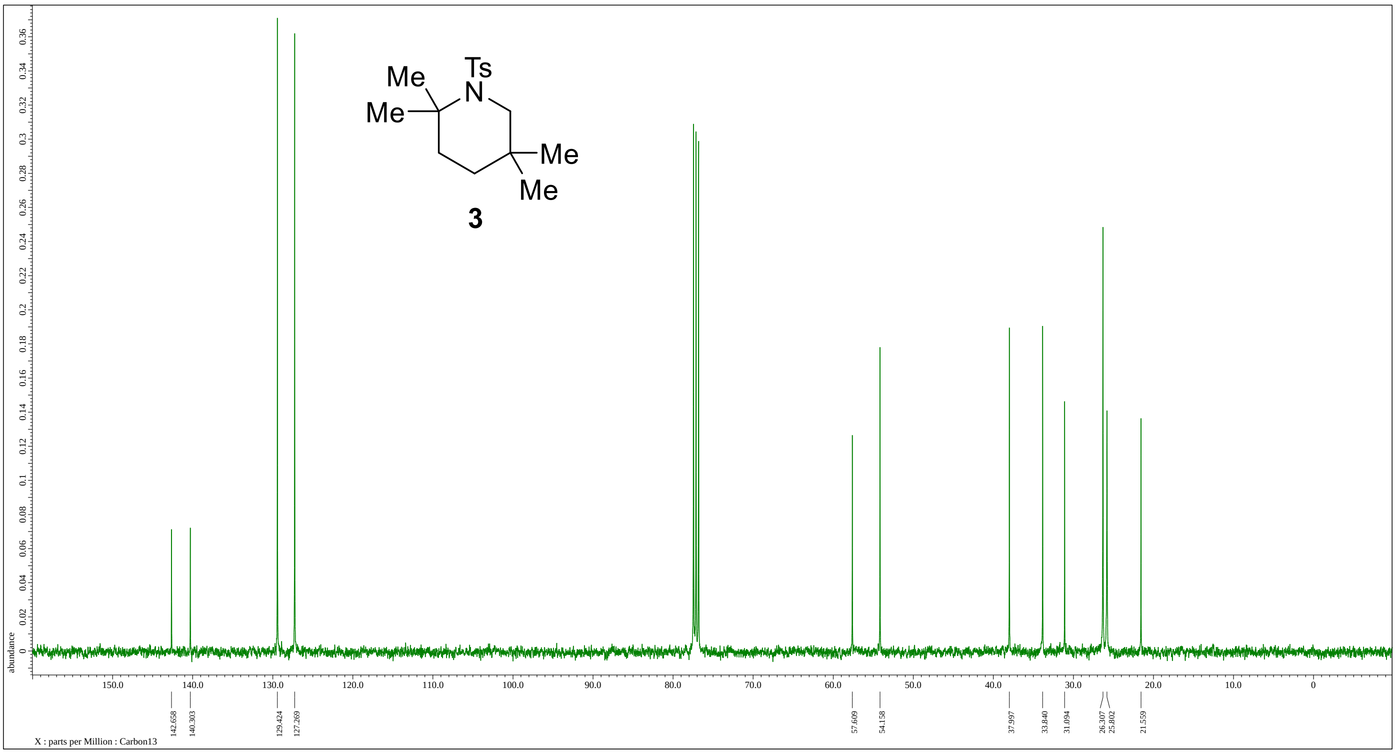
Me
Ts
N
Me
Me
Me
3
abundance
X : parts per Million : Carbon13
142.658
140.303
129.424
127.269
57.609
54.158
37.997
33.840
31.094
26.307
25.802
21.559

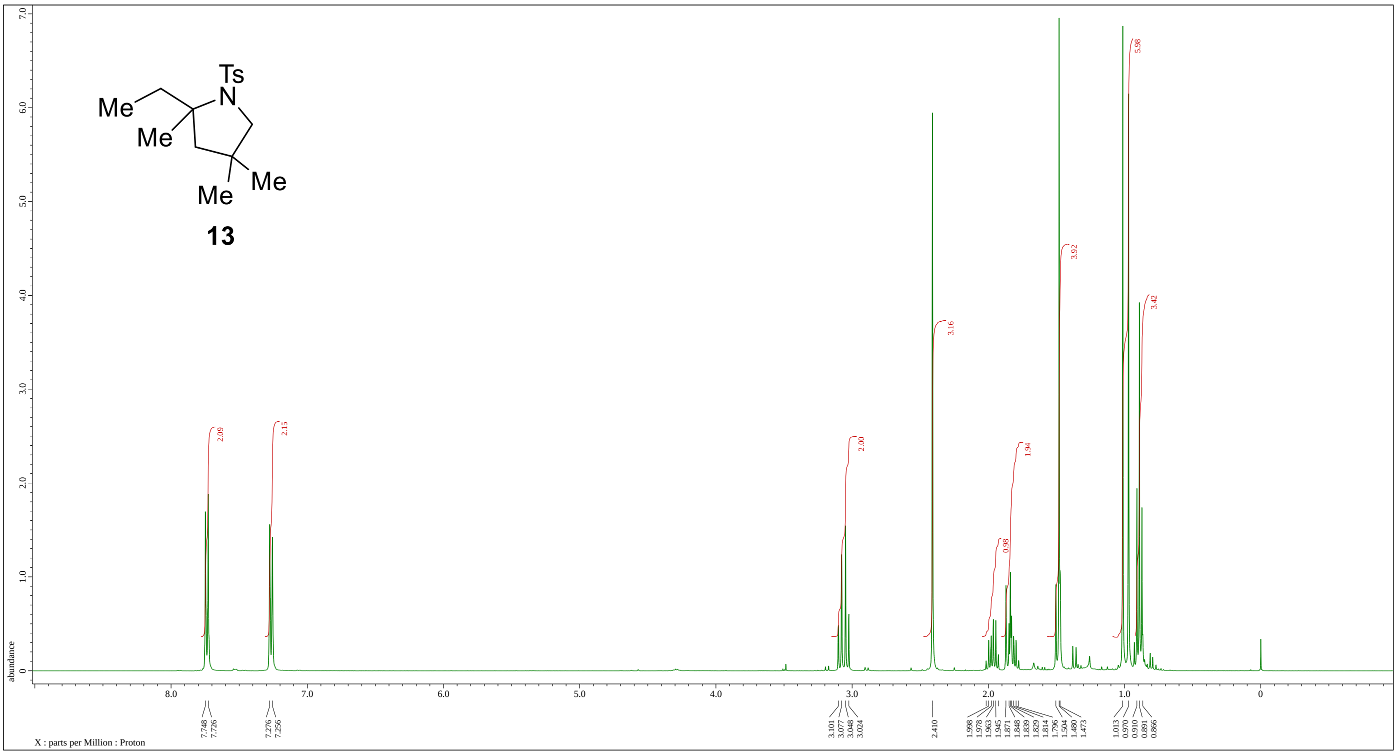
Ts
N
Me
Me
Me
Me
13
abundance
X : parts per Million : Proton
7.748
7.726
7.276
7.256
3.101
3.077
3.048
3.024
2.410
1.998
1.978
1.963
1.943
1.871
1.848
1.839
1.829
1.814
1.796
1.504
1.480
1.473
1.013
0.970
0.910
0.891
0.866
2.09
2.15
2.00
3.16
0.98
1.94
3.92
5.98
3.42

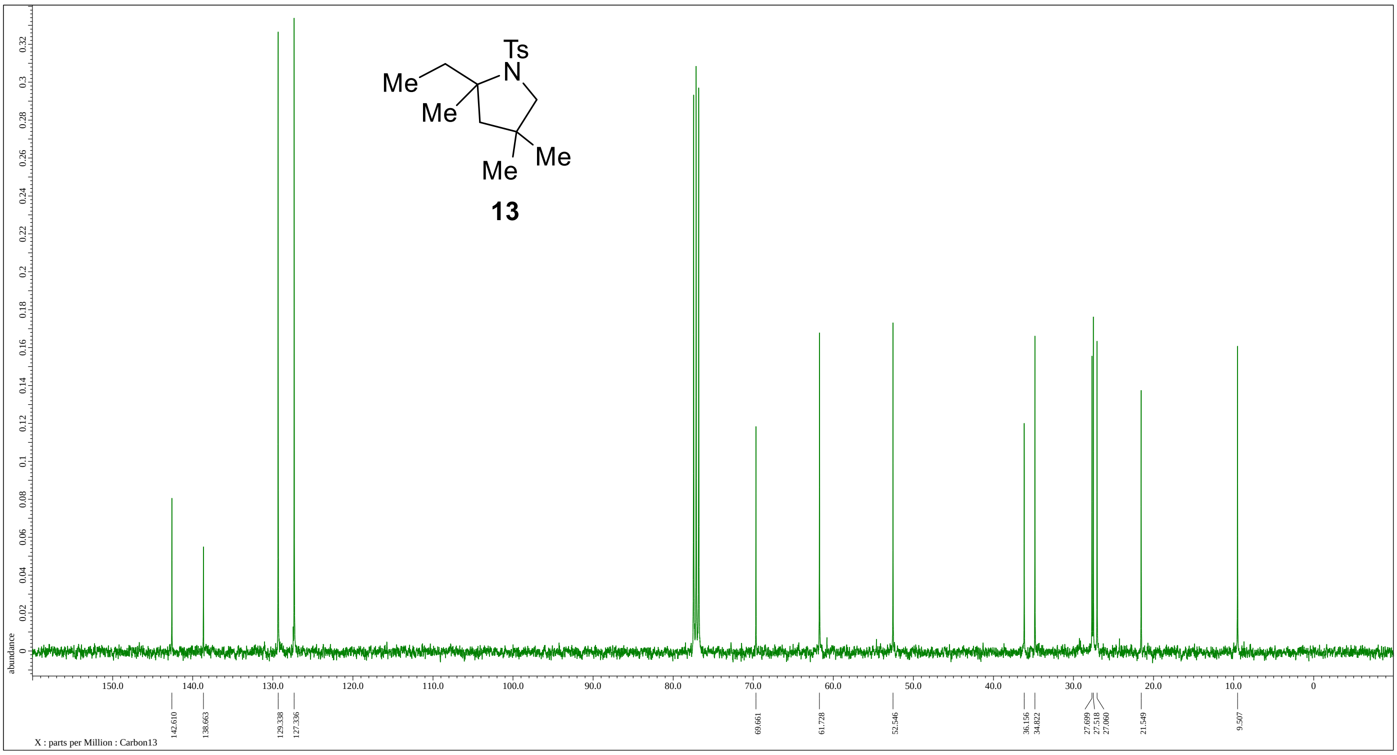
Ts
N
Me
Me
Me
Me
13
abundance
X : parts per Million : Carbon13
142.610
138.663
129.338
127.336
69.661
61.728
52.546
36.156
34.822
27.699
27.518
27.060
21.549
9.507

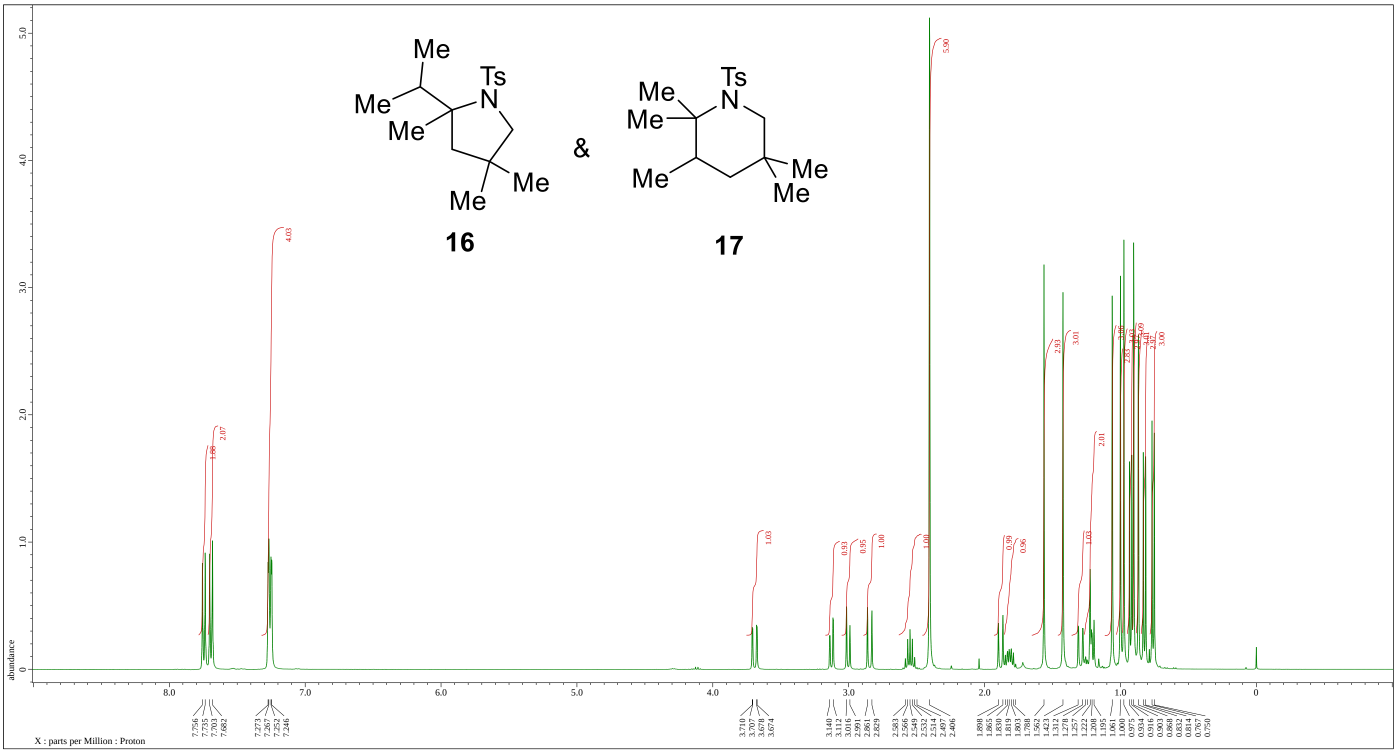

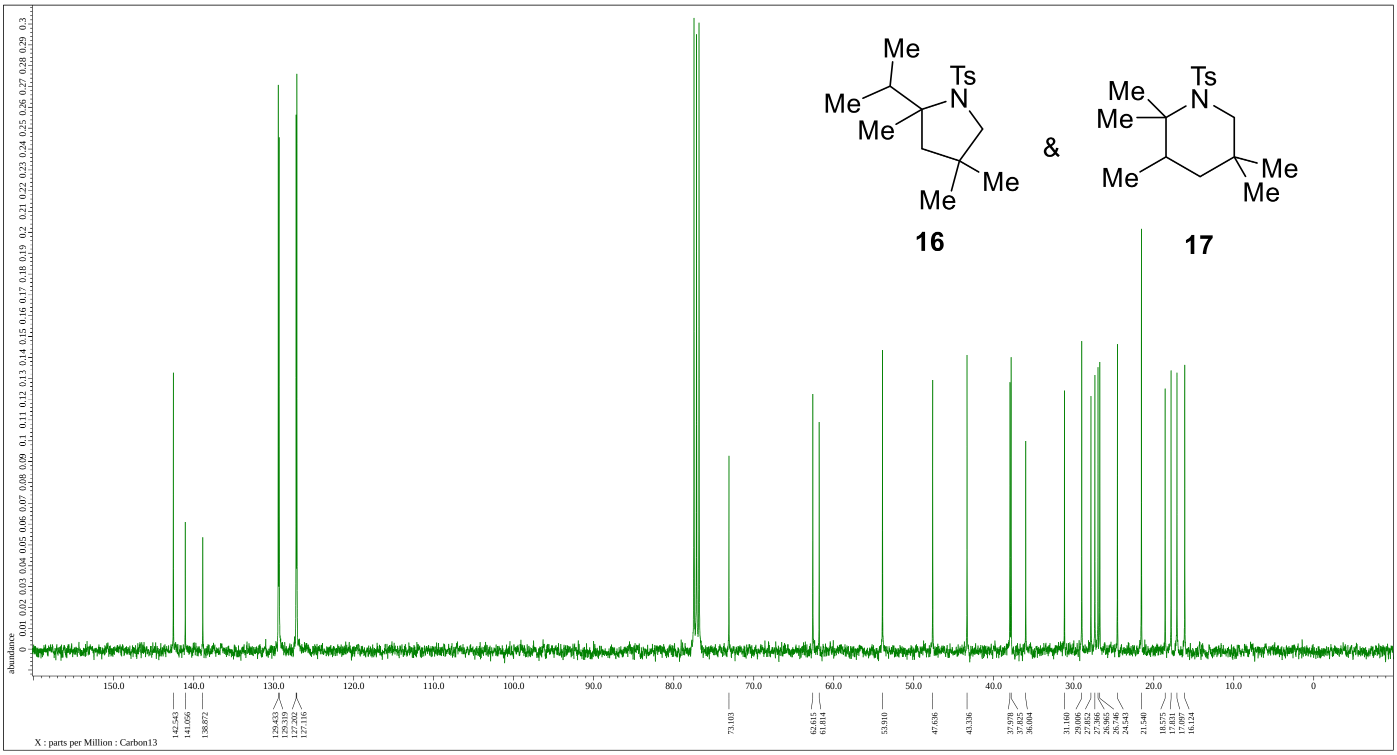

Supplement: File 1 — General remarks and characterization data, including copies of 1H and 13C NMR spectra. [file Beilstein_J_Org_Chem-20-1900-s001.pdf]
